# The determinants of trust: findings from large, representative samples in six OECD countries

**DOI:** 10.1111/ecca.12549

**Published:** 2024-08-29

**Authors:** Roxanne Kovacs, Maurice Dunaiski, Matteo M. Galizzi, Gianluca Grimalda, Rafael Hortala-Vallve, Fabrice Murtin, Louis Putterman

**Affiliations:** 1 University of Gothenburg; 2 LSE; 3 Kiel Institute for the World Economy; 4 OECD Centre on Well-being, Inclusion, Sustainability and Equal Opportunity; 5 Brown University

**Keywords:** D9, C91, D01

## Abstract

Trust is key for economic and social development. But why do we trust others? We study the motives behind trust in strangers using an experimental trust game played by 7236 participants, in six samples representative of the general populations of Germany, Italy, Japan, Luxembourg, the UK and the USA. We examine the broadest range of potential determinants of trustor sending to date, including risk tolerance, preferences for redistribution, and conformity. We find that even though self-interest, indicated by expected returns, is relevant for trustor behaviour, the most important correlate of sending is participants’ altruism or fairness concerns, as measured by giving in a dictator game. We also find that in our large and representative sample, behaviour in the trust game and responses in a trust survey are significantly correlated, and that similar correlates—altruism in particular—are relevant for both.

## 1 │ INTRODUCTION

Trust is crucial for economic and social development, as it facilitates cooperation, information sharing and entrepreneurial activity (Arrow 1972; Putnam 1993; Durlauf 2002; Dearmon et al. 2009; Fehr 2009; Francois and Zabojnik 2005). But why do we trust others? And why are we trustworthy? It is generally assumed that trust is based primarily on self-interested motives (Coleman 1994; Algan and Cahuc 2013). For instance, according to Gambetta (1988), when we say that we trust someone, ‘we implicitly mean that the probability that he will perform an action that is beneficial or at least not detrimental to us is high enough for us to consider engaging in some form of cooperation with him’. The interdisciplinary definition offered by Rousseau et al. (1998), which begins by saying ‘Trust is a psychological state comprising the intention to accept vulnerability’ likewise continues by stating that the intention is ‘based upon positive expectations of the intentions or behaviour of another’. Trustworthiness is our willingness to act favourably towards others when an implicit or explicit demand for action has been placed on us (Ben-Ner and Halldorsson 2010). Trustworthiness is often assumed to be motivated primarily by reciprocity—that is, the tendency to reward cooperative behaviour and to punish uncooperative behaviour (Fehr and Falk 2002). The assumption that trust and trustworthiness are determined primarily by expectations of return, for the trustor, and by obligations of reciprocity, by trustees, is common not only in economics but also in a range of other disciplines, including political science (Hardin 2002), philosophy (Dietz 2011) and psychology (Rotter 1980).

We study the determinants of both self-reported and incentivized trust in strangers using data from the Trustlab survey. Trustlab was designed to study trust in other individuals as well as in institutions and government, through a combination of incentivized games and survey questions. Specifically, we examine behaviour in an experimental trust game played by a sample of 7236 participants, in six samples representative of the general adult populations of Germany, Italy, Japan, Luxembourg, the UK and the USA. The trust game (or investment game) is a simple decision task, where one participant (the trustor, or first mover) is given a monetary endowment and can decide which proportion (if any) they want to send to a second participant (the trustee, or second mover). If a transfer is made, then the amount is multiplied by a number greater than 1, and the trustee can decide which proportion to return to the trustor. The amount of money transferred by the first mover has been used to measure trust, and the proportion of the available amount returned has been used to measure trustworthiness, although Cox (2004) and Carter and Castillo (2011) note correctly that part of the sending in both directions might be attributed to other-regarding preferences. Although trust is also measured in surveys (Knack and Keefer 1997), many experimental and behavioural economists have preferred the trust game as a method for measuring trust (Berg et al. 1995; Glaeser et al. 2000). The main reason for this is that behaviour in the trust game has monetary consequences, making it *a priori* less likely that participants should deviate from their true preferences (Glaeser et al. 2000). We study the motives that underlie first-mover sending in a trust game, and investigate the role of expected returns, altruism or fairness preferences (based on a dictator game), and risk preferences (based on a multiple lotteries task), as well as a large number of other personal characteristics, values and attitudes—including preferences for redistribution and conformity.

In our samples in Germany, Italy, Japan, Luxembourg, the UK and the USA, trustors send an average of 60% of their endowment to trustees, and trustees return an average of 34% of the available amount, resembling behaviours in a large meta-analysis of 84 experiments (averages 50% and 37%, respectively, in Johnson and Mislin 2011). Cross-country differences in average sending and return proportions in our study are relatively small. We find that self-interest, indicated by expected return by the receiver, as well as risk aversion, arguably a self-protective preference, is a significant predictor of the amount sent as trustor. However, the most important correlate of trustor sending—predicting 13% of behavioural variation—is their sending in a dictator game, conventionally an indicator of altruism or fairness preferences. We find that the sender’s gender and religion, as well as their preferences for redistribution and conformity, are associated with the amount sent, although the share of variation explained is small. We also analyse the correlates of the trustees’ transfers. Results suggest that second-mover behaviour is correlated with reciprocity (proxied by conditional cooperation, based on a conditional public goods game)—which is particularly important when small amounts are received. However, the analysis also indicates that dictator-game sending explains a larger share of the variance in second-mover behaviour than any other factor (i.e. 10%).

Our study makes several contributions. First, it contributes to the literature on the determinants of sending behaviour in the trust game (Ashraf et al. 2006; Schechter 2007; Holm and Danielson 2005; Sapienza et al. 2013; Dietz 2011). To our knowledge, our study is the first to investigate such a broad range of potential correlates of first- and second-mover transfers towards unidentified strangers in large and representative samples of adult participants in several countries, using both experimental and survey methods. The games and survey questions in the Trustlab platform permit us to investigate the broadest range of potential determinants of trustor sending in the trust game to date, including risk preferences, preferences for redistribution and conformity. This therefore provides a more comprehensive picture of what drives trust and trustworthiness, extending previous findings from small-scale studies, for example: by Ashraf et al. (2006), who examine the role of expected returns, altruism and risk preferences in a sample of 168 university students; by Sapienza et al. (2013), who study the role of expected returns, conditional cooperation and risk preferences in a sample of 554 university students; and by Schechter (2007), who focuses on the role of risk aversion with 188 villagers in Paraguay.

We also contribute to the literature that examines the correlation between trust games and trust surveys (Glaeser et al. 2000; Etang et al. 2012; Holm and Nystedt 2008; Banerjee et al. 2021). We find that in our large representative samples, behaviour in the game and responses in the survey are significantly and positively correlated. This correlation was observed despite the fact that there was no connection between the game and the survey question: the two were presented in methodologically distinct modules and in temporally separated stages of the survey process, using different wording. Our results also suggest that the types of factors that predict sending behaviour in the game—altruism in particular—are also relevant for predicting responses to the trust survey question.

Finally, our study helps to introduce the methodological innovation of Trustlab, a platform that combines a large survey module with a multi-part incentivized experiment module recruiting representative adults to monitor policy-relevant sentiments. Falk et al. (2018) also use representative adult samples and report trust and other preferences in many more countries than those that we study. However, their approach draws on the methodology of experimental economics somewhat indirectly, by asking participants to make (non-incentivized) hypothetical decisions that were previously validated using a lab experiment conducted with a German student sample. Their trust measure relies on a survey question only. Trustab’s use of both incentivized and survey modules can be considered as a methodological alternative. Although Trustlab’s initial surveys included only a thousand participants per country, and were conducted in only a relatively small number of countries, all of them high-income democracies, the methodology has the potential be scaled up in both sample size and diversity of countries, so reporting its performance with respect to its core focus, making use of both experiment and survey modules in the analysis, should aid scholar and policymaker assessment of the value of this approach.

## 2 │ EXPERIMENTAL DESIGN AND DATA

### 2.1 │ Experiments

We analyse data from 7236 respondents in six samples representative of the general adult populations of Germany, Italy, Japan, Luxembourg, the UK and the USA. Data were collected by the Trustlab initiative (Murtin et al. 2018; Cetre et al. 2024) between 2017 and 2021, with the exact period varying by country.^1^ To our knowledge, Trustlab is the first initiative that collects experimental measures of trust, trustworthiness and other social preferences as well as extensive survey data among several nationally representative samples. The Trustlab data were collected through an online platform designed by the Organisation for Economic Co-operation and Development (OECD). Ethical approval was granted by the OECD Centre on Well-being, Inclusion, Sustainability and Equal Opportunity.

In each country, the online experiments and surveys were conducted with a sample of approximately 1000 respondents. These samples are nationally representative by age (e.g. 18–34 years, 35–49 years and 50–65 years), gender and income (e.g. bottom 20% of household income in each country, 20th–80th percentiles of household income, and top 20% of household income), and were drawn by a private sector polling company. The analysis includes 998 respondents from Germany and Italy, 2154 from Japan, 981 from Luxembourg, 1034 from the UK, and 1071 from the USA.^2^ Participants accessed the Trustlab platform online using a link provided by the polling company. Upon completion of the entire set of online experimental tasks and survey questions, participants received payment for one of the potentially incentivized games, with the game and role chosen randomly, and with the payoff implemented non-deceptively—that is, in the case of the trust game, by pairing participants and randomly assigning one to the first-mover role and one to the second-mover role.

All participants took part in a trust game, a (conditional) public goods game and a dictator game, as well as a multiple lotteries risk-taking task. Experiments were always conducted in this same order for all participants.^3^ Experimental instructions are shown in Online Appendix A. Before taking part in the experimental trust game (and also, subsequently, in the public goods game), participants took part in a simulation, where they could try out different choices.

Participants were told at the outset that only one of the ‘incentivized tasks’ would be chosen at random to be remunerated with real money. Participants could make up to 40 currency units (CU) in the trust game ($40 in the USA, £40 in the UK, 40 euros in Germany, Italy and Luxembourg, and 6000 yen in Japan), with lower maxima applying if the public goods game, dictator game or multiple lotteries task were chosen for payout.^4^ Participants earned 12 CU on average in Trustlab, with small cross-country differences.^5^ Only 3% of players earned 0 CU, and 5% earned 20 CU or more. Participants remained anonymous and were given no information on whom they were playing with. The one exception is that instructions clearly stated that in all the games, participants were matched with a person living in their country (e.g. in the trust game, a trustor from Italy was always matched with a trustee from Italy).

#### 2.1.1 │ Trust game

The trust game was played first. Both first mover (‘trustor’) and second mover (‘trustee’) received a 10 CU endowment. Trustors were able to transfer any part of their endowment to trustees. Any amount sent to trustees was tripled, and trustees could return any part of their funds to trustors. Participants first played the role of the trustor, and decided which amount to send. Using the ‘strategy method’ (Selten 1967; Mitzkewitz and Nagel 1993; Bardsley et al. 2009), participants then played the role of trustee and indicated for each of the eleven possible integer amounts sent (0–10 CU) how much they would return. The amount available to be returned by second movers was the tripled amount sent by the trustor plus their 10 CU baseline endowment.^6^ A considerable literature has interpreted the amount sent as a measure of trust (Berg et al. 1995; Johnson and Mislin 2011). Similar to Berg et al. (1995), Johnson and Mislin (2011), Sutter (2006) and Ashraf et al. (2006), we use as an indicator of trustworthiness the proportion of the amount available that is returned by trustees, that is, y/(3x+10), where *y* and *x* are the amounts returned and sent, respectively. We acknowledge, however, that first- and second-mover sending can also reflect other-regarding preferences (Cox 2004), so the terms trustor, trust, trustee and trustworthiness are adopted only as first approximations and will be further qualified as we proceed.

After completing the trust game, participants were asked how much money they expect to receive in return when sending 5 CU. Answers could range from 0 CU to 25 CU.^7^ In the analysis, we express expected returns in terms of standard deviation changes, to facilitate comparisons to other variables. Our measure of expected returns is somewhat different to the approach taken in some other studies. Ashraf et al. (2006), for instance, ask participants to indicate which proportion of the endowment they expect to receive back based on what they themselves had sent in the experiment. The approach used by Ashraf et al. (2006) creates a mechanical link between the amount sent and the amount expected in return, as the potential amount that players can receive in return increases with the amount sent. Because respondents in our study are asked about the same amount, we are able to circumvent this issue. However, unlike in Sapienza et al. (2013), participants were not asked how much they expected to receive in return for all the possible amounts sent, and their answer was not incentivized. This was done to reduce time demands, given the number of games and other components of the overall survey.

#### 2.1.2 │ Public goods game

The public goods game (Andreoni 1988; Isaac and Walker 1988) was played after the trust game. Participants were told that they were playing in groups of four individuals from the same country. Each participant received 10 CU, and could decide which proportion to contribute to a ‘joint project’. Any amount contributed was multiplied by 1.6, and the total amount of money invested would be split equally between the four participants.

Participants then took part in a conditional public goods game, which we use to measure conditional cooperation, following Fischbacher et al. (2001). Participants were asked how much money they would invest, based on the average contribution of the other three participants (i.e. subjects indicated how much they would invest when the average contribution of other players was 0, 1, …, 10 CU).^8^ For each participant, we regress the amount that they would contribute on the amount contributed by others, and use the slope coefficient as a measure of conditional cooperation, following Cetre et al. (2024). We use participants’ conditional cooperation also as a proxy for reciprocity, as the two measures are conceptually closely related. Reciprocity is the tendency to reward cooperative behaviour and punish uncooperative behaviour. Conditional cooperation captures the degree to which participants increase their own contribution to a public good, based on the contributions of others. Increasing your own contribution to a public good when others increase theirs is akin to rewarding cooperative behaviour. Decreasing your own contribution when others decrease theirs is akin to punishing uncooperative behaviour.

#### 2.1.3 │ Dictator game

The dictator game was played after the public goods game, using a standard experimental protocol (Camerer 2003). Participants were informed that they would be matched with a person from the same country but not someone with whom they had interacted in previous rounds. They were given a 10 CU endowment, told that the person with whom they were playing had received no endowment, and asked to decide whether they wanted to transfer any proportion of their endowment to the other participant, who had no decisions to make. The (standardized) amount sent in the dictator game is used to measure altruism or fairness preferences.^9^

#### 2.1.4 │ Multiple lotteries task

Risk preferences were also measured, as there is evidence for an association between trust and risk-taking (Eckel and Wilson 2004; Houser et al. 2010; Schechter 2007). In particular, the Binswanger–Eckel–Grossman multiple lotteries risk-taking task was played after the dictator game (Binswanger 1980; Eckel and Grossman 2002). Participants were told to choose one of six gambles, in each of which they could win one of two amounts. They were informed that the gamble was a random draw, comparable to a coin toss, and that each possible outcome had a 50% chance of occurring. The first gamble involved no risk as respondents would earn 8 CU regardless of the outcome. The level of risk increased to a 50/50 chance of earning 7 CU or 10 CU in the second gamble, 6 CU or 12 CU in the third, 5 CU or 14 CU in the fifth, and 1 CU or 19 CU in the most risky gamble. Participants could select any of the six gambles. We measure risk preferences as a semi-continuous variable, where higher values indicate a higher level of risk tolerance (i.e. participants choosing riskier gambles).^10^

### 2.2 │ Survey data

The Trustlab initiative also collected survey data on participants’ gender, age, household size, employment status, whether participants live in a rural area or village (as opposed to a town or a metropolitan area), and whether they were born in the country in which the survey was being administered. To measure life satisfaction, participants were asked to indicate how satisfied they were with ‘life as a whole’, on a scale from 0 to 10, where 10 refers to being completely satisfied (Dolan and Metcalfe 2012). Respondents also indicated how important religion was in their own life, on a scale from 0 to 10. To measure respondents’ preferences for redistribution, they indicated what they perceived to be a ‘fair split of the tax burden’ for different groups of earners (top 1%, next 9%, next 40%, and bottom 50%). They indicated the percentage of available resources that should be paid in tax by each group. We operationalize preferences for redistribution via four continuous variables, which capture the desired tax rate for each group.

Some personal characteristics were collected only in a subsample of countries. In all countries except Luxembourg, Trustlab captured some political views of respondents by asking them to score, on a scale from 0 to 10, how much they agree with the statements ‘There is not much opportunity to get ahead in society’, ‘People don’t have any say about what government does’ and ‘Our culture is undermined by immigrants’, which were respectively used to measure beliefs about social mobility and political efficacy, and anti-immigration attitudes.

Participants in Italy were asked about their marital status (single, married, divorced, widowed, legally separated). Respondents in Germany, Italy, Japan and the UK were asked about which religious group, if any, they belonged to (no religion, Catholic, Protestant, other Christian, Muslim, Jewish, Buddhist, other). Participants from Italy, Japan and the UK took part in a 15-item ‘Big Five’ personality test (see Online Appendix A for details).

Conformity was measured only in the UK. Participants were given five pairs of statements, and were asked which of each pair they agree with more, following Blais and Hortala-Vallve (2021). For example, they could choose between ‘It’s best for everyone if people try to fit in instead of acting in unusual ways’ (which is in line with conformist attitudes) and ‘People should be encouraged to express themselves in unique and possibly unusual ways’ (which is in line with non-conformist attitudes). The full list of statements is shown in Online Appendix A. We create a continuous measure of non-conformity, which indicates the proportion of items chosen that reflect non-conformity.

Finally, in all countries except Luxembourg, Trustlab elicited participants’ self-declared level of trust by asking them to what extent they agree with the statement ‘Generally speaking, would you say that most people can be trusted or that you cannot be too careful in dealing with people?’, on a scale from 0 to 10. This is a standard trust question used in the World Values Survey, the European Social Survey, and many other surveys (OECD 2017). Given the juxtaposition of incentivized and survey trust in our analysis, it is important to bear in mind that participants encountered the question among a great number of other survey questions, and that it was separated from the incentivized trust game task (instructions for which omit words such as trust or trustworthiness) by dozens of incentivized survey decisions.^11^

### 2.3 │ Data overview

Table 1 shows summary statistics on key characteristics of the participants and key decision variables, by country. Brief descriptions of the variables used in the analysis appear in Table A.1 of the Appendix below. Detailed experimental instructions appear in Online Appendix Section A.

### 2.4 │ Analysis

Our goal is to examine behavioural and survey response indicators of trusting and trustworthiness using a large and innovatively collected set of representative observations with broader coverage of potential correlates of choices in the trust game than in previous studies. While informed by theoretical approaches such as those of Hoffman et al. (1998) and Charness and Rabin (2002) on reciprocity, Cox (2004) on other-regarding preferences, and Eckel and Grossman (2002) on risk aversion, our contribution is primarily an empirical one, centred on estimating simple OLS regressions to examine the correlates of first- and second-mover sending decisions, as well as of survey responses. Our independent variables are Trustlab’s incentivized measures of expected second-mover returning, conditional cooperation, dictator game sending, and risk aversion, as well as a selection of the survey’s relevant demographic indicators and survey responses. For our three main dependent variables, we estimate the OLS model

(1) $$x_{i}=\beta_{1}R_{i}+\beta_{2}G_{i}+\beta_{3}A_{i}+\beta_{4}P_{i}+\gamma_{c(i)}+\varepsilon_{i},$$

where $x_{i}$ refers to the amount sent in the trust game by participant i, the response to the generalized trust survey question, or the proportion of available funds sent as second mover in the trust game, by participant i. Here, $R_{i}$ captures participants’ expected returns (standardized amount expected back when 5 CU are sent), $A_{i}$ captures their level of altruism or fairness concern (standardized amount sent in the dictator game), $G_{i}$ captures risk tolerance (higher values for riskier gambles), $P_{i}$ is a vector for participants’ personal characteristics (see Table 1), and $\gamma_{c(i)}$ captures country fixed effects. We add $R_{i}$, $A_{i}$, $G_{i}$ and $P_{i}$ successively. We focus first on correlates of first-mover sending in the trust game, then use the variants of the model to study correlates of the trust survey response and of amount sent by participants as trust game second movers. While the main analysis relies on OLS models, we also use alternative specifications (probit and ordered probit regressions), where the amount sent is dichotomized or divided into categories. We use the Shapley approach to capture the average marginal contribution of different explanatory variables to the overall R-squared across all permutations of the order in which variables are entered following Shorrocks (2013)—see also Henderson et al. (2018)—to gauge the proportion of behavioural variation explained by different factors.^12^ We also report simple bivariate correlations, for example to study the relationship between incentivized trust game sending and response to the survey trust question.

## 3 │ RESULTS

### 3.1 │ Trust and trustworthiness in Germany, Italy, Japan, Luxembourg, the UK and the USA

**FIGURE 1:**
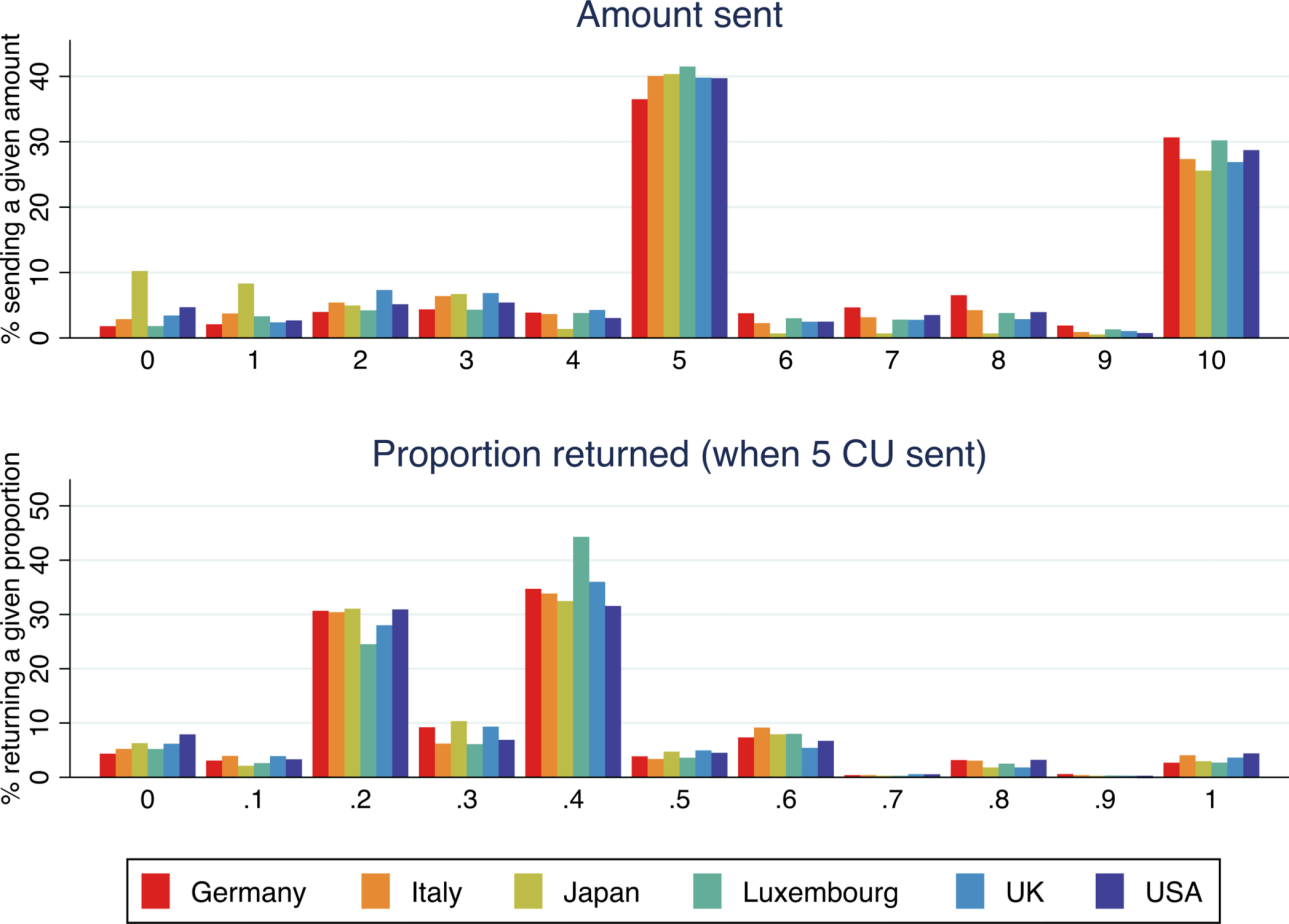
Amount sent and proportion of the available amount returned in the trust game (Germany, Italy, Japan, Luxembourg, the UK and the USA). *Notes*: The figure shows the distribution of choices for the amount sent and amount returned in the trust game for 998 respondents from Germany and Italy, 2154 from Japan, 981 from Luxembourg, 1034 from the UK, and 1071 from the USA. In the top panel, the bars show the proportion of trustors in each country that sent each amount between 0 and 10 CU to the trustee. In the bottom panel, the bars show the proportion of trustees in each country that returned between 0 and 100% of the amount available (i.e. 25 CU, three times the amount send (5 CU) plus the 10 CU endowment), where shares are rounded to the nearest decimal.

The top panel in Figure 1 provides an overview of the distribution of choices for the amount sent in the trust game in Germany, Italy, Japan, Luxembourg, the UK and the USA. On average, trustors sent 6 CU of their 10 CU endowment to trustees, with moderate cross-country differences.^13^ The distribution of the amount sent in the trust game is bimodal. Overall, 40% of trustors sent 5 CU, and 28% sent 10 CU. Only 5% of trustors sent 0 CU to the trustee. Japanese respondents are substantially more likely to send 1 CU or less, as 17% of respondents do so, compared to only 6% in the other countries. According to a meta-analysis of 162 trust games, the average amount sent is 50% of the endowment (52% in North America, and 54% in Europe) (Johnson and Mislin 2011). Amounts sent in our study are thus slightly above the average, but well within the range of variability that has been reported.

Trustees returned on average 34% of the amount available across the 11 scenarios that they were asked about (receiving 0 CU, 1 CU, and so on). The bottom panel in Figure 1 shows the distribution of choices for the amount returned in the trust game, when trustors send half of their endowment (5 CU).^14^ Trustees returned 37% of their endowment on average when asked about being sent 5 CU. The distribution of return rates is also bimodal on the two actions characterized by the sender’s break-even point (i.e. the sender is returned what she sent) and the equal split point (both players earn the same amount). There are modest differences in the proportion returned by country, statistically significant mainly for Japanese participants, who returned lower proportions on average.^15^ Second-mover behaviour is in line with previous studies. Based on a meta-analysis of 137 trust games, the average proportion of the available amount returned is 37% (34% in North America, and 37% in Europe) (Johnson and Mislin 2011). As one would expect, individual respondents’ choices as first mover and as second mover in the trust game were significantly correlated (Pearson correlation 0.32, *p* < 0.0001).

### 3.2 │ Why do we trust others?

#### 3.2.1 │ Expected returns

One common assumption is that first-mover behaviour in the trust game is to a large extent explained by whether the sender expects a sufficient amount to be returned so that it makes sending profitable to herself. On average, trustors expected to receive 9 CU in return when 5 CU are sent, which would make trusting profitable on average.^16^ But a non-negligible share expected to only break even, with some even anticipating a loss from sending. Table 2 groups the amount trustors sent in the trust game based on the amount they themselves expected to receive back.^17^ Both the amount sent and the expected return are divided into three categories. There is a clear link between how much trustors send in the trust game and how much they expect to receive back (*χ*^2^ *p*-value < 0.001). Trustors with low expectations of returns sent smaller amounts than those with high expectations, with the numbers in column (3) suggesting that fully 83% of those who reported expected return rates that made sending profitable sent half or more of their endowments. However, about 29% of sending decisions seem to be at odds with an intention to maximize own material payoff, and an additional 27% of participants sent CU with the apparent expectation of merely breaking even, a choice difficult to reconcile with the prevalence of at least modest risk aversion in most populations.^18^ Thus only about 44% of sending choices appear strictly explicable by self-interest, leaving considerable scope for other factors to be at play.

Model (1) in Table 3 examines the association between the amount sent in the trust game and the (standardized) expected return while controlling for country fixed effects in a simple OLS regression model based on equation (1) (see Subsection 2.4). Shapley values, which estimate the percentage of variance explained by each variable in the model displayed, are shown in the corresponding columns of Table 4 (in the present case, column (1)). We find that how much trustors expect to receive back from trustees is significantly associated with the amount sent (*p* < 0.01). A one standard deviation (SD) increase in the amount expected back increases the amount sent by 0.78 CU (or 0.27 SD). Hence self-interest appears to play a role in how much is sent in the trust game. The Shapley value for expected returns is 0.066—which can be interpreted to mean that expected returns can account for approximately 6.6% of the variance in the amount sent.^19^ This corresponds to 78% of the total explained variance in model (1) (the remaining 22% is associated with country fixed effects). We take this to suggest that while expected returns are relevant for trusting behaviour in the trust game, trusting is not only based on self-interest calculation.

#### 3.2.2 │ Trust: altruism, risk preferences, and personal characteristics

Having established that expected returns account for only a small part of the variation in participants’ experimental trusting behaviour, this subsubsection examines whether other *a priori* relevant factors are better at predicting behaviour. We look in particular at altruism or fairness preferences, at risk preferences, and at personal characteristics.

First, we examine the potential role of altruism or fairness concern. Cox (2004) and Carter and Castillo (2011) remind economists that participants in experimental games often share money with strangers in the absence of potential monetary gains, and that positive weight on the earnings of the other in one’s own utility (i.e. other-regarding preferences) can explain such choices. Although the Berg et al. (1995) trust game gives players equal initial endowments, which rules out sending out of fairness unless participants believe others’ out-of-game financial situations differ from their own, not all other-regarding preferences are strongly inequality averse. Moreover, the fact that the sent amount gets increased by a multiplicative factor has been found to increase sending in experimental settings. (See, for example, Charness and Rabin (2002), who posit an efficiency preference, that is, one for ‘enlarging the pie’ even at the expense of one’s own slice.) Although Trustlab did not include a variant of the dictator game in which sent amounts are tripled, a device that Cox (2004) uses to disentangle altruism from trust, we can use the behaviours of participants in the platform’s standard dictator game in the Table 3 partial correlation approach to partly distinguish altruistic or other-regarding motives from expectations of return in our data.

The amount sent in a dictator game is often interpreted as a measure of the weight placed on the payoff of the other relative to one’s own (Charness and Haruvy 2002; Engel 2011). Even though the amount sent in a standard dictator game can be sensitive to social norms (Krupka and Weber 2013) as well as the environment and how choices are framed (Dana et al. 2006; List 2007; Bardsley 2008), many researchers (e.g. Forsythe et al. 1994; Franzen and Pointner 2013) continue to interpret dictator game sending as an altruism indicator. Because the amount sent determines the division of 10 CU between the dictator and the participant chosen as recipient, and because their respective shares of those 10 CU will be the only experimental game earnings of two participants if the dictator game task is randomly selected as the basis of their earnings, we cannot rule out a fairness or inequality aversion interpretation of the behaviour in the dictator game (Bolton and Ockenfels 2000; Cappelen et al. 2007). Generalizing, we refer to a participant’s dictator game sending decision as a measure of their altruism or other-regarding preferences (ORP). In the dictator game, participants sent on average 4.3 CU of their 10 CU endowment to a stranger (including 9% who sent 0 CU, 51% who sent 5 CU, and 8% who sent 10 CU). As shown in a simple model that includes dictator game sending and country fixed effects only (model (2) in Table 3), altruism or ORP is significantly associated with first-mover sending in the trust game (*p* < 0.01). A 1 SD increase in altruism (ORP) is associated with an increase in the amount sent in the trust game by 1.1 CU (or 0.35 SD). As shown in column (2) of Table 4, altruism (ORP) alone can explain 12.5% of behavioural variation in the amount sent (88% of the total explained variance in model (2)).

Second, we examine the role of risk preferences. Participants took part in a multiple lotteries task, where 30% of participants choose the least risky gamble (8 CU with no risk), and 11% selected the most risky gamble (50/50 chance of winning 1 CU or 19 CU). As shown in model (3) of Table 3, participants who selected riskier gambles sent slightly larger amounts in the trust game. A one point increase in risk tolerance is associated with a 0.19 CU increase in the amount sent. These associations between trust and risk preferences make sense if trustors view trusting as a risky decision from a self-interested viewpoint. However, as shown in column (3) of Table 4, while the estimated coefficient on risk tolerance is significant at the 1% level, it appears to account for only 1.3% of the variation in trusting behaviour (38% of the total explained variance in model (3), the remainder being accounted for by country fixed effects).

Third, we examine the role of personal characteristics, ten of which are entered jointly along with the country fixed effects in model (4) of Table 3. Unlike previous studies that focus on small and homogeneous samples (for instance, university students or village neighbours), our sample is diverse and representative of people living in six countries. Although our data present the ideal setting for studying personal characteristics, we find little evidence that these can predict behaviour in the trust game. In the absence of the behavioural variables of the first three models, model (4) finds three of the ten factors to be significant at the 5% or 1% level: that women send 0.6 CU (0.18 SD) less than men in the trust game, that participants who indicate that they are completely satisfied with life send 0.3 CU (0.10 SD) more, and that those having a university education send an average of 0.18 CU (0.06 SD) more. Taken together, however, Table 4 shows that variation in all ten individual characteristics accounts for only 1.3% of the variance of first-mover sending, less than one-fifth as much as does expected return in model (1), and one-tenth as much as altruism (ORP) in model (2). Moreover, model (5) of Table 3, in which all variables are entered simultaneously, indicates that which individual characteristics are significantly correlated with first-mover sending depends on what controls are included, with life satisfaction losing significance, while having another diploma (beyond high school, but less than a bachelor’s or other university degree) obtains a positive and significant coefficient, and importance of religion a negative significant coefficient. Other plausible determinants of behaviour, such as age, place of residence or immigration status are not significantly associated with the amount sent in Table 3.^20^ As indicated in Table 4, personal characteristics account for only 36% of the total explained variance in model (4).^21^

Fourth, we examine the role of country fixed effects, which might capture cultural and institutional differences not captured by the other variables. In comparison to the reference category (Germany), players from other countries sent between 0.5 CU and 1 CU less in the trust game, a non-negligible difference since overall sending averages 6 of 10 CU. However, as shown in the bottom row of Table 4, country fixed effects account for only around 1–2% of the variance in the amount sent, so variation among individuals within countries far outstrips that between the average individual in different countries. Part of the reason for this might be that we include only data from OECD countries, which share important characteristics such as a high level of income. In addition, four countries are located in Europe. Japan is likely most dissimilar culturally to the other included countries, but differences in the amount sent are still modest. Nonetheless, it is likely that country fixed effects would be more important had a more diverse sample of countries been surveyed.

Finally, when we reintroduce expected returns, altruism and risk preferences along with personal characteristics and country fixed effects in model (5) of Table 3, positive coefficients on the three behavioural variables remain highly significant, although the point estimates of the coefficients on expected return and risk tolerance fall by about a third, and that on altruism (ORP) by a little over 13%. As shown in Table 4, altruism (ORP) explained the largest share of the variance in model (5) (10.4%), followed by expected returns (4.5%), country fixed effects (1.2%), individual characteristics (1.1%) and risk tolerance (0.9%). As altruism and expected returns also show the strongest degree of association with first-mover sending, we conduct an *F* -test to determine whether the coefficients of the two standardized variables are the same, and the result rejects the null hypothesis that the coefficients have the same magnitude (*F* < 0.0001). In view of the coefficient magnitudes and respective R-squared values of specifications (1) and (2), our results therefore suggest that altruism or fairness preference is the single most important predictor of the amount sent in our trust game, with expected return playing the next most important role. Although we include a large number of potential drivers of trust, 82% of behaviour in the trust game is not explained by the included factors.

#### 3.2.3 │ Trust: attitudes and values

As a large proportion of first-mover sending behaviour is not predicted by the factors explored in the previous subsubsection, we now examine other plausible correlates relating to participants’ attitudes and values, specifically preferences for redistribution, conformity, religion, personality traits, negative personal events (i.e. divorce) and political views.^22^

First, it is plausible that trusting behaviour is linked to participants’ preferences for redistribution. Participants who prefer a more equal distribution of resources within society might also be willing to risk some loss of endowment to help an anonymous counterpart.^23^ As shown in Online Appendix Table A, we find that—when expected returns, altruism and risk preferences are controlled for—participants who desire a higher tax burden for the top 1% and top 9% of earners send more in the trust game, and those who desire a higher tax burden for the bottom 50% send less. Redistributive preferences explain no more than 0.03% of the variance in the amount sent.^24^

Second, beliefs about community norms and inclinations to adhere to such norms, which might vary from participant to participant, could account for some differences in amounts sent as first mover. As mentioned in Subsection 2.2, data on conformity were collected in the UK Trustlab following the approach of Blais and Hortala-Vallve (2021). As shown in Online Appendix Table A, individuals who feel less pressure to conform to community norms send 0.6 CU (0.20 SD) more in the trust game when expected returns, altruism and risk preferences are controlled for. Based on Shapley values, non-conformity explains only 0.2% of behavioural variation in trust (based on model (2) of Table A).^25^

Third, using data from Germany, Italy, Japan and the UK, and controlling for country fixed effects, we test whether religious affiliation is significantly correlated with trust. As shown in Online Appendix Table A, Catholics appear to send 0.27 CU less, while those who self-categorize as ‘not religious’ send 0.25 CU more in the trust game when expected returns, altruism and risk preferences are controlled for.^26^ Religion explains no more than 0.08% of variation in the amount sent.^27^

Fourth, we examine the role of marital status and political views. Using data from Italy, we examine whether married or divorced participants differ in their trusting behaviour—which has been found when trust is measured by survey questions (Alesina and La Ferrara 2002).^28^ As shown in Online Appendix Table A, we find no evidence to suggest that this is the case. We also examine whether political views—specifically attitudes towards immigration, participants’ senses of their own political influence or power and the perceived degree of social mobility in society—are correlated with the amount sent in the trust game. We find no evidence suggesting this (see Online Appendix Table A).

Finally, as one might argue that trust is similar to a personality trait (Freitag and Bauer 2016), we also analyse the role of ‘Big Five’ personality traits in Italy, Japan and the UK. As shown in Online Appendix Table A, a one point increase in neuroticism lowers the amount sent by 0.06 CU, while an increase in extraversion or in openness increases the amount sent by 0.05 CU. These results are significant at the 1%, 10% and 5% levels, respectively, and are intuitively consistent with conventional understandings of the traits in question. However, as highlighted in Online Appendix Table A, the relationships lose significance and in one case reverse sign once expected returns, altruism and risk preferences are controlled for, perhaps because the experimental behaviours jointly capture most information in the personality measures. Personality traits explain no more than 0.3% of variation in the amount sent.^29^

#### 3.2.4 │ Self-reported trust

The preceding subsubsections have used the trust game as a controlled setting to capture trusting behaviour directly. A virtue of the Trustlab methodology is that we can also now examine whether self-reported trust correlates with first-mover sending, and study whether trust is correlated with the same factors as the incentivized decision. Answers to the self-reported trust question—that is, participants’ responses to the generalized trust question, ranging from option 0, ‘You can’t be too careful’, to 10, ‘Most people can be trusted’—are available from participants in all but one country studied. As shown in Online Appendix A, average self-reported trust is highest in Germany and the USA (respondents score 6 and 5.9, on average), followed by the UK (5.5 on average) and Italy (5 on average). Average self-reported trust is lowest in Japan (4.3 on average).^30^ The bivariate correlation between first-mover sending observed in the trust game and response to the survey trust question is positive and significant on average, although modest in magnitude (*r* = 0.13, *p* < 0.0001). As shown in Online Appendix Figure A, the correlation between the amount sent in the trust game and self-reported trust is significant at the 1% level in each of the countries except Italy, where its value is 0.06 and it is significant at the 10% level only. In view of the studies discussed above, some of which fail to find the incentivized game and the survey trust measure to be correlated, and given the considerable separation between the two components in the Trustlab platform, this finding in a large and representative sample is noteworthy. We take this to suggest that while there are differences between the game and the survey, they appear to capture similar underlying constructs (see also Banerjee et al. 2021).

Table 5 reproduces the regression analysis of Table 3 but replaces first-mover sending with self-reported trust as an outcome. Despite the differences between the two measures, the estimated coefficients are qualitatively similar, although a larger number of the personal variables obtain statistically significant coefficients. The high levels of significance of the coefficients on the three task-based variables (expected return, altruism (ORP) and risk tolerance), the relative importance of the first two compared to the third, the high significance of the female and country fixed effect coefficients, and their similar relative magnitudes in Tables 3 and 5, are arguably among the strongest evidence in the literature to date suggesting that incentivized trust game sending and responses to the general survey trust question are picking up closely related if not identical dispositions. As was the case with trusting behaviour observed in the trust game, only a small proportion of variation in self-reported trust is explained by the included factors (15%, compared to 18% in the trust game).

### 3.3 │ What determines trustees’ choices?

This subsection examines the correlates of second-mover sending in the trust game. We use a regression model resembling equation (1) in Subsection 2.4, except that we add conditional cooperation in the public goods game as a regressor, because reciprocity is likely a key motive for second movers to return some of the money sent them by the matched first mover. Rather than control for the first mover’s sending as an independent variable, we put tripled first-mover sending plus the second mover’s endowment in the denominator of the dependent variable, second-mover sending per available CU.^31^ Our OLS estimating model is thus

(2) $$\frac{y_{ix}}{3x+10}=\beta_{1}\;CC_{ix}+\beta_{2}G_{i}+\beta_{3}A_{i}+\beta_{4}P_{i}+\gamma_{c(i)}+\varepsilon_{ix},$$

where $y_{ix}/(3x+10)$ captures the proportion of the available amount sent (back) by second mover *i* under the condition that the matched first mover sent amount x. Trustees were asked how much they would ‘send back’ to trustors for each possible integer amount sent (0 CU to 10 CU). The variable $CC_{ix}$ captures participants’ conditional cooperation (i.e. the best-fitting slope of their contribution conditional on each possible average contribution of others in the conditional public goods task, our proxy for reciprocity). All other parameters are defined as in equation (1).

We begin with a descriptive look at $CC_{ix}$, based on the Trustlab conditional cooperation task. Participants with negative scores on this measure (3%) decrease their contribution to a joint project when others increase theirs. Participants who score zero (15%) do not change their own contribution in response to other people’s.^32^ Most participants (82%) have positive scores, increasing their contribution to a public good when others increase theirs. As a verification exercise, we consider the Finan and Schechter (2012) approach of using the trust game itself to generate a binary measure of reciprocity that indicates whether trustees return a higher share of the available money when large amounts (≥ 5 CU) are sent by trustors compared to when small amounts (< 5 CU) are sent.^33^ We find that our measure of conditional cooperation based on our conditional public goods game data is highly correlated with this measure. Participants who return a higher fraction of the available money when they receive larger amounts also score 0.77 SD higher on our measure of conditional cooperation. This supports the idea that conditional cooperation in the conditional public goods game is a valid measure of reciprocity.^34^

Also, before estimating equation (1), it is important to mention preliminary analysis which reveals that conditional cooperation has a significant impact on second-mover sending, but one that is qualitatively different for different amounts received. Specifically, panel A of Online Appendix Table F.1, reports a separate regression of the proportion returned on conditional cooperation, for each of the eleven possible first-mover sending amounts. The estimates show that conditional cooperation is a statistically significant predictor of the proportion returned for most amounts sent, but that it has significant negative coefficients for sent amounts between 0 and 3, and significant positive coefficients for sent amounts between 6 and 10. Point estimates of the coefficients increase monotonically with the amount sent. This pattern would be difficult to explain if the Trustlab trust game followed Berg et al. (1995) in every detail, but it is instead a reassuring and illuminating by-product of Trustlab’s slightly different design, which allowed second-movers to send money to their first-mover counterparts not only from the tripled funds received, but also from their endowments. The design thereby allows a second mover who was sent nothing to still send up to 10 CU to their counterpart, if so inclined. What the results in Table F.1 demonstrate is that second movers with strongly reciprocal preferences, reflected in high values of $CC_{ix}$, tended to send to their first mover only if entrusted with funds by the latter’s sending decision. There is a positive association between second-mover sending and altruism (dictator game sending) even at 0 and low amounts received (see panel B of Table F.1), because second movers having weaker reciprocal preferences and strongly positive other-regarding preferences sent money to their first mover even when the latter had sent little or nothing. The more reciprocal a second mover was (as shown by $CC_{ix}$), the less they sent to a first mover who showed little or no generosity or trust towards them—precisely as expected from understandings of reciprocity in the economics and other social science literatures. Panel B also shows that altruism’s positive impact becomes smaller when the first mover had sent a relatively large amount.

To avoid the clutter of presenting a separate estimate for each first-mover sending amount, and to leave room for more than one specification, Table 6 presents our estimates^35^ of both the full model of equation (1) and two partial versions, while grouping the data into three sending ranges: 0–3 CU (shown in columns (1)–(3)), 4–6 CU (columns (4)–(6)) and 7–10 CU (columns (7)–(9)). The first estimate covering each range includes only $CC_{ix}$ and country fixed effects as explanatory variables; the second adds controls for individual characteristics; and the third adds and displays the estimates for the altruism and risk proclivity variables. Consistent with the results of Online Appendix Table F.1, $CC_{ix}$ picks up negative and significant coefficients for the 0–3 CU first-mover sending range, positive and significant coefficients for the 7–10 CU sending range, and small positive coefficients for the 4–6 CU sending range, the coefficient on $CC_{ix}$ in the latter range being insignificant until it is joined by the altruism and risk proclivity controls (column (6)). Within each range, the point estimates of the coefficient on $CC_{ix}$ are relatively insensitive to specification, but across the three estimation sets, they display the aforementioned pattern of monotonic increase. In estimates (3), (6) and (9), altruism (dictator game sending) also obtains a highly significant positive coefficient, mildly declining from 0.078 for the 0–3 CU range (column (3)) to 0.060 for the 7–10 CU range (column (9)), similar to its decline when the sole behavioural determinant in Table F.1. Together, the estimates for the two key coefficients indicate that both reciprocity and altruism are important to second-mover sending, the latter finding resonating well with the arguments of Cox (2004) and Carter and Castillo (2011). Put succinctly, participants acting in the second-mover role, all of whom have resources to share with their first mover even when sent little or nothing, send less (more) to the first mover the stronger is their tendency towards reciprocity when the first mover exhibits less (more) trust and generosity. Relatively other-regarding second movers send money to the first mover in all circumstances, with the role of reciprocity growing and that of other-regarding preference declining as first-mover sending increases. Risk attitudes also obtain positive and significant coefficients, although second-mover sending is not a risky decision.^36^

As was the case with first-mover sending, our main analysis of the correlates of second-mover sending in the tables above has not made full use of all potentially relevant elements in the extensive Trustlab survey, in part because some information was collected in a single country or a few countries only. We therefore also investigated other factors that might be correlated with second-mover behaviour, in particular: preferences for redistribution (Online Appendix F.3), conformity (F.4), religion (F.5), personality traits (F.6), negative personal events (F.7) and political views (F.8). Tables in Online Appendix F.3 suggest that those who prefer a more progressive tax scheme (hence more redistribution) tend to send a larger proportion as second movers when 0 CU or 1 CU are received. Results also suggest that Catholics return larger amounts when 0 CU are sent, and that participants with no religion return less when under 5 CU are sent (see Online Appendix F.5). As indicated in Online Appendix F.6, extraversion is positively correlated with the proportion returned when less than 5 CU are sent. We also find that married individuals return less overall (see Online Appendix F.7), as do participants with attitudes that are not supportive of immigration (see Online Appendix F.8). However, while there are significant correlations between these factors and second-mover behaviour, these are generally small in magnitude, and none of the factors predicts a meaningful amount of behaviour variation— as the proportion of the variance explained is generally less than 1%.

### 3.4 │ Robustness

As shown in Online Appendix G, our results are robust to a number of alternative specifications. We measure trusting behaviour in the trust game in different ways. First, Tables G1 and G2 re-run the main analysis using a binary measure of the amount sent, which captures whether participants sent a non-zero amount, using OLS and probit specifications. Second, Tables G3 and G4 capture whether respondents sent more than half of their endowment, using OLS and probit specifications. Third, Table G5 categorizes the amount sent (less than half, half, more than half). We find that results remain qualitatively unchanged when binary or categorical measures of the amount sent are used, as well as when probit regressions are used instead of OLS.

We also examine how the larger sample from Japan influenced the results. We have over 2000 participants from Japan, compared to roughly 1000 for the other countries. As shown in Online Appendix Table G6, we reproduce the main analysis using a random subsample of 1000 Japanese respondents. This sample does not differ from the full sample based on observable characteristics (see Table G7). As shown in Table G8, we also re-run the analysis using weights to account for differences in sample size.^37^ Both of these approaches produce qualitatively very similar results, suggesting that the larger Japanese sample does not affect our results.

Finally, Online Appendix Tables G9 - G38 reproduce the main analysis separately for each of the six countries. Overall, the results are qualitatively similar for the different country samples. One exception is that in the UK, expected returns explain a slightly larger share of the variation in the amount sent than altruism ORP (8% as compared to 6%)—which is not the case for any of the other countries (see Table G12). Overall, the determinants of trusting and trustworthy behaviour appear generally similar across the included countries.

## 4 │ DISCUSSION

We study first-mover sending to strangers in an incentivized online trust game as a controlled setting to investigate, with the aid of accompanying game decisions and survey responses, the potential underlying motives for a decision widely used to proxy trust. We use data from six samples representative of the general adult populations of Germany, Italy, Japan, Luxembourg, the UK and the USA, with a total of 7236 participants. We find that trustors on average send 60% of their endowment to trustees, and that trustees return 34% of the amount available—an amount that makes trusting pay off on average, with only small cross-country differences. We find that self-interest (expected return by the receiver) and risk aversion are significant predictors of the amount sent by the first mover. However, our results suggest that the single most important correlate of first-mover sending in the trust game is giving in the dictator game, which is an indicator for other-regarding, altruism or fairness preferences, and explains 13% of behavioural variation. This finding adds considerable support for the caveat (Cox 2004) that first-mover sending can reflect both other-regarding preferences and trust in the proper sense of willingness to rely on the trustworthiness of the counterpart. Our findings validate the use of first-mover sending as a trust proxy not only because of its robustly significant correlation with self-reported expected return, but also because we provide one of the largest and most representative sets of observations of both first-mover sending in the trust game and responses to the generalized trust survey question, and we find the two to be significantly correlated (at the 1% level in large samples of four countries, at the 10% level in a fifth). We also analyse the correlates of second-mover sending in the trust game, and find that while second-mover behaviour is significantly positively correlated with reciprocity (proxied by conditional cooperation) when large amounts are received, altruism or fairness preference explains the largest share of the variance in second-mover behaviour (an average of 10%, with variation across received amount levels). While the importance of other-regarding preferences for second-mover sending in the Trustlab data may be higher due to the atypical design choice of allowing endowment too to be sent in that role, that feature generates a novel and illuminating added finding: that stronger reciprocal preferences actually discourage second-mover transfers when the first-mover sends little or nothing, as revealed by highly significant coefficients on the reciprocity measure being negative predictors of sending when the ‘trustee’ is sent less than a third of the first mover’s endowment.

In this study, we do not take as given the assumption that trust is determined only by calculative motives. Instead, we leave open what the determinants of trust might be, and we examine the potential role of a large range of factors that are *a priori* relevant. We find that risk preferences, participants’ gender and religion, as well as their preferences for redistribution and conformity, are correlated with trusting behaviour. However, the share of behavioural variation explained is small. Our main finding is that altruism is the single most important predictor of first-mover sending. Parallel regressions replacing trust game sending with the survey generalized trust response likewise find both expected return in the trust game and altruism to be significant correlates of trust, with altruism again being quantitatively the more important of the two, if by only a small margin (*F* < 0.0001). One way to interpret the finding that trust game sending is associated with giving in the dictator game is that players are influenced by the emotional valence with which they view the possibility that the trustee will return somewhat less money than the trustor would have hoped, and will keep more of it for themselves. Players with a generous attitude might reason that trustees who keep the money really need it, and such individuals can therefore feel good about having been generous. Players with such generous feelings about making the anonymous other better off in the trust game are likely also more inclined to give more in the dictator game. In contrast, players who have more negative emotions about trustees keeping the money they sent, such as feelings of being taken advantage of, are also unlikely to give in the dictator game.

We also find that the majority of behavioural variation (approximately 85%) in trust game sending is unexplained, although we include a large set of plausible determinants as explanatory variables. Interestingly, this is not only a feature of the trust game, but is also true when we use the most common survey measure of trust. This raises the question of what accounts for the remainder of behavioural variation. A growing body of research emphasizes the relevance of non-economic factors in accounting for differences in prosociality. The climate could be one such factor. Buggle and Durante (2021) find that European regions with higher pre-industrial climatic variability display higher levels of trust today, suggesting that climatic risk in the past favoured inter-community exchange and emergence of trust-engendering political institutions. Exposure to pathogens may be another factor. It has been posited that exposure to diseases may have the effect of binding individuals in a society more closely to one another (Thornhill and Fincher 2014; Zaleskiewicz et al. 2015). This seems to have been the case during the COVID-19 pandemic, as individuals more exposed to the pandemic seem to have increased their propensity to help others (Grimalda et al. 2021), albeit perhaps only temporarily (Gambetta and Morisi 2022). The type of agricultural production in which people were involved since the ancient past, such as growing wheat or rice, also seems to have long-lasting effects on propensities to cooperate with one another (Talhelm and Dong 2024; Talhelm et al. 2014). Also, environmental clues—for example, the atmosphere created by images in a virtual lab—have been found to influence trusting behaviour (Atlas and Putterman 2011). Furthermore, trust may have historical roots. It has been suggested that higher rates of capture and embarkation in the slave trade, perhaps by shaping optimal strategies of watchfulness and distrust, may have been passed down and came to persist over time in Africa (Nunn and Wantchekon 2011). This is in line with other work in economics that finds that attitudes about trust and other social preferences are transmitted from parents to children (Algan and Cahuc 2010; Dohmen and Falk 2011). Finally, genetic variability and hormonal functions may also affect prosociality (Cesarini et al. 2008; Zak et al. 2005). These environmental and historical factors plausibly account for some of the variation in trusting behaviour that is left unexplained in our analysis.

To our knowledge, our study is the first to systematically investigate such a broad range of correlates of trusting behaviour in large samples representative of the general population in several countries. We are able to reproduce the results of previous small-scale studies that find evidence for the importance of altruism in explaining trusting behaviour, for example between fellow students (Ashraf et al. 2006; Sapienza et al. 2013). Our findings suggest that altruism is important for trust between strangers within sizeable countries, rather than solely among those who share the same local situation.

In addition, previous small-scale studies also find that commonly examined factors explain only a small share of behavioural variation in trust. Sapienza et al. (2013), for example, find that 85% of trusting behaviour between students is unexplained. We show that this finding holds also in our large and representative sample. Notably, this finding is closely in line with the studies that measure trust with survey questions, rather than experimental games: using a large sample from 76 countries, Falk et al. (2018) find that 83% of variation in survey responses on trust is unexplained by the factors that they examine.

Our study is limited in four main respects. The first relates to our use of the ‘strategy method’ (Selten 1967; Mitzkewitz and Nagel 1993; Bardsley et al. 2009) to capture trustworthiness (i.e. second-mover returning behaviour). While the use of the strategy method is common in experimental economics, there is mixed evidence on whether it provides the same type of responses as when choices are observed in direct interactions (Brandts and Schram 2001; Charness and Rabin 2002; Brosig et al. 2003; Oxoby and McLeish 2004; Casari and Cason 2009; Brandts and Charness 2011). Fortunately, findings about first-mover sending and its relation to survey trust, which are most central to Trustlab’s mission, have no chance of being affected by this feature of the second-mover decision, since each participant made a first-mover decision before learning how their second-mover choice would be elicited.

The second limitation has to do with the way expected returns are captured in the Trust-lab data. Respondents are asked how much they expect that their counterpart would return in a situation where they sent 5 CU. They were not asked about other amounts, and their belief responses were not incentivized, as was done for instance in Sapienza et al. (2013). As sending 5 CU was the most common choice in the trust game, our sense is that this scenario was cognitively easy for participants to imagine. Nonetheless, we are unable to observe how expected returns differ among the whole spectrum of participants’ choices. It is, however, reassuring that Sapienza et al. (2013), who ask participants to indicate how much they expect to receive in return for all possible amounts sent, find that the correlation between expected returns and the amount sent in the trust game is very similar across scenarios—except when participants are asked about sending very small amounts.

The third limitation relates to the fact that with a 10 euros endowment for both participants, and a modest chance that the game will be the one randomly chosen to be paid off on, the incentives used in the trust game were modest. If behaviour relating to trust and altruism indeed depend on the monetary stakes, this puts the generalizability of our results into question. For instance, would altruism still be more relevant than expected returns if the stakes were higher, say with 1000 euros or more at stake? The literature on how stakes influence behaviour in economic experiments shows mixed results, as some studies find that stakes do not impact behaviour meaningfully (Cameron 1999), while others find that participants become less trusting (Johansson-Stenman et al. 2009), more risk averse (Binswanger 1980; Holt and Laury 2002) and more tolerant of unfairness (Andersen et al. 2011) when monetary stakes increase. Finally, experimental games were not played in a random order—which was not possible due to the online platform used by Trustlab in different countries. We therefore cannot exclude that order or spillover effects could have affected decisions. As the trust game was always played first, however, any such effects apply only to our explanatory variables.

Notwithstanding these limitations, there are at least two broad takeaways from our findings.

First, first-mover sending in the trust game—and perhaps trust itself in the sense of willingness to incur vulnerability on the basis of positive expectations of the behaviour of a trustee—are likely based on a broader range of factors than is commonly assumed. The trust game is arguably the simplest setup in which to observe trusting decisions. The potential payoffs and losses are clearly defined, and decisions are made in relation to windfall money. In addition, interactions are one-off, and the participants are anonymous to one another. The absence of reputational concerns, as well as the unusual setting in which decisions are made, likely leaves more room for self-interested motives than real-world decisions about trust. It is striking, then, that even though the playing field in which first-mover sending decisions are made in the trust game is so well defined, the most common prediction of what determines sending (i.e. expected returns) explains such a small share of behavioural variation. Our findings suggest that even in this highly stylized and simple setting, sending is not mostly explained by pecuniary self-interest.

Second, our sense is that, given the complex set of factors that determine trusting behaviour, and given the considerable interest in measuring trust, it may be important to adopt a broader and more inclusive understanding of trust in economics. Very commonly used definitions, such as those by Gambetta (1988)—who focuses on the probability that others will perform actions that are beneficial to us—do not appropriately capture the generous and prosocial disposition that can also underlie some trusting decisions, including first-mover sending in experiments based on Berg et al. (1995). Moreover, players in our experiment acted as trustors and trustees, and are representative of the population in six countries. In a one-shot trust game, trustworthiness cannot possibly be due to self-interest. It makes little sense to assume that social motivation comes into play only when participants act as trustees, but that their actions as trustors are strictly rational and self-interested. Whichever share of trustees depart from their strict rational self-interest, a similar share of trustors presumably do so as well.

Our findings are in line with the definition of altruistic trust proposed by Mansbridge (2010). According to this conceptualization of trust, we at times trust others more than is warranted by the available information, and do so because we enjoy giving them ‘the benefit of the doubt’. This definition leaves room for trust being a way to express respect and to treat others as we would wish to be treated ourselves. Hence, next to mere self-interest, it may be that we can also engage in trusting actions to uphold an ideal that benefits others, to confirm to ourselves our sense of belonging to society, and to experience the consequential warm glow (Andreoni 1990). Despite the steady stream of research on the topic during the past four decades, much that could prove useful to building or restoring social trust and well-functioning societies continues to be incompletely understood and deserving of further research.

## Supplementary Material

Online Appendices

## Figures and Tables

**TABLE 1:** Descriptive statistics (by country).

	(1)		(2)		(3)		(4)		(5)		(6)	
	Germany		Italy		Japan		Luxembourg		UK		USA	
	Mean	SD	Mean	SD	Mean	SD	Mean	SD	Mean	SD	Mean	SD
*Player characteristics*												
Female	0.50	0.50	0.51	0.50	0.47	0.50	0.48	0.50	0.54	0.50	0.51	0.50
High school or less	0.28	0.45	0.51	0.50	0.30	0.46	0.29	0.45	0.50	0.50	0.20	0.40
Non-tertiary diploma	0.38	0.49	0.17	0.38	0.12	0.33	0.20	0.40	0.15	0.36	0.38	0.48
University	0.34	0.47	0.32	0.47	0.58	0.49	0.51	0.50	0.35	0.48	0.42	0.49
Age	43.98	13.16	42.27	12.22	42.75	13.18	46.09	15.48	41.27	13.35	45.41	13.70
Respondent born in country	0.94	0.24	0.96	0.19	1.00	0.04	0.63	0.48	0.89	0.31	0.91	0.29
Household size	2.49	1.23	3.06	1.20	2.84	1.30	2.94	1.34	2.57	1.24	2.50	1.29
Rural	0.43	0.50	0.51	0.50	0.04	0.20	0.89	0.32	0.25	0.43	0.23	0.42
Working	0.70	0.46	0.67	0.47	0.65	0.48	0.69	0.46	0.65	0.48	0.63	0.48
Religion important	0.30	0.46	0.50	0.50	0.21	0.40	0.19	0.40	0.26	0.44	0.59	0.49
Completely satisfied with life	0.13	0.34	0.15	0.35	0.06	0.24	0.12	0.32	0.15	0.36	0.17	0.38
Social mobility is low	0.27	0.45	0.32	0.47	0.44	0.50	0.33	0.47	0.35	0.48	0.27	0.45
Tax burden top 1%	43.93	15.91	40.23	17.85	42.97	17.51	43.71	17.98	43.63	16.16	39.82	17.46
Tax burden next 9%	33.99	10.74	32.55	11.60	34.14	11.39	33.76	11.26	33.45	11.04	31.42	11.62
Tax burden next 40%	23.28	4.84	23.12	5.27	22.71	4.67	22.67	5.14	23.25	4.72	23.62	5.03
Tax burden bottom 50%	14.24	10.10	17.11	13.27	15.77	11.23	15.67	12.97	14.93	10.90	17.10	12.13
Low political efficacy	0.27	0.44	0.13	0.34	0.59	0.49	—	—	0.21	0.41	0.27	0.45
Against immigration	0.32	0.47	0.39	0.49	0.40	0.49	—	—	0.30	0.46	0.19	0.39
*Key variables*												
Amount sent in TG (CU)	6.51	2.79	6.01	2.92	5.26	3.29	6.31	2.84	5.92	2.95	6.04	3.01
Return expected in TG (CU)	8.98	4.47	9.91	5.52	7.37	4.86	9.54	4.78	8.82	5.01	9.18	5.46
Amount returned in TG (CU)	9.30	4.87	9.58	5.35	8.87	4.81	9.67	4.72	9.13	5.00	9.19	5.55
Earnings (CU)	11.97	6.88	12.80	4.58	10.36	5.77	11.96	7.07	12.09	13.17	11.83	6.76
Amount sent in DG (CU)	4.64	2.24	4.33	2.28	3.92	2.64	4.65	2.23	4.29	2.38	4.70	2.56
Risk tolerance	2.91	1.69	2.84	1.59	2.71	1.72	2.95	1.71	2.81	1.61	3.01	1.77
Survey trust	5.98	2.00	5.02	2.27	4.29	2.56	—	—	5.49	2.32	5.85	2.44
Observations	998		998		2154		981		1034		1071	

*Notes:* Data are from six representative samples of respondents in Germany (July 2017 to July 2018), Italy (June–July 2018), Japan (January–February 2020), Luxembourg (May–June 2021), the UK (June–July 2018) and the USA (June–July 2017). CU stands for currency units, TG stands for trust game, and DG stands for dictator game. Amount returned in TG is the amount chosen conditional on the first mover sending 5 CU and the participant as second mover having a maximum of 25 CU from which to ‘return’. Earnings capture the total amount paid out to players for taking part in Trustlab.

**TABLE 2:** Amount sent and amount expected in return in the trust game.

	Expects < 5 CU	Expects 5 CU	Expects > 5 CU	All
	(1)	(2)	(3)	(4)
Sent less than half (< 5 CU)	45.13	30.43	16.96	23.33
Sent half (5 CU)	29.08	45.31	39.21	39.79
Sent more than half (> 5 CU)	25.79	24.26	43.83	36.89
All	100.00	100.00	100.00	100.00

*Notes:* The table shows the amount that participants sent in the trust game and the amount that they expect to receive back. Each column shows the breakdown of those with the expectation in its heading into the three decision categories. Data are from six representative samples (7236 respondents) in Germany, Italy, Japan, Luxembourg, the UK and the USA.

**TABLE 3:** Potential determinants of trusting behaviour (OLS regressions).

	Amount sent in the trust game				
	(1)	(2)	(3)	(4)	(5)
Expected return (standardized)	0.781***				0.511***
	(0.036)				(0.035)
Altruism ORP (standardized)		1.077***			0.932***
		(0.034)			(0.035)
Risk tolerance			0.195***		0.135***
			(0.021)		(0.019)
Female				−0.557***	−0.428***
				(0.072)	(0.067)
Other diploma				0.160	0.216**
				(0.101)	(0.093)
University				0.180**	0.307***
				(0.085)	(0.078)
Age				0.001	−0.001
				(0.003)	(0.002)
Born in country				−0.016	0.037
				(0.134)	(0.124)
Household size				−0.005	−0.032
				(0.029)	(0.026)
Rural				0.026	0.018
				(0.093)	(0.086)
Working				0.124	−0.011
				(0.077)	(0.071)
Religion important				−0.060	−0.234***
				(0.081)	(0.074)
Completely satisfied with life				0.278**	0.024
				(0.111)	(0.102)
UK	−0.567***	−0.439***	−0.572***	−0.528***	−0.376***
	(0.130)	(0.126)	(0.133)	(0.137)	(0.126)
Italy	−0.648***	−0.372***	−0.494***	−0.452***	−0.359***
	(0.131)	(0.127)	(0.135)	(0.138)	(0.128)
Japan	−1.008***	−0.937***	−1.213***	−1.236***	−0.829***
	(0.113)	(0.109)	(0.115)	(0.125)	(0.115)
Luxembourg	−0.284**	−0.203	−0.208	−0.234	−0.293**
	(0.132)	(0.127)	(0.135)	(0.150)	(0.138)
USA	−0.499***	−0.495***	−0.490***	−0.460***	−0.474***
	(0.129)	(0.125)	(0.132)	(0.136)	(0.125)
Observations	7236	7236	7236	7236	7236
R-squared	0.083	0.141	0.033	0.033	0.182

*Notes:* Results are from OLS regression models including country fixed effects and a constant (not shown). The dependent variable in all models is the amount of money sent by the trustor in the trust game (mean 5.8 CU, SD 3.1). For country fixed effects, Germany is the reference category. Data are from six representative samples of respondents in Germany, Italy, Japan, Luxembourg, the UK and the USA. ORP stands for other-regarding preferences.

***, **, * indicate *p* < 0.01, *p* < 0.05, *p* < 0.1, respectively.

**TABLE 4:** Potential determinants of trusting behaviour (Shapley values).

	Shapley values				
	(1)	(2)	(3)	(4)	(5)
Expected return (standardized)	0.066				0.045
Altruism ORP (standardized)		0.125			0.104
Risk tolerance			0.013		0.009
Individual characteristics				0.013	0.011
Country fixed effects	0.017	0.016	0.021	0.021	0.012

*Notes:* The table shows Shapley values (estimated share of variance explained) for potential determinants of trust. Each column corresponds to a model in Table 3, and cells show Shapley values for key variables of interest. For instance, column (1) shows Shapley values for expected returns and country fixed effects (i.e. the variables included in model (1) of Table 3). Grouped Shapley values are shown for individual characteristics and country fixed effects. Expected return and altruism are standardized by subtracting their means and dividing by their standard deviations. By construction, Shapley values of a regression’s independent variables sum to its R-squared.

**TABLE 5:** Potential determinants of response to the survey question on trust (OLS regressions).

	Self-reported trust				
	(1)	(2)	(3)	(4)	(5)
Expected return (standardized)	0.319***				0.192***
	(0.031)				(0.031)
Altruism ORP (standardized)		0.340***			0.250***
		(0.030)			(0.031)
Risk tolerance			0.060***		0.029*
			(0.018)		(0.017)
Female				−0.199***	−0.165***
				(0.060)	(0.059)
Other diploma				0.047	0.065
				(0.084)	(0.083)
University				0.405***	0.437***
				(0.070)	(0.069)
Age				0.014***	0.013***
				(0.002)	(0.002)
Respondent born in country				0.160	0.160
				(0.141)	(0.139)
Household size				0.097***	0.090***
				(0.024)	(0.024)
Rural				−0.027	−0.032
				(0.075)	(0.074)
Working				0.245***	0.202***
				(0.064)	(0.063)
Religion important				0.744***	0.697***
				(0.066)	(0.065)
Completely satisfied with life				0.781***	0.698***
				(0.092)	(0.091)
USA	−0.145	−0.140	−0.138	−0.417***	−0.423***
	(0.104)	(0.104)	(0.105)	(0.105)	(0.104)
Japan	−1.586***	−1.584***	−1.672***	−1.677***	−1.553***
	(0.091)	(0.091)	(0.091)	(0.097)	(0.096)
Italy	−1.018***	−0.918***	−0.957***	−1.135***	−1.119***
	(0.106)	(0.106)	(0.107)	(0.107)	(0.106)
UK	−0.476***	−0.442***	−0.483***	−0.408***	−0.365***
	(0.105)	(0.105)	(0.106)	(0.106)	(0.105)
Observations	6194	6194	6194	6194	6194
R-squared	0.092	0.095	0.078	0.128	0.148

*Notes:* Results are from OLS regression models including country fixed effects. Data are from six representative samples of participants in Germany, Italy, Japan, the UK and the USA. Respondents were asked to indicate their own view with regard to the statement ‘Generally speaking, would you say that most people can be trusted, or that you can’t be too careful in dealing with people?’ by selecting a number from 0 to 10, where 0 = you can’t be too careful, and 10 = most people can be trusted. The response given is the dependent variable in all models.

***, **, * indicate *p* < 0.01, *p* < 0.05, *p* < 0.1, respectively.

**TABLE 6:** Second-mover sending, conditional cooperation and other-regarding preferences.

	0–3 CU			4–6 CU			7–10 CU		
	(1)	(2)	(3)	(4)	(5)	(6)	(7)	(8)	(9)
Conditional cooperation	−0.039***	−0.036***	−0.033***	0.003	0.003	0.005**	0.019***	0.019***	0.022***
	(0.003)	(0.003)	(0.003)	(0.003)	(0.003)	(0.003)	(0.003)	(0.003)	(0.003)
Altruism (standardized)			0.078***			0.063***			0.060***
			(0.003)			(0.003)			(0.003)
Risk proclivity			0.005***			0.003**			0.004***
			(0.001)			(0.001)			(0.001)
Individual characteristics		✓	✓		✓	✓		✓	✓
Observations	28,944	28,944	28,944	21,708	21,708	21,708	28,944	28,944	28,944
R-squared	0.035	0.043	0.146	0.004	0.007	0.103	0.011	0.015	0.096

*Notes:* Estimates of OLS regression models with individual-level observations. Dependent variable: proportion of available CU (i.e. tripled amount received, if any, plus 10 CU endowment) that *i* sends to their first mover when in the second mover role in the trust game. Estimates (1)–(3) use the set of observations of second-mover sending choices conditional on the amount sent by the first mover being 0, 1, 2 or 3 CU. Estimates (4)–(6) are, correspondingly, for observations conditioned on amounts sent of 4, 5 or 6 CU, and estimates (7)–(9) use observations conditioned on amounts sent of 7, 8, 9 or 10 CU. Standard errors are clustered at the individual level. All models control for country fixed effects. Online Appendix Table A pools data for all three first-mover sending ranges.

***, **, * indicate *p* < 0.01, *p* < 0.05, *p* < 0.1, respectively.

## Footnotes

1 The survey was conducted in the USA in June–July 2017, in Germany in July–August 2017 (with addition of 97 participants in June–July 2018), in Italy and the UK in June–July 2018, in Japan in January–February 2020, and in Luxembourg in May–June 2021.

2 We include only participants who responded to all parts of the questionnaire. We therefore drop 437 respondents with incomplete or missing information.

3 In some countries, additional games were played between the dictator game and the lottery task. For example, in the USA and Germany, participants made additional trust game first-mover choices following the dictator game, with the ethnic group or ethnicity plus income quintile of the second mover specified (Cetre et al. 2024).

4 Participants in the UK completed one additional risk-taking task, as well as an incentivized time preferences task that had higher payoffs, and could therefore earn up to £200 in total. However, while the maximum payout was higher in the UK, average payouts were similar compared to the other countries—as the probability of earning large amounts was low. In the UK, only 11 participants (1%) earned more than 40 CU.

5 Participants in Italy earned 13 CU on average, compared to 12 CU in Germany, the UK, Luxembourg and the USA, and 10 CU in Japan.

6 For example, when trustors send 0 CU, trustees can return up to 10 CU (using only their baseline endowment). When trustors send 1 CU, trustees can return up to 13 CU (three times the amount sent plus their baseline endowment). The ability of the second mover to make transfers to the first mover out of the second mover’s endowment is a departure from the rules used by Berg et al. (1995), and leads to some interesting effects, discussed below.

7 If a trustor sends 5 CU, then the trustee’s total budget is 25 CU (15 CU plus 10 CU endowment).

8 For example: ‘Please indicate how much (if anything) you would like to transfer to the joint project if, on average, each of the other group members contributes 0 CU.’

9 Recall that each participant would earn from the incentivized games the payoff associated with one randomly chosen game and role. A participant should therefore have perceived their own chance to receive payment as the receiver in the dictator game to be equal to the chance of being in any of the other games and roles. The participant in the dictator role itself might therefore send money with the motivation of altruism or fair sharing (see Camerer and Thaler 1995).

10 Bohnet and Zeckhauser (2004) and successor studies report evidence that experiment participants show greater aversion to having their return expectations be disappointed due to the choice of a human decision-maker than due to the outcome of a random draw. They coin the term ‘betrayal aversion’ to denote this. While the distinction is potentially relevant to all studies, including ours, in which there is a human second mover, Trustlab’s design includes no trust game with randomized second move, so we cannot distinguish between betrayal and risk aversion. This leaves open the possibility that some reluctance of first movers to send more could be related to fear of being disappointed but not captured by the risk aversion measure *per se*.

11 In the US survey, for example, the trustor sending choice was followed by 32 incentivized choices, by an implicit association task taking at least 5 minutes to complete, and by 18 items requiring survey responses, before the generalized trust question (followed by more than 60 additional survey items). The survey had the same overall structure in all countries.

12 We use the *shapley2* command in STATA 17.

13 Trustors in Germany sent an average of 6.5 CU of their endowment to trustees, compared to 6.3 CU in Luxembourg, 6.0 CU in the USA and Italy, 5.9 CU in the UK, and 5.3 CU in Japan.

14 When trustors send 5 CU, trustees have 25 CU available to return (3 × 5 CU plus their 10 CU endowment.)

15 Trustees in Luxembourg return 39% of their endowment when receiving 5 CU, compared to 38% in Germany and Italy, 37% in the UK and the USA, and 36% in Japan.

16 As shown in Online Appendix Subsection A, the distribution of expected returns is bimodal as 26% of trustors expected to receive 5 CU in return (the amount sent by the trustor), and 32% expected to receive 10 CU (the amount that makes the earnings of the two participants equal).

17 Online Appendix Figure A plots the amount sent on expected returns.

18 The 16.96% of those who sent less than half their endowment, and the 39.21% who sent half of their endowment, while expecting the receiver to return more than 5 CU, would have been forgoing the maximization of their own earnings if they held the stated beliefs confidently or were risk neutral. The 25.79% who sent more than half their endowment while reporting an expectation that the receiver would return less than 5 CU appeared to be making a loss-making or risk-loving choice. Added together, they made up about 29% of the overall sample. The 45.31% of senders of 5 CU and 24.26% of senders of more than 5 CU who reported expecting 5 CU in return, adding up to 27% of the sample, would, according to their self-reported expectation, have been sending despite only breaking even, suggesting, in practice, the likelihood that they had additional motives.

19 Hereafter, we use the same method to identify the share of variance attributable to specific variables or sets of variables, following Henderson et al. (2018) and Shorrocks (2013).

20 Some individual characteristics may not be significantly associated with the amount sent because of collinearity. We therefore enter each variable separately into regression models, controlling only for country fixed effects (results available on request). With the exception of labour force status, none of the personal characteristics shown in Table 3 are statistically significant when included in such a model. In a specification that includes only labour force status and country fixed effects, individuals who are currently working send 0.24 CU more.

21 Participant’s gender accounts for 25% of the total explained variance in model (4), and 64% is due to country fixed effects.

22 Four of the five correlates studied in this subsubsection are available for a subset of countries only, thus could not be integrated into the analysis of Tables 3 and 4 for the full sample. The remaining factor, redistributive preferences as indicated by the way participants would allocate their country’s tax burden among those in four different income classes, is available for all countries but studied in what follows as a set of four indicators, for which results stand out more clearly in separate regressions, as discussed next.

23 Sending in the trust game can be mainly intended to benefit both sender and receiver, which reduces society-wide income inequality if both have below average income, which is true of most given the skewness of the income distribution. Since the trustee can be assumed to probably have below-average income, a trustor of average income or above who wishes to reduce inequality disadvantaging the poor might also be more willing to countenance an outcome in which the trustee sends back little or nothing.

24 This is based on Shapley values, which respectively are 0.00001, 0.00029, 0.00003 and 0.00018 for models (1), (3), (5) and (7) of Online Appendix Table A.

25 Shapley values cannot be estimated for model (1), which includes too few variables.

26 These results are from regressions that include a control for at most one religion category, which is thus being compared to participants who report themselves to be of any of the remaining categories, which include Catholic, Protestant, Christian (other), Muslim, Jewish, Buddhist, not religious, and other. We report tests for each of three categories only: Catholic, Protestant and not religious. There is a clear overlap between the type of religion and the survey country, as 66% of Catholics live in Italy, 57% of Protestants in Germany, and 53% of atheists in Japan.

27 Shapley values are 0.00088, 0.00027 and 0.00079 for models (1)–(3), respectively, in Online Appendix Table A.

28 There is also a significant correlation between self-reported trust and marital status in the Trustlab data, as married individuals send 0.1 CU more (*p* = 0.0004).

29 Shapley values are 0.00061, 0.00141, 0.00329, 0.00183 and 0.00229 for models (1)–(5) of Online Appendix Table A.

30 A Kruskal–Wallis test suggests that self-reported trust differs significantly between countries (*p* < 0.0001). We use a Mann–Whitney test to examine whether differences between all country pairs are significant, and find that there is no significant difference between self-reported trust in Germany and the USA, but that differences are significant for all other country pairs (*p* < 0.0001).

31 Note again that the Trustlab trust game design allows the second mover to send to the first not only out of tripled receipts but also out of her initial endowment of 10 CU.

32 More precisely, there is no average direction of change for those for whom the best-fitting linear slope is zero. Most studies using the Fischbacher et *al*. (2001) elicitation procedure (for a meta-analysis, see Thöni and Volk 2018) have found a minority of subjects conforming to a pattern called ‘triangle’ or ‘hump’ shaped conditional cooperation, in which the participant chooses to contribute more as others do so, in the lower range of contributions by others, but then chooses to contribute less as others contribute more, in the higher range of their contributions. Since our $CC_{ix}$ variable is the estimated slope assuming no segmentation, ‘hump-shaped contributors’ can also have $CC_{ix}$ of approximately zero.

33 We first create two continuous variables: $l_{ix}$ indicates the share of the available money returned when ≥ 5 CU (or large amounts l) are sent by trustors; *s*_*ix*_ indicates the share of the available money returned when < 5 CU (or small amounts s) are sent by trustors. We then calculate the difference $d=l_{ix}-s_{ix}$ between the two. The binary measure of reciprocity proposed by Finan and Schecther (2012) is equal to 1 when d>1, and 0 otherwise.

34 In the rest of our paper, we use the conditional public goods game measure of reciprocity rather than that of Finan and Schechter (2012) so that decisions taken in the two roles of the trust game are reserved as targets of analysis rather than tools with which analysis is conducted. For another paper showing a strong correlation between trust game decisions and conditional cooperation in the public goods game, see Kim et al. (2022), who find that experimental participants treat information about one another’s trust game behaviours as reliable predictors of their conditional cooperation.

35 Online Appendix Tables F3,F4 and F5 provide more complete estimates of the regressions shown in Table 6, which also exhibit the coefficients of individual control variables, and Table A does the same for the fully pooled sample.

36 Note that the experimental literature has considered both fairness and altruism as potential explanations for sending in the dictator game, in the basic form of which (as also adopted in Trustlab) only the sender is provided with an endowment, hence there will be considerable inequality of game earnings between sender and receiver unless the sender transfers some money. In both the design by Berg et al. (1995) and that of Trustlab, trust game first and second movers begin with endowments of equal size, so first-mover sending motivated by other-regarding preferences can be motivated by altruism but not fairness concern (unless participants have knowledge of out-of-game relative wealth and poverty). First-mover altruism may be ‘super-charged’ or enhanced by efficiency preference (Charness and Rabin 2002), due to the experimenter’s tripling of sent funds. Both features are different for the second mover, who begins with inequality in her favour if the first mover has sent funds, but who has no efficiency reason for returning money in a one-shot interaction without reputational consequences. As for the significant but small positive correlation between risk proclivity and second-mover sending, it might be explained by the fact that a participant’s first- and second-mover sending are strongly correlated through the channel of expectations, and that first-mover sending and risk proclivity are in turn correlated due to the riskiness of the portion of sending attributable to an expectation of returns.

37 Data from Japan are simply weighted by 0.4 (i.e. 1000∕2500).

## APPENDIX A

**TABLE A1:** Variable definitions.

Variable	Definition/survey question	Coding	Type
Amount sent or first-mover sending (*x*)	Amount sent in the trust game (0,1,…,10)		Continuous (integer, 0–10)
Altruism (ORP)	Amount sent in the dictator game (0,1,…,10)		Continuous (integer, 0–10)
Risk tolerance	Based on a multiple lotteries task. Higher values indicate participants selecting riskier gambles.	Gambles numbered 1–6	Multivalued
Conditional cooperation	Slope of best-fit line through amounts contributed conditional on average amount contributed by others in conditional contribution task. Contributions and average contribution by others could be 0,1,…,10.		Continuous
Female	Respondent is female (0 = No; 1 = Yes)	Gender responses ‘Male’ and ‘Other’ coded 0 = No	Binary
Education	What is the highest level of education you have completed?		(in regressions that include controls for education, the controls are the binary variable ‘Other diploma’ and the binary variable ‘University,’ with ‘High school or less’ as the omitted category)
High school or less	*0* = *Less than high school; 1* = *High school diploma (low level); 2* = *High school diploma; 3* = *Some college, diploma, trades certificate or other post-school qualification; 4* = *Undergraduate degree; 5* = *Postgraduate degree*	High school or less (= 0, 1, or 2)	
Other diploma		Other diploma (= 3)	
University		University (= 4 or 5)	
Age	Age of the respondent		Continuous (integer)
Born in country	Respondent born in country	0= Non-native; 1 = Native	Binary
Household size	Number of people living in household of respondent		Continuous (integer)
Rural	Type of geographic area of respondent	Rural = 1 if response ≤ 1 Rural = 0 if response ≥ 2	Binary
	*0* = *Rural area;* *1* = *Village (*< *50,000 inhabitants);* *2* = *Town (50,000–200,000 inhabitants);* *3* = *Small metropolitan area (200,000–500,000 inhabitants);* *4* = *Medium-sized metropolitan area (500,000–1.5 million inhabitants);* *5* = *Large metropolitan area (more than 1.5 million inhabitants)*		
Working	Respondents were asked to indicate their current labour force status, selecting between: Employed, Self-employed, Unemployed and Outside the labour force	Working = 1 if employed or self-employed selected Working = 0 otherwise	Binary
Religion important	How important would you say religion is in your own life? (0 = Not important at all; 10 = Very important)	Religion important =1 if response > 5 Religion important = 0 otherwise	Binary
Completely satisfied with life	Overall, how satisfied are you with life as a whole these days? (0 = Not at all satisfied; 10 = Completely satisfied)	Completely satisfied with life = 1 if response = 10 = 0 for all other responses	Binary
Self-reported trust (response to generalized trust survey question)	Generally speaking, would you say that most people can be trusted, or that you can’t be too careful in dealing with people? (0 =You can’t be too careful; 10 = Most people can be trusted)		Continuous (integers)
Second-mover (trustee) sending: *y*/(3*x* + 10)	Proportion of the available amount (3x+10) sent by second mover in the trust game to the matched first mover, elicited for each possible first-mover sent amount (0,1,…, 10), where *x* = first-mover sent amount and y= second-mover sent amount		Semi-continuous (possible values range from 0 to 1, subject to integer values of *x* and *y*)
Redistributive preferences	The government currently raises a certain amount of revenues through taxes in order to sustain the current level of public spending. In your view, what would be the fair split of tax burden to sustain public spending?		Each variable is continuously valued over the 0–100% range (participants were required to adjust the four sliders until the sum of taxes collected given the income shares of those in the four categories equalled the overall share of income taxed in their country at the time of the survey)
Tax burden top 1% Tax burden next 9% Tax burden next 40% Tax burden bottom 50%	Please use the sliders below to tell us how much you think each of the following groups should pay as a percentage of their available resources. Each slider represents a segment of the population with a different income (top 1%, next 9%, next 40%, bottom 50%).		
Non-conformity	See the five pairs of statements among which participants chose, in Online Appendix A. Statements were taken from Blais and Hortala-Vallve (2021).	One option in each pair is coded as non-conformist. The measure is the share of the five items on which the participant chose the non-conformist option.	Multivalued (0, 0.2, 0.4, 0.6, 0.8, 1)
Religion	Religious affiliation of respondent.	1 = Not religious; 2 = Catholic; 3 = Protestant; 4 = Christian (other); 5 = Muslim; 6 = Jewish; 7 = Buddhist; 8 = Other	
No religion		No religion =1 if response is 1; no religion = 0 othersise	Binary
Catholic		Catholig = 1 if response is 2; Catholic = 0 otherwise	Binary
Protestant		Protestant = 1 if response is 3; Protestant = 0 otherwise	Binary
Marital status	Marital status		Binary
Married	*1* = *Married;* *2* = *Divorced;*	Married = 1 if response = 1 Married = 0 otherwise	
Divorced	*3* = *Never married;* *4* = *Widowed*	Divorced = 1 if response = 2 Divorced = 0 otherwise	
Personality traits Agreeableness Conscientiousness Openness Extraversion Neuroticism	Three questions for each trait from the Big Five Personality Inventory were included in the Trustlab surveys in Italy, Japan and the UK. See the questions in Online Appendix A. Each question was answered on a five-option scale ranging from strongly disagree to strongly agree.		Continuous (each trait is based on responses to three questions, as shown in Online Appendix A, where answers for each question range from strongly disagree = 1 to strongly agree = 5)
Against immigration	To what extent do you agree with the statement ‘Our culture is undermined by immigrants’, where 0 = strongly disagree and 10 = strongly agree?	Against immigration if > 5	Binary
Low political efficacy	To what extent do you agree with the statement ‘People like me don’t have any say about what government does’, where 0 = strongly disagree and 10 = strongly agree?	Low political efficacy if > 5	Binary
Social mobility is low	To what extent do you agree with the statement ‘There is not much opportunity to get ahead in society’, where 0 = strongly disagree and 10 = strongly agree?	Social mobility is low if > 5	Binary
