# Supplementary material for: The determinants of trust: findings from large, representative samples in six OECD countries
Source: Economica. Author manuscript; Available in PMC 2025 Sep 26. (PMC12456668; doi:10.1111/ecca.12549)
Supplement: Online Appendices [file NIHMS2043840-supplement-Online_Appendices.pdf]

For online publication only

Appendix to "The determinants of trust: Findings from large, representative samples in six OECD countries"

Authors: Roxanne Kovacs, Maurice Dunaiski, Matteo M Galizzi, Gianluca Grimalda, Rafael Hortala-Vallve and Louis Putterman

A Experimental instructions

Screen 1:

Welcome! Our research team invites you to participate in a quick online study on decision making. The aim of this study is to learn more about how we as human beings behave – how do we make decisions? How do we interact with one another when faced with different choices? How do we feel about the people and institutions around us? To find this out, you will be participating in different tasks: In the first part, you will participate in five simple tasks, in anonymous interaction with one or more other people. In the second part, you are going to sort different sets of words. In the third part, we ask you to answer a few questions about yourself and your opinions. The whole study should take you about 30 minutes. Note that you should complete this study in one sitting, without any extensive period of inactivity. For best results, minimise distractions and close other programs. You can participate in the study via your laptop computer or tablet (we support recent iPads). If you are having trouble accessing the platform, we advise you to switch to Google Chrome. If problems persist, please contact GMI, specifying your device model and browser.

By participating in the study's tasks, you can earn an amount of up to [currency amount: United States: "40 dollars"]. This amount will depend on the decisions you make together with the other participants during the study's tasks. At the end of the study, one of the several tasks you have completed will be randomly selected. The amount of money you will receive will correspond to your earnings in this selected task. Your decisions will also affect the earnings that other people will receive! You will receive your money at the end of the study via Paypal. Your payment will be processed after your decisions and those of other participants are collected. Because other participants may not be online at the same time as you, the calculation of your earnings may take up to 48 hours.

The data gathered in this study is subject to national privacy protocols. We will use it for research purposes only.

Screen 2 – SECTION 1: TASKS

We will start by giving you five tasks. Note that each task may include several different

decisions. This is the part of the study that will allow you to earn additional money. Each of these decisions may determine your final payments. At the beginning of each task, you may be grouped with other study participants. All participants in this study are from [country name (United States: "the United States"] like you. In each task, the other participants you are grouped with will be different: the same person will never be in your group more than once.

How will your earnings be calculated? Your earnings in each task will depend on your and the other participants' decisions. At the end of the study, one of the five tasks you have completed will be randomly selected. The amount of money you will receive will correspond to your earnings in one of the decisions in this selected task. In short, each task may determine your final payoff!

Screen 3 – TASK 1: INTRODUCTION

In the first task, two people participate: Participant A and Participant B. As mentioned before, this other participant also lives in [country name (United States: "the United States"].

These are the rules of the task:

• At the beginning of the task, both participants receive [currency amount, United States: "10 dollars"].

• Participant A has the option to transfer none, part or all of his or her [currency amount, United States: "10 dollars"] to Participant B.

• Whatever amount Participant A sends is multiplied by 3.

• Participant B, after receiving the transfer of Participant A, has to decide how much money, if any, he or she wants to send back to Participant A.

You are asked to make decisions in both role A and B. Which role you will be assigned to for payment will be determined randomly. In either case, your interaction will be with a person who gets randomly assigned to the other role.

Screen 4 – TASK 1: TEST SIMULATION

This is not the real task yet, but a simulation to help you understand the rules better. You can use the test screen below to experiment with the different choices of the two participants.

Between each test, click the "reset to zero" button below to reset the calculator.

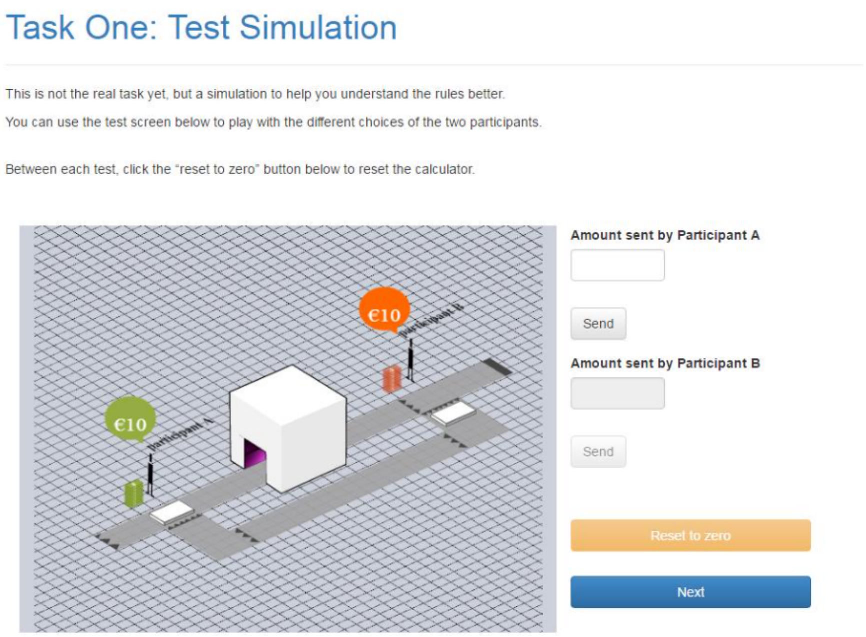

Screen 5 – TASK 1: REAL TASK (PARTICIPANT A)

Now the real task 1 starts. Once you have made your decision and clicked the "Next" button, you cannot return to this screen.

Suppose you are selected to be in the role of Participant A. You have [currency amount, United States: "10 dollars"] in your possession. How much (if any) do you want to send to Participant B? Please enter a number from 0 to 10:

Screen 6 – TASK 1: REAL TASK (PARTICIPANT B)

Now, suppose you are selected to be in the role of Participant B. On this screen you will make the decisions that will count if you are selected for that role. Once you have made your decision and clicked the "Next" button, you cannot return to this screen. As always, your initial endowment is [currency amount, United States: "10 dollars"]. Remember that Participant A also starts with an endowment of [currency amount, United States: "10 dollars"].

If Participant A sends you any of the amounts listed in the table below, how much money (if any) do you want to send back to Participant A? All of your choices below can impact how much money you and the other participant will receive at the end of the study.

• If Participant A sends you [currency amount, United States: "0 dollars"] (your total endowment is now [currency amount, United States: "10 dollars"]. How much will you send back to Participant A:

• If Participant A sends you [currency amount, United States: "1 dollar"] (your total endowment is now [currency amount, United States: "13 dollars"]. How much will you send

back to Participant A:

• If Participant A sends you [currency amount, United States: “2 dollars”] (your total endowment is now [currency amount, United States: “16 dollars”]. How much will you send back to Participant A:

• If Participant A sends you [currency amount, United States: “3 dollars”] (your total endowment is now [currency amount, United States: “19 dollars”]. How much will you send back to Participant A:

• If Participant A sends you [currency amount, United States: “4 dollars”] (your total endowment is now [currency amount, United States: “22 dollars”]. How much will you send back to Participant A:

• If Participant A sends you [currency amount, United States: “5 dollars”] (your total endowment is now [currency amount, United States: “25 dollars”]. How much will you send back to Participant A:

• If Participant A sends you [currency amount, United States: “6 dollars”] (your total endowment is now [currency amount, United States: “28 dollars”]. How much will you send back to Participant A:

• If Participant A sends you [currency amount, United States: “7 dollars”] (your total endowment is now [currency amount, United States: “31 dollars”]. How much will you send back to Participant A:

• If Participant A sends you [currency amount, United States: “8 dollars”] (your total endowment is now [currency amount, United States: “34 dollars”]. How much will you send back to Participant A:

• If Participant A sends you [currency amount, United States: “9 dollars”] (your total endowment is now [currency amount, United States: “37 dollars”]. How much will you send back to Participant A:

• If Participant A sends you [currency amount, United States: “10 dollars”] (your total endowment is now [currency amount, United States: “40 dollars”]. How much will you send back to Participant A:

Screen 7 – TASK 1: REAL TASK (HYPOTHETICAL SCENARIO)

You have just had made decisions as Participant A and Participant B. The following question is about your expectations of other people’s decisions. You are not actually deciding as Participant A or Participant B, and this decision will not affect your earnings.

We want you to imagine the following scenario: Imagine you sent [currency amount, United States: “5 dollars”], so Participant B receives [currency amount, United States: “15 dollars”], making his or her total budget [currency amount, United States: “25 dollars”]. Participant

B has no information about your identity. What amount would you expect Participant B to return to you? Please enter a number from 0 to 25.

Screen 8 – THANK YOU

Thank you very much for entering your choice. We have recorded your decision. Now, please proceed to the second task.

Screen 9 – TASK TWO: INTRODUCTION

In the second task, groups of 4 participants (yourself and 3 other people) are formed. Remember, the participants in this group are different from the person you interacted with in the previous task. However, they all live in [country name (United States: "the United States"].

These are the rules: • At the beginning, each group member has [currency amount, United States: "10 dol- lars"].

• Every group member has to choose how much of these [currency amount, United States: "10 dollars"] he or she wants to keep and how much he or she wants to transfer into a joint project.

• The total amount transferred to the joint project is multiplied by 1.6.

• At the end, the money in the joint project will be re-divided and split equally between all 4 group members (including yourself).

Screen 10 – TASK TWO: TEST SIMULATION

This is not the real task yet, but a simulation to help you understand the rules better. You can use the test screen below to experiment with the different choices of the four participants.

Whenever you are ready to proceed to the real task, click next.

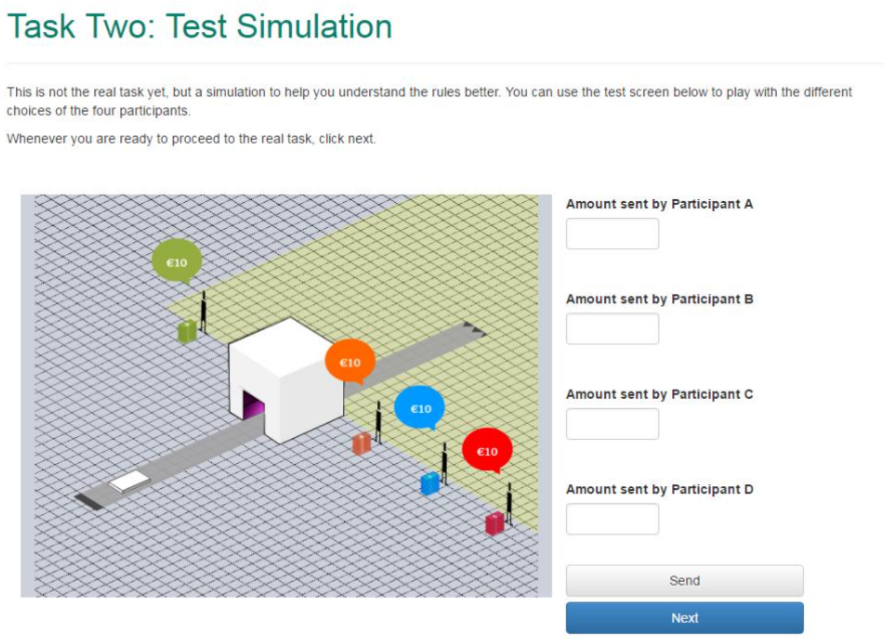

Screen 11 – TASK TWO: REAL TASK

Now the real task starts. Once you have made your decision and clicked the "Next" button, you cannot return to this screen. You have [currency amount, United States: "10 dollars"] in your possession. You may choose to keep this money, or choose to invest some (or all) of it in the joint project. How much (if any) do you want to transfer to the project?

Screen 12 – TASK TWO: REAL TASK CONTINUED

Now imagine that this time, you find out how much money the other three members of your group are investing in the joint project. All of your choices below can impact how much money you will receive at the end of the study.

Please indicate how much (if any) you would like to transfer to the joint project:

• if on average, each of the other group members contributes [currency amount, United States: "0 dollars"]:

• if on average, each of the other group members contributes [currency amount, United States: "1 dollar"]:

• if on average, each of the other group members contributes [currency amount, United States: "2 dollars"]:

• if on average, each of the other group members contributes [currency amount, United States: "3 dollars"]:

• if on average, each of the other group members contributes [currency amount, United States: “4 dollars”]:

• if on average, each of the other group members contributes [currency amount, United States: “5 dollars”]:

• if on average, each of the other group members contributes [currency amount, United States: “6 dollars”]:

• if on average, each of the other group members contributes [currency amount, United States: “7 dollars”]:

• if on average, each of the other group members contributes [currency amount, United States: “8 dollars”]:

• if on average, each of the other group members contributes [currency amount, United States: “9 dollars”]:

• if on average, each of the other group members contributes [currency amount, United States: “10 dollars”]:

Screen 13 – THANK YOU

Thank you very much for entering your choice. We have recorded your decision. Again, your payoff will depend on the actions of the other participants. Now, please proceed to the third task.

Screen 14 – TASK THREE: INTRODUCTION

The third task involves two participants – Participant A and Participant B. Remember, the other participant is different from the ones you interacted with in the previous two tasks. However, he or she also lives in [country name (United States: “the United States”].

These are the rules:

• At the beginning, Participant A receives [currency amount, United States: “10 dollars”].

• Participant B does not receive any money – he or she has [currency amount, United States: “0 dollars”].

• Participant A must now decide if he or she wants to transfer any of his or her [currency amount, United States: “10 dollars”] to Participant B.

This transfer is not multiplied by any number and Participant B cannot transfer any amount back to Participant A. Your role (Participant A or Participant B) will be determined later. We ask you to make a choice as A in case this is your role. B has no decision to make. Remember that someone will be assigned to role B and that person’s payment will be affected

by your decision as A. Because this task is simple, there will be no simulator to test out different choices.

Screen 15 – TASK THREE: REAL DECISION

This is the real third task. Once you have made your decision and clicked the "Next" button, you cannot return to this screen. Suppose that you are selected to be in the role of Participant A. You have [currency amount, United States: "10 dollars"] in your possession. How much (if any) do you want to transfer to Participant B?

Screen 16 - THANK YOU

Thank you very much for entering your choice. We have recorded your decision. Now, please proceed to the fourth task, which will be similar to Task 1. [. . .]

Screen 32 – TASK FIVE: INTRODUCTION

In this task you have the option to choose from six different gambles. In each gamble, you can win one out of two amounts. You must select one and only one of these gambles. Each gamble has two possible outcomes: outcome A and outcome B. Only one of these outcomes will occur. The gamble works as a random draw, comparable to a coin toss. Like in a coin toss, each possible outcome has a 50% chance of occurring.

Your compensation for this part of the study will be determined by:

1. Which of the six gambles you select. This is your choice. 2. Which of the two possible outcomes occur. This is determined by chance. The random draw is conducted by our computer. Either outcome has the same probability of occurring. The gamble selection table below shows your possible options. You will be asked to choose one of these gambles.

Gamble	Outcome	Payoff	Probabilities	Choice
1	A	8	50%	●
	B	8	50%	
2	A	7	50%	●
	B	10	50%	
3	A	6	50%	●
	B	12	50%	
4	A	5	50%	●
	B	14	50%	
5	A	4	50%	●
	B	16	50%	
6	A	1	50%	●
	B	19	50%	

Examples:

- For instance, if you choose Gamble 2, you will earn 7 dollars if outcome A occurs, or 10

dollars if outcome B occurs.

• If you choose Gamble 5, you will earn 4 d ollars if outcome A occurs, or 16 dollars if outcome B occurs.

• If you choose Gamble 1, you will earn 8 dollars, regardless of which outcome occurs.

Screen 33 – TASK FIVE: REAL TASK

Now the real task 5 starts. Once you have made your decision and clicked the "Next" button, you cannot return to this screen. These are the six gambles from which you can choose. If this task is chosen for payment, then your earnings will depend on the gamble you choose and the outcome of the gamble. Please select the gamble of your choice.

Gamble	Outcome	Payoff	Probabilities	Choice
1	A	8	50%	●
	B	8	50%	
2	A	7	50%	●
	B	10	50%	
3	A	6	50%	●
	B	12	50%	
4	A	5	50%	●
	B	14	50%	
5	A	4	50%	●
	B	16	50%	
6	A	1	50%	●
	B	19	50%	

Screen 34 – TASK FIVE: THANK YOU

Thank you once again! You have completed all tasks in this section and we have recorded all your choices. Let's go to the second section of the study, where you will be asked to sort a number of words.

B "Big Five" personality traits

The following items were used to measure participants "Big Five" personality traits. Traits are captured in factor scores using integrated principal factor analysis, as set out in (John & Srivastava 1999). Following standard procedure, factor scores for each of the five personality traits are based on responses to three survey questions each. For each of the five traits, we initially calculate factor loadings – which capture the degree of correlation between the item (the survey question) and the factor (the underlying construct of interest, say agreeableness). Factor scores for each personality trait are equal to the sum of Likert-scale responses to each question (which run from 1=strongly disagree to 5=strongly agree) weighed by the item's

factor loading (which runs from -1 to +1). The analysis is performed on the combined sample of countries.

Participants were asked about how strongly they agree or disagree with each statement (strongly disagree, disagree a little, neither agree nor disagree, agree a little, strongly agree):

Agreeableness:

1. I see myself as someone who is sometimes somewhat rude to others
2. I see myself as someone who has a forgiving nature
3. I see myself as someone who is considerate and kind to others

Conscientiousness:

1. I tend to be lazy
2. I see myself as someone who does a thorough job
3. I see myself as someone who does things effectively and efficiently

Neuroticism:

1. I see myself as someone who is relaxed, handles stress well
2. I see myself as someone who gets nervous easily
3. I see myself as someone who worries a lot

Extraversion:

1. I see myself as someone who is reserved
2. I see myself as someone who is outgoing, sociable
3. I see myself as someone who is communicative, talkative

Openness:

1. I see myself as someone who values artistic experiences
2. I see myself as someone who has an active imagination
3. I see myself as someone who is original, comes up with new ideas

C Measuring non-conformity

In each of the following 5 pairs of statements, you will be asked to decide which statement you agree most on, statement A or statement B. For each pair of statements, which of the two statements do you agree most on?

Pair 1:

1. Statement A: "It's best for everyone if people try to fit in instead of acting in unusual ways"
2. Statement B: 'People should be encouraged to express themselves in unique and possibly unusual ways"

Pair 2:

1. Statement A: "People should not try to understand how society works but just accept the way it is"
2. Statement B: "People should constantly try to question why things are the way they are"

Pair 3:

1. Statement A: "People should be guided more by their feelings and less by the rules"
2. Statement B: "The only way to stay out of trouble is to respect the established rules of society"

Pair 4:

1. Statement A: "In the long run our cultural and ideological differences will make us a healthier, more creative, and stronger society"
2. Statement B: "It is unlikely that this country will survive in the long run unless we can overcome our differences and disagreements"

Pair 5:

1. Statement A: "Young people sometimes get rebellious ideas but as they grow up they ought to get over them and settle down"
2. Statement B: "If some people don't occasionally come up with rebellious ideas there would be less progress in the world"

D Trust

D.1 Expected returns

Figure D1: Expected returns in the investment game

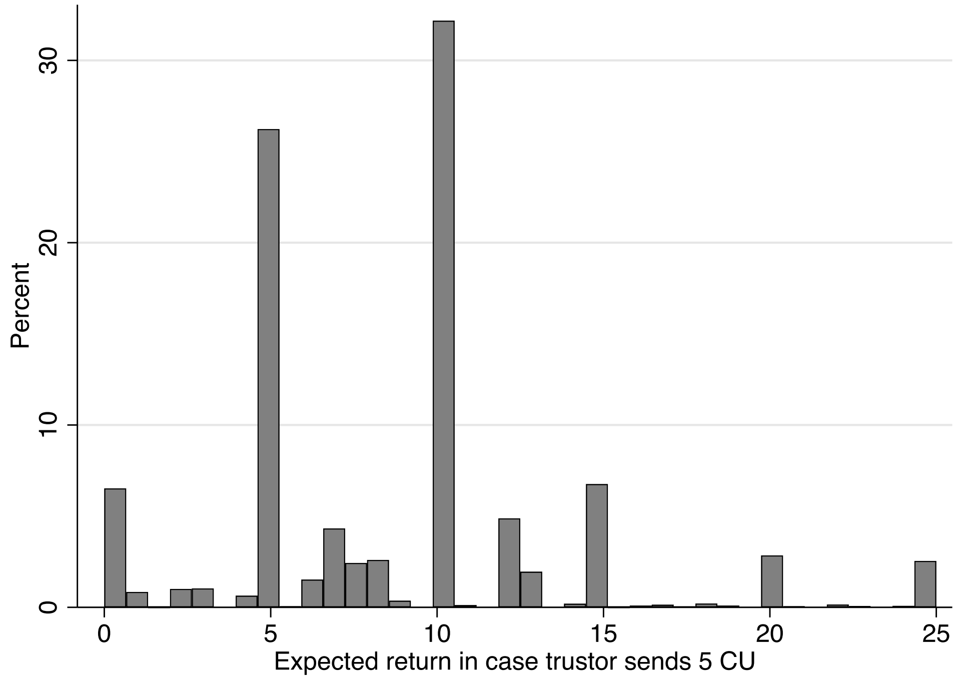

Note: Trustors were asked how much they expect to receive back from trustees in a situation where they transferred 5 CU to the trustee. If trustors send 5 CU, the amount is tripled and the second mover can return up to 25 CU (15 CU + 10 CU endowment).

Figure D2: Amount sent and expected returns

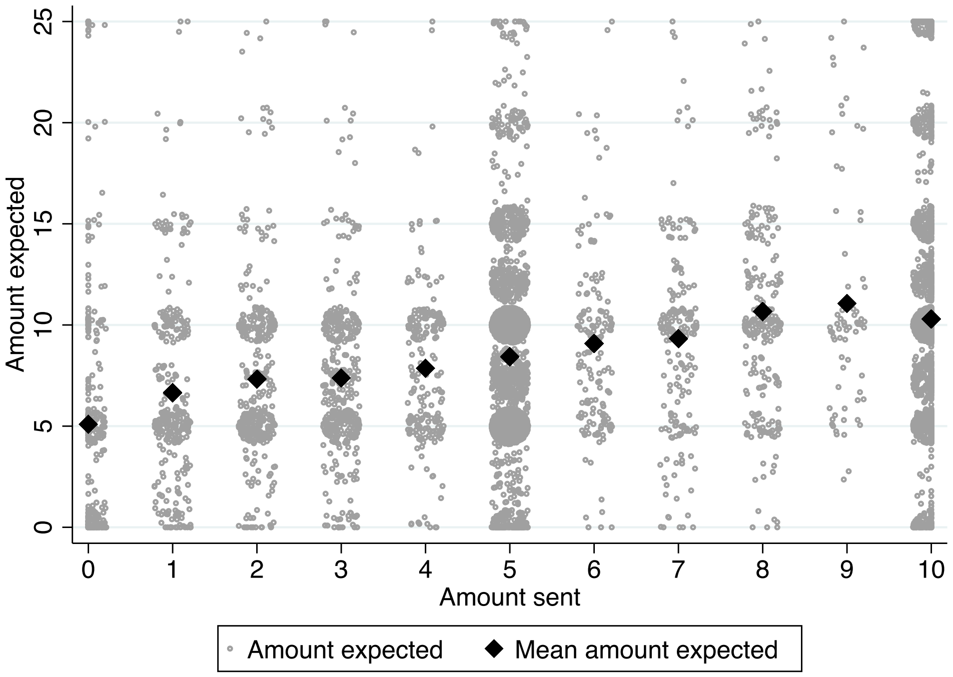

Note: The figure plots the amount sent in the investment game against the amount expected in return. The black diamond-shaped markers show the average (mean) amount expected back, for each amount sent. Data are from a representative sample of respondents in Germany, Italy, Japan, Luxembourg, the UK and the USA.

D.2 Preferences for redistribution

Table D1: Amount sent and preferences for redistribution

VARIABLES	(1)	(2)	(3)	(4)	(5)	(6)	(7)	(8)
Tax burden top 1%	0.003	0.066**						
	(0.036)	(0.033)						
Tax burden next 9%			0.055	0.134***				
			(0.036)	(0.033)				
Tax burden next 40%					-0.021	-0.042		
					(0.036)	(0.033)		
Tax burden bottom 50%							-0.035	-0.126***
							(0.036)	(0.033)
Expected return (standardised)		0.527***		0.516***		0.509***		0.518***
		(0.035)		(0.035)		(0.035)		(0.035)
Altruism ORP (standardised)		0.939***		0.931***		0.927***		0.931***
		(0.035)		(0.035)		(0.035)		(0.035)
Risk proclivity				0.147***		0.146***		0.146***
				(0.019)		(0.019)		(0.019)
Constant	6.513***	6.351***	6.510***	5.923***	6.514***	5.938***	6.509***	5.915***
	(0.096)	(0.088)	(0.096)	(0.104)	(0.096)	(0.104)	(0.096)	(0.105)
Observations	7,236	7,236	7,236	7,236	7,236	7,236	7,236	7,236
R-squared	0.022	0.167	0.022	0.175	0.022	0.173	0.022	0.175

Note: Results are from OLS regression models. The dependent variable in all models is the amount sent by the first mover. Data are from a representative sample of respondents in Germany, Italy, Japan, Luxembourg, the UK and the USA. All models control for country fixed effects.

D.3 Non-conformity

Table D2: Amount sent and non-conformity

VARIABLES	(1)	(2)
Non-conformity	0.547	0.612*
	(0.350)	(0.326)
Expected return (standardised)		0.793***
		(0.091)
Altruism ORP (standardised)		0.634***
		(0.091)
Risk proclivity		0.064
		(0.053)
Constant	5.587***	5.347***
	(0.233)	(0.263)
Observations	1,034	1,034
R-squared	0.002	0.141

Note: Results are from OLS regression models. The dependent variable in all models is the amount sent by the first mover. Data are from a representative sample of respondents in the UK. 61% of respondents feel little pressure to conform to community norms (SD=0.26).

D.4 Religion

Table D3: Amount sent and religion in Germany, Italy, Japan and the UK

VARIABLES	(1)	(2)	(3)	(4)	(5)	(6)
Catholic	-0.257*			-0.270**		
	(0.137)			(0.125)		
No religion		0.130			0.248***	
		(0.090)			(0.082)	
Protestant			-0.118			-0.157
			(0.158)			(0.144)
Expected return (standardised)				0.548***	0.552***	0.544***
				(0.042)	(0.042)	(0.042)
Altruism ORP (standardised)				0.954***	0.958***	0.958***
				(0.041)	(0.041)	(0.041)
Risk proclivity				0.153***	0.152***	0.152***
				(0.023)	(0.023)	(0.023)
Constant	6.575***	6.459***	6.546***	5.974***	5.806***	5.955***
	(0.102)	(0.104)	(0.106)	(0.115)	(0.117)	(0.119)
Observations	5,184	5,184	5,184	5,184	5,184	5,184
R-squared	0.025	0.024	0.024	0.187	0.187	0.186

Note: Results are from OLS regression models. The dependent variable in all models is the amount sent by the first mover. Data are from a representative sample of respondents in Germany, Italy, Japan and the UK. All models control for country fixed effects.

D.5 Marital status

Table D4: Amount sent and marital status in Italy

VARIABLES	(1)	(2)	(3)	(4)
Married	-0.080		-0.157	
	(0.186)		(0.172)	
Divorced		-0.148		-0.073
		(0.339)		(0.313)
Expected return (standardised)			0.430***	0.429***
			(0.086)	(0.086)
Altruism ORP (standardised)			0.967***	0.964***
			(0.100)	(0.100)
Risk proclivity			0.023	0.020
			(0.054)	(0.054)
Constant	6.050***	6.018***	5.917***	5.845***
	(0.138)	(0.097)	(0.195)	(0.178)
Observations	998	998	998	998
R-squared	0.000	0.000	0.151	0.151

Note: Results are from OLS regression models. The dependent variable in all models is the amount sent by the first mover. Data are from a representative sample of respondents in Italy.

D.6 Personality traits

Table D5: Amount sent and personality traits

VARIABLES	(1)	(2)	(3)	(4)	(5)
Agreeableness	0.012				
	(0.031)				
Conscientiousness		0.003			
		(0.031)			
Neuroticism			-0.064***		
			(0.023)		
Extraversion				0.050*	
				(0.026)	
Openness					0.053**
					(0.026)
Constant	5.881***	5.904***	6.102***	5.767***	5.530***
	(0.143)	(0.215)	(0.117)	(0.126)	(0.214)
Observations	4,186	4,186	4,186	4,186	4,186
R-squared	0.013	0.013	0.014	0.013	0.014

Note: Results are from OLS regression models. All models control for country fixed effects. The dependent variable in all models is the amount sent by the first mover. Data are from a representative sample of respondents in Italy, Japan and the UK.

Table D6: Amount sent and personality traits

VARIABLES	(1)	(2)	(3)	(4)	(5)
Agreeableness	-0.002				
	(0.028)				
Conscientiousness		-0.008			
		(0.029)			
Neuroticism			-0.027		
			(0.021)		
Extraversion				-0.011	
				(0.024)	
Openness					0.011
					(0.023)
Expected return (standardised)	0.515***	0.515***	0.512***	0.516***	0.514***
	(0.047)	(0.047)	(0.047)	(0.047)	(0.047)
Altruism ORP (standardised)	0.991***	0.991***	0.989***	0.992***	0.990***
	(0.046)	(0.046)	(0.046)	(0.046)	(0.046)
Risk proclivity	0.164***	0.164***	0.162***	0.164***	0.163***
	(0.027)	(0.027)	(0.027)	(0.027)	(0.027)
Constant	5.461***	5.501***	5.532***	5.488***	5.376***
	(0.149)	(0.207)	(0.131)	(0.136)	(0.206)
Observations	4,186	4,186	4,186	4,186	4,186
R-squared	0.182	0.182	0.182	0.182	0.182

Note: Results are from OLS regression models. All models control for country fixed effects. The dependent variable in all models is the amount sent by the first mover. Data are from a representative sample of respondents in Italy, Japan and the UK.

D.7 Political views

Table D7: Amount sent and political views

VARIABLES	(1)	(2)	(3)	(4)	(5)	(6)
Against immigration	-0.086			-0.055		
	(0.086)			(0.078)		
Low political efficacy		0.076			0.153*	
		(0.089)			(0.082)	
Social mobility is low			-0.031			0.098
			(0.076)			(0.070)
Expected return (standardised)				0.503***	0.508***	0.514***
				(0.039)	(0.039)	(0.036)
Altruism ORP (standardised)				0.956***	0.954***	0.925***
				(0.038)	(0.038)	(0.035)
Risk proclivity				0.133***	0.146***	0.139***
				(0.022)	(0.021)	(0.020)
Observations	5,870	6,007	7,045	5,870	6,007	7,045
R-squared	0.020	0.020	0.021	0.180	0.180	0.172

Note: Results are from OLS regression models. All models control for country fixed effects. The dependent variable in all models is the amount sent by the first mover. Data in columns 1,2,4 and 5 are from a representative sample of respondents in Germany, Italy, Japan, the UK and the USA. Data in columns 3 and 6 are from a representative sample of respondents in Germany, Italy, Japan, Luxembourg, the UK and the USA

E Self-reported trust

Figure E1: Amount sent in the trust game and self-reported trust

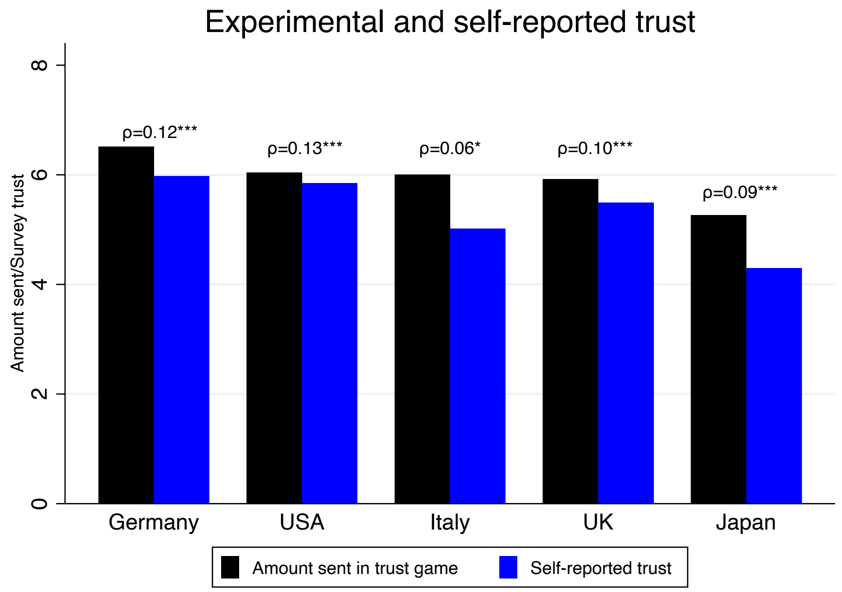

Note: The figure shows the country-level averages of the amount sent in the trust game and responses to the trust survey question, as well as the Pearson correlation between the two. Respondents were asked to respond on a scale from 0 to 10, indicating where they place their own view with regard to the statement "Generally speaking, would you say that most people can be trusted, or that you can't be too careful in dealing with people?" where 0 = you can't be too careful and 10 = most people can be trusted. As the amount sent ranges between 0 CU and 10 CU, survey and experimental trust can be plotted on the same scale. Data are from a representative sample of participants in Germany, Italy, Japan, the UK and the US. A Mann-Whitney test indicates that here is no significant difference between self-reported trust in Germany and the USA, but that differences are significant for all other country pairs ($p < 0.0001$). Kruskal-Wallis test suggests that self-reported trust differs significantly between countries ($p < 0.0001$).

Figure E2: Self-reported trust in Germany

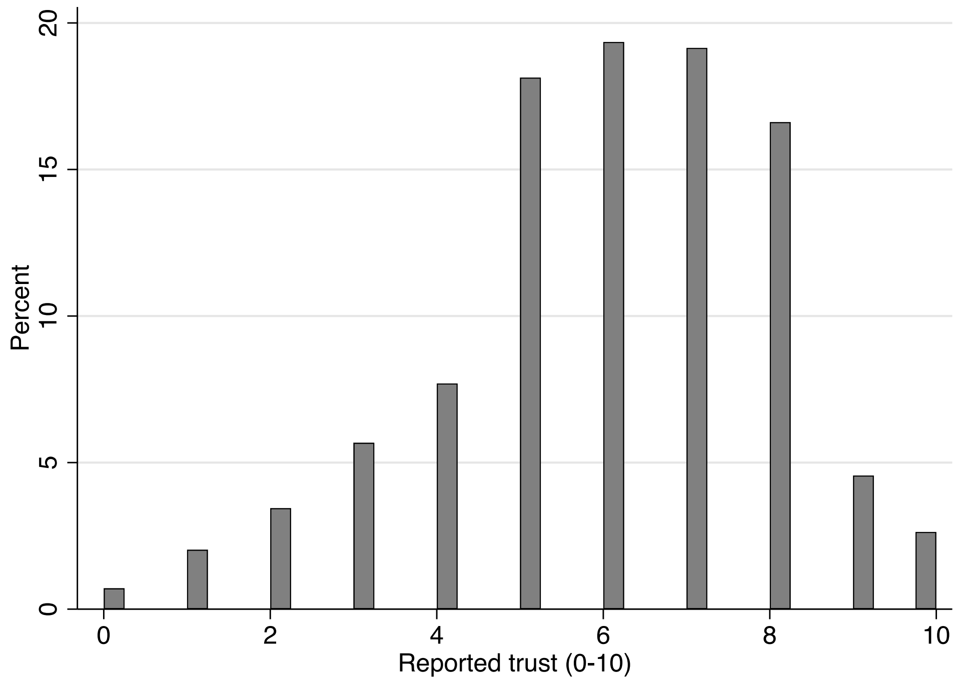

Note: Participants were asked indicate to what extend they agree with the statement "Generally speaking, would you say that most people can be trusted or that you cannot be too careful in dealing with people?", on a scale from 0-10.

Figure E3: Self-reported trust in Italy

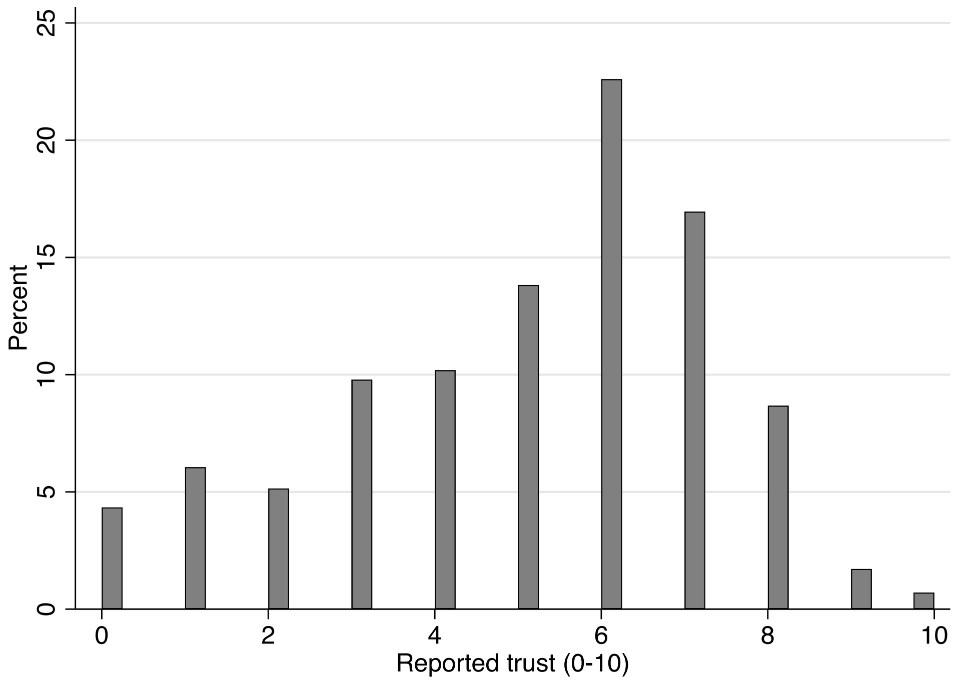

Note: Participants were asked indicate to what extend they agree with the statement "Generally speaking, would you say that most people can be trusted or that you cannot be too careful in dealing with people?", on a scale from 0-10.

Figure E4: Reported trust in Japan

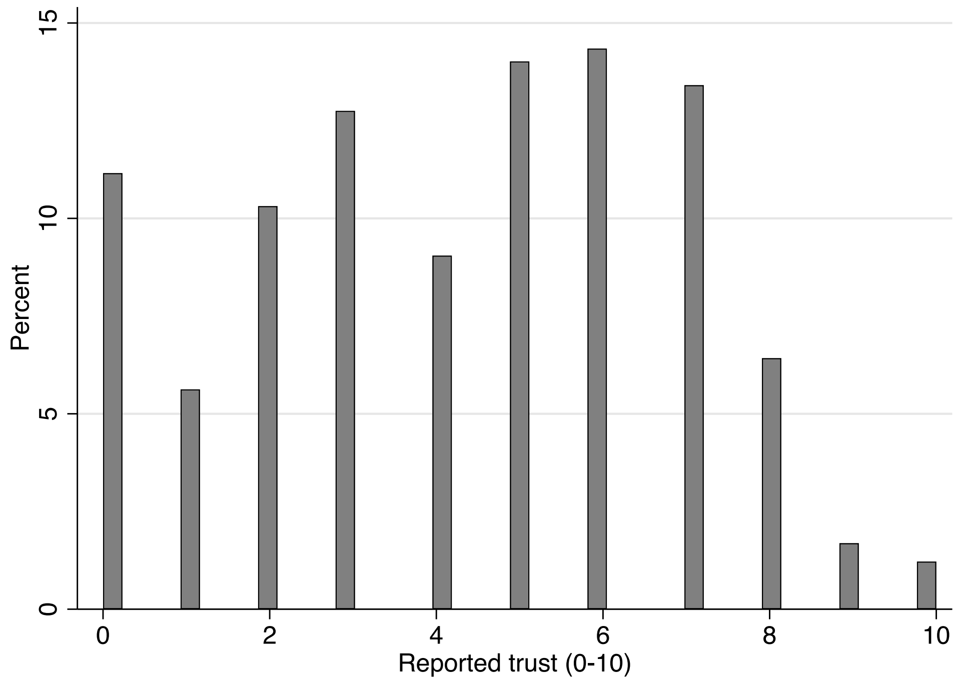

Note: Participants were asked indicate to what extend they agree with the statement "Generally speaking, would you say that most people can be trusted or that you cannot be too careful in dealing with people?", on a scale from 0-10.

Figure E5: Self-reported trust in the UK

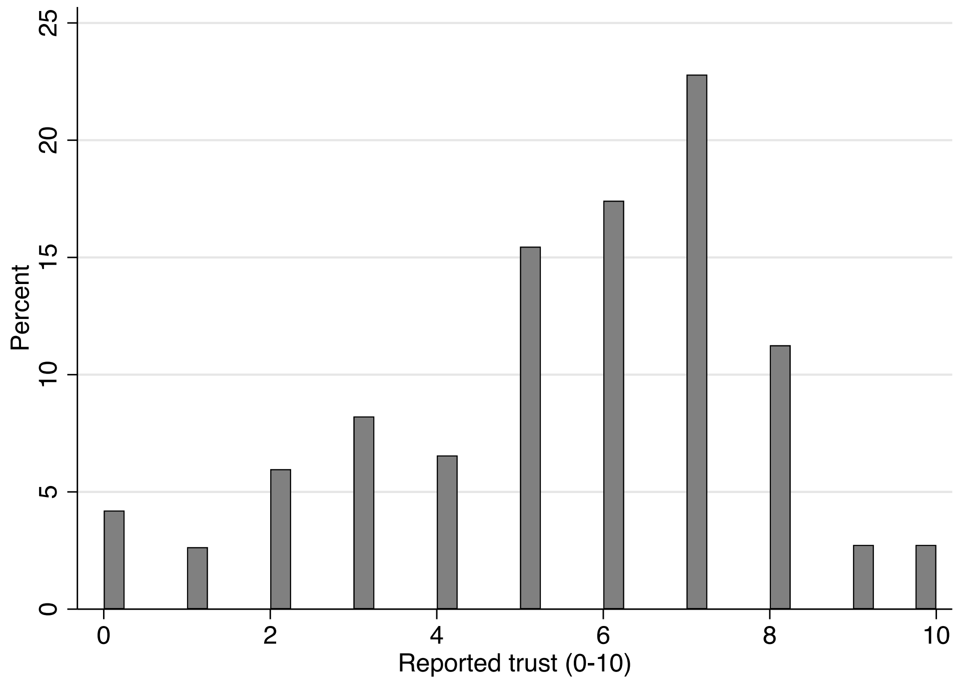

Note: Participants were asked indicate to what extend they agree with the statement "Generally speaking, would you say that most people can be trusted or that you cannot be too careful in dealing with people?", on a scale from 0-10.

Figure E6: Reported trust in the USA

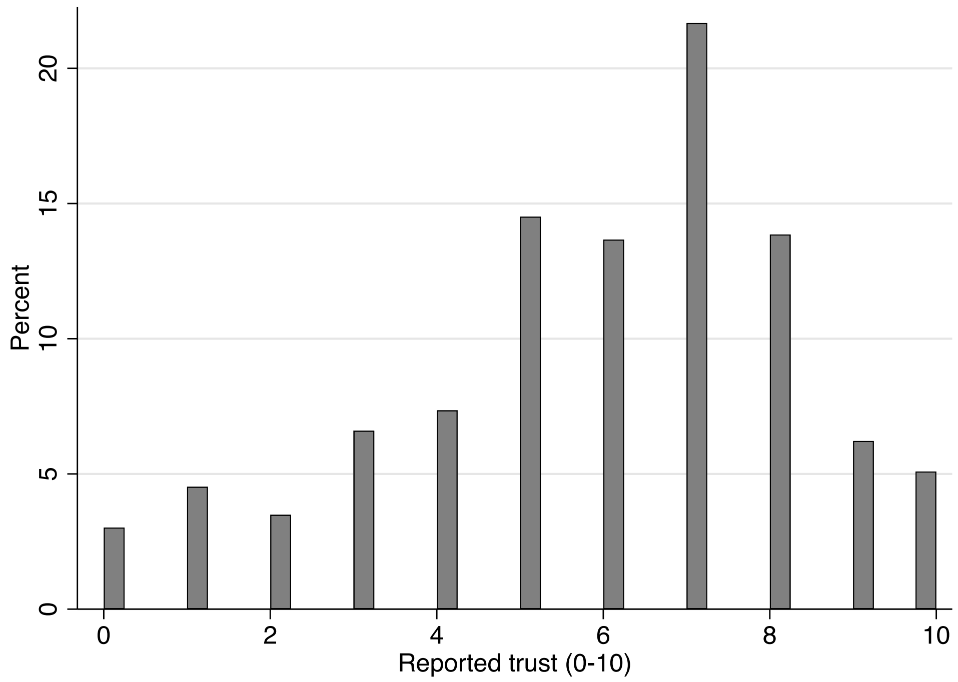

Note: Participants were asked indicate to what extend they agree with the statement “Generally speaking, would you say that most people can be trusted or that you cannot be too careful in dealing with people?”, on a scale from 0-10.

F Trustworthiness

F.1 Reciprocity

Table F1: Second-mover sending: Conditional cooperation and altruism

	(1)<br>0 CU	(2)<br>1 CU	(3)<br>2 CU	(4)<br>3 CU	(5)<br>4 CU	(6)<br>5 CU	(7)<br>6 CU	(8)<br>7 CU	(9)<br>8 CU	(10)<br>9 CU	(11)<br>10 CU
Panel A:											
Cond. cooperation	-0.075***	-0.044***	-0.025***	-0.012***	-0.004	0.003	0.009***	0.015***	0.018***	0.022***	0.023***
	(0.003)	(0.003)	(0.002)	(0.002)	(0.002)	(0.002)	(0.002)	(0.002)	(0.002)	(0.002)	(0.003)
Shapley values	0.064	0.040	0.017	0.004	0.001	0.000	0.002	0.004	0.007	0.010	0.011
Observations	7,236	7,236	7,236	7,236	7,236	7,236	7,236	7,236	7,236	7,236	7,236
R-squared	0.074	0.050	0.025	0.009	0.005	0.004	0.006	0.008	0.010	0.014	0.014
Panel B:											
Altruism (std.)	0.103***	0.0818***	0.0713***	0.0676***	0.0652***	0.0645***	0.0609***	0.0608***	0.0589***	0.0592***	0.0594***
	(0.00336)	(0.00251)	(0.00227)	(0.00222)	(0.00227)	(0.00229)	(0.00228)	(0.00237)	(0.00237)	(0.00241)	(0.00244)
Shapley values	0.118	0.130	0.122	0.115	0.104	0.100	0.091	0.084	0.079	0.077	0.076
Observations	7,236	7,236	7,236	7,236	7,236	7,236	7,236	7,236	7,236	7,236	7,236
R-squared	0.127	0.139	0.129	0.118	0.107	0.102	0.094	0.087	0.082	0.079	0.078

Note: Results are from OLS regression models. The dependent variable in all models is the proportion of the available amount returned by the second mover. All models include country fixed effects as well as a constant (not shown). Shapley values refer to the variables of interest (conditional cooperation in Panel A and altruism in Panel B). Data are from a representative sample of respondents in Germany, Italy, Japan, Luxembourg, the UK and the USA. Cond. cooperation is the slope of the line of best fit when plotting the eleven conditional contribution decisions in Trustlab against the average contribution of others on which they are predicated, for the respondent in question.

Table F2: Shapley values for second-mover sending: Conditional cooperation and altruism

	(1)<br>0 CU	(2)<br>1 CU	(3)<br>2 CU	(4)<br>3 CU	(5)<br>4 CU	(6)<br>5 CU	(7)<br>6 CU	(8)<br>7 CU	(9)<br>8 CU	(10)<br>9 CU	(11)<br>10 CU
Panel A:											
Cond. cooperation	0.064	0.040	0.017	0.004	0.001	0.000	0.002	0.004	0.007	0.010	0.011
Country fixed-effects	0.009	0.010	0.009	0.005	0.005	0.003	0.005	0.004	0.004	0.003	0.003
Panel B:											
Altruism(std.)	0.118	0.130	0.122	0.115	0.104	0.100	0.091	0.084	0.079	0.077	0.076
Country fixed-effects	0.009	0.010	0.007	0.004	0.003	0.002	0.003	0.003	0.003	0.002	0.002

Note: Results show Shapley values corresponding to Table F1.

Table F3: Second-mover sending, conditional cooperation, and other correlates: additional specifications (first-mover sends 0, 1, 2 or 3)

VARIABLES	(1)	(2)	(3)	(4)	(5)
Conditional cooperation	-0.039***				-0.033***
	(0.003)				(0.003)
Altruism (standardised)		0.081***			0.078***
		(0.003)			(0.003)
Risk proclivity			0.008***		0.005***
			(0.002)		(0.001)
Female				0.003	0.011**
				(0.005)	(0.005)
Non-tertiary diploma				-0.004	0.000
				(0.007)	(0.007)
University				-0.017***	-0.005
				(0.006)	(0.005)
Age				0.001***	0.001***
				(0.000)	(0.000)
Respondent born in country				-0.007	-0.007
				(0.010)	(0.009)
Household size				0.002	0.000
				(0.002)	(0.002)
Rural				0.005	0.002
				(0.007)	(0.006)
Working				0.015***	0.008*
				(0.005)	(0.005)
Religion important				0.026***	0.012**
				(0.006)	(0.005)
Completely satisfied with life				0.036***	0.019**
				(0.009)	(0.008)
Observations	28,944	28,944	28,944	28,944	28,944
R-squared	0.035	0.119	0.013	0.021	0.146

Note: Estimates of OLS regression models with individual-level observations. Dependent variable: proportion of available CU (i.e. tripled amount received [if any] plus 10 CU endowment) that i sends to their first-mover when in the second-mover role in the trust game. Estimates use the set of observations of second-mover sending choices conditional on the amount sent by the first-mover being 0, 1, 2 or 3. Standard errors are clustered at the individual level. All models control for country fixed effects.

Table F4: Second-mover sending, conditional cooperation, and other correlates: additional specifications (first-mover sends 4, 5 or 6)

VARIABLES	(1)	(2)	(3)	(4)	(5)
Conditional cooperation	0.003				0.005**
	(0.003)				(0.003)
Altruism (standardised)		0.064***			0.063***
		(0.003)			(0.003)
Risk proclivity			0.005***		0.003**
			(0.001)		(0.001)
Female				-0.009*	-0.004
				(0.005)	(0.004)
Non-tertiary diploma				0.002	0.005
				(0.007)	(0.006)
University				0.003	0.009*
				(0.005)	(0.005)
Age				-0.000	-0.000
				(0.000)	(0.000)
Respondent born in country				-0.010	-0.011
				(0.009)	(0.008)
Household size				0.000	-0.001
				(0.002)	(0.002)
Rural				0.009	0.007
				(0.006)	(0.006)
Working				0.004	0.000
				(0.005)	(0.005)
Religion important				0.008	0.000
				(0.005)	(0.005)
Completely satisfied with life				0.018**	0.010
				(0.008)	(0.007)
Observations	21,708	21,708	21,708	21,708	21,708
R-squared	0.004	0.101	0.006	0.007	0.103

Note: Estimates of OLS regression models with individual-level observations. Dependent variable: proportion of available CU (i.e. tripled amount received [if any] plus 10 CU endowment) that i sends to their first-mover when in the second-mover role in the trust game. Estimates use the set of observations of second-mover sending choices conditional on the amount sent by the first-mover being 4, 5 or 6. Standard errors are clustered at the individual level. All models control for country fixed effects.

Table F5: Second-mover sending, conditional cooperation, and other correlates: additional specifications (first-mover sends 7, 8, 9 or 10)

VARIABLES	(1)	(2)	(3)	(4)	(5)
Conditional cooperation	0.019***				0.022***
	(0.003)				(0.003)
Altruism (standardised)		0.060***			0.060***
		(0.003)			(0.003)
Risk proclivity			0.005***		0.004***
			(0.002)		(0.001)
Female				-0.019***	-0.014***
				(0.005)	(0.005)
Non-tertiary diploma				0.002	0.003
				(0.007)	(0.007)
University				0.009	0.013**
				(0.006)	(0.005)
Age				-0.000***	-0.000**
				(0.000)	(0.000)
Respondent born in country				-0.009	-0.009
				(0.009)	(0.009)
Household size				0.000	-0.001
				(0.002)	(0.002)
Rural				0.007	0.006
				(0.007)	(0.006)
Working				-0.000	-0.004
				(0.005)	(0.005)
Religion important				0.001	-0.005
				(0.006)	(0.005)
Completely satisfied with life				0.011	0.006
				(0.008)	(0.008)
Observations	28,944	28,944	28,944	28,944	28,944
R-squared	0.011	0.081	0.005	0.007	0.096

Note: Estimates of OLS regression models with individual-level observations. Dependent variable: proportion of available CU (i.e. tripled amount received [if any] plus 10 CU endowment) that i sends to their first-mover when in the second-mover role in the trust game. Estimates use the set of observations of second-mover sending choices conditional on the amount sent by the first-mover being 7, 8, 9 or 10. Standard errors are clustered at the individual level. All models control for country fixed effects.

Table F6: Second-mover sending, conditional cooperation, and other correlates: additional specifications (pooled, including all first-mover sending amounts)

VARIABLES	(1)	(2)	(3)	(4)	(5)
Conditional cooperation	-0.006**				-0.003
	(0.003)				(0.002)
Altruism (standardised)		0.068***			0.068***
		(0.003)			(0.003)
Risk proclivity			0.006***		0.004***
			(0.001)		(0.001)
Female				-0.008*	-0.002
				(0.004)	(0.004)
Non-tertiary diploma				-0.000	0.003
				(0.006)	(0.006)
University				-0.002	0.005
				(0.005)	(0.005)
Age				0.000	0.000
				(0.000)	(0.000)
Respondent born in country				-0.009	-0.009
				(0.008)	(0.008)
Household size				0.001	-0.000
				(0.002)	(0.002)
Rural				0.007	0.005
				(0.006)	(0.006)
Working				0.007	0.002
				(0.005)	(0.004)
Religion important				0.012**	0.003
				(0.005)	(0.005)
Completely satisfied with life				0.022***	0.012*
				(0.008)	(0.007)
Observations	79,596	79,596	79,596	79,596	79,596
R-squared	0.005	0.092	0.007	0.007	0.094

Note: Estimates of OLS regression models with individual-level observations. Dependent variable: proportion of available CU (i.e. tripled amount received [if any] plus 10 CU endowment) that i sends to their first-mover when in the second-mover role in the trust game. Estimates use all second-mover sending choices. For each individual there are eleven observations (for the eleven possible sending amounts – i.e. 0 CU-10CU). Standard errors are clustered at the individual level. All models control for country fixed effects.

Figure F1: Trustworthiness and reciprocity

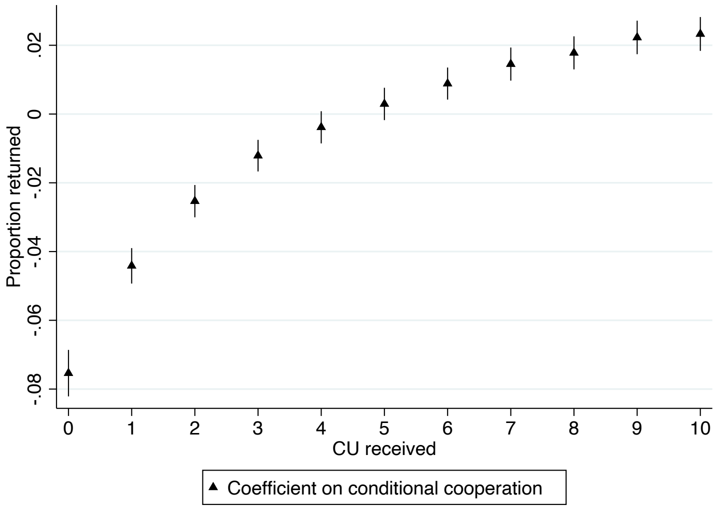

Note: The figure plots coefficients from OLS regressions, where the proportion of the available amount returned by second movers is regressed on conditional cooperation (based on the public goods game). Markers show regression coefficients and confidence intervals on conditional cooperation, corresponding to Panel A in Table F1. The first marker depicts results from Model 1 (0 CU sent), the second marker depicts Model 2 (1 CU sent) and so on.

Figure F2 plots the average proportion returned by trustees in the trust game on the amount received by trustors. The blue reference line shows how much a participant preferring an egalitarian split of resources would return if they were motivated only by their distributional preferences.[38] Overall, participants return larger amounts than participants who are solely motivated by reciprocity. For instance, participants who receive 0 CU from trustees nonetheless return 1.9 CU (19 %) on average - using their own baseline endowment. Whilst the proportion returned is an increasing function of the amount sent by the trustee, this rate of increase falls when larger amounts are received. When trustees send 5 CU or less, a one point increase in the amount sent is associated with a 3 percentage point increase in the proportion returned, compared to only a 0.9 percentage point increase when trustees send more than 5 CU. Table F7 regresses the proportion returned by the trustee against the standardised amount sent by the trustor. A one standard-deviation increase in the amount sent, is associated with a 6 percentage point increase in the proportion returned (or 0.24 SD).

Figure F2: Amount returned in the trust game based on the amount sent

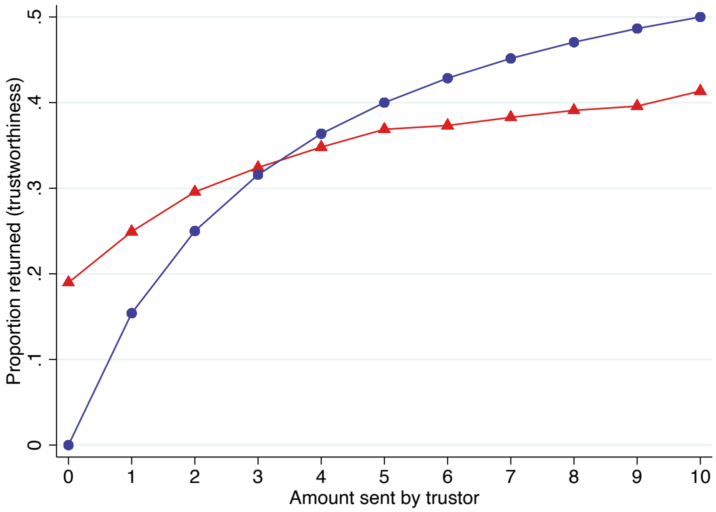

Note: The figure plots the proportion returned by the trustee in the trust game against the amount sent by the trustor (red triangles). The blue reference line indicates how much a participant preferring an equal split $(10 - x + y = 10 + 3x - y)$ would return if they were motivated only by their distribution preferences.

[38] That is, when $10 - x + y = 10 + 3x - y$.

Table F7: Proportion returned in the investment game based on the amount received (OLS regressions)

VARIABLES	(1)	(2)
Standarised amount sent by trustee	0.056***	0.056***
	(0.001)	(0.001)
Unconditional kindness (standardised)		0.067***
		(0.004)
Lower risk (7 or 10)		-0.013
		(0.008)
Low risk (6 or 12)		-0.012
		(0.008)
Medium risk (5 or 14)		0.001
		(0.009)
High risk (4 or 16)		0.007
		(0.011)
Highest risk (1 or 19)		0.011
		(0.011)
Female		0.002
		(0.006)
Non-tertiary diploma		0.001
		(0.007)
University		0.008
		(0.007)
Age		0.000
		(0.000)
Respondent born in country		-0.006
		(0.011)
Household size		0.000
		(0.002)
Rural		0.002
		(0.006)
Working		-0.001
		(0.006)
Religion important		-0.000
		(0.006)
Completely satisfied		0.008
		(0.008)
UK	-0.008	0.002
	(0.008)	(0.008)
Italy	0.011	0.024***
	(0.008)	(0.008)
USA	0.010	0.006
	(0.008)	(0.008)
Observations	51,799	51,788
R-squared	0.059	0.143

Note: Results are from OLS regression models. The dependent variable in all models is the proportion of the available amount returned by second movers. Respondents were asked which proportion they would return to the trustor for each amount received (0 CU - 10 CU), meaning that there are 11 observations for each respondent.

F.2 Trustworthiness, altruism, risk preferences and personal characteristics

Figure F3: Trustworthiness and altruism

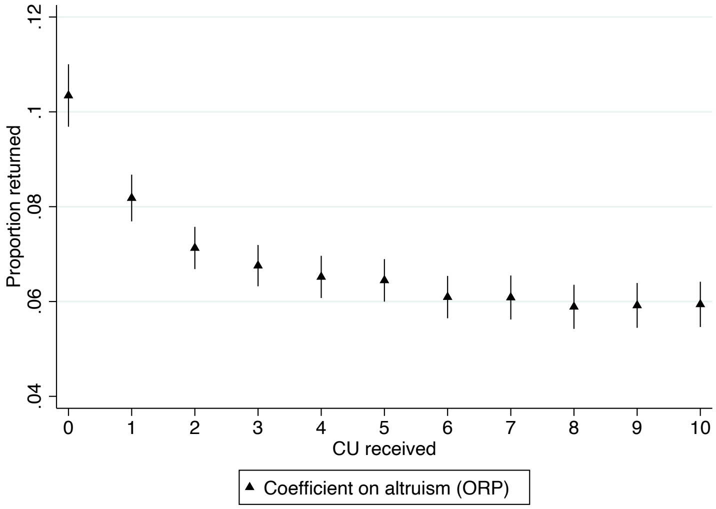

Note: The figure plots coefficients from OLS regressions, where the proportion of the available amount returned by second movers is regressed on altruism (i.e. the amount sent in the dictator game). Markers show regression coefficients and confidence intervals on altruism, corresponding to Panel B in Table F1. The first marker depicts results from Model 1 (0 CU sent), the second marker depicts Model 2 (1 CU sent) and so on.

Table F8: Personal characteristics and trustworthy behaviour (OLS regressions)

VARIABLES	(1)<br>0 CU	(2)<br>1 CU	(3)<br>2 CU	(4)<br>3 CU	(5)<br>4 CU	(6)<br>5 CU	(7)<br>6 CU	(8)<br>7 CU	(9)<br>8 CU	(10)<br>9 CU	(11)<br>10 CU
Female	0.011	0.004	-0.001	-0.003	-0.006	-0.009**	-0.012**	-0.015***	-0.017***	-0.021***	-0.022***
	(0.007)	(0.005)	(0.005)	(0.005)	(0.005)	(0.005)	(0.005)	(0.005)	(0.005)	(0.005)	(0.005)
Non-tertiary diploma	-0.015	-0.003	0.000	0.000	0.002	0.003	0.002	-0.001	0.001	0.001	0.008
	(0.010)	(0.008)	(0.007)	(0.007)	(0.007)	(0.007)	(0.007)	(0.007)	(0.007)	(0.007)	(0.007)
University	-0.036***	-0.018***	-0.008	-0.006	0.001	0.002	0.006	0.007	0.008	0.009	0.012**
	(0.008)	(0.006)	(0.006)	(0.006)	(0.006)	(0.006)	(0.006)	(0.006)	(0.006)	(0.006)	(0.006)
Age	0.003***	0.001***	0.001***	0.000*	-0.000	-0.000	-0.000	-0.000**	-0.000***	-0.001***	-0.001***
	(0.000)	(0.000)	(0.000)	(0.000)	(0.000)	(0.000)	(0.000)	(0.000)	(0.000)	(0.000)	(0.000)
Respondent born in country	-0.011	-0.004	-0.003	-0.009	-0.011	-0.012	-0.009	-0.013	-0.011	-0.005	-0.006
	(0.013)	(0.010)	(0.009)	(0.009)	(0.009)	(0.009)	(0.009)	(0.009)	(0.009)	(0.009)	(0.009)
Household size	0.004	0.002	0.002	0.001	0.000	-0.001	0.001	0.001	0.000	-0.000	0.000
	(0.003)	(0.002)	(0.002)	(0.002)	(0.002)	(0.002)	(0.002)	(0.002)	(0.002)	(0.002)	(0.002)
Rural	-0.005	0.007	0.008	0.009	0.007	0.011*	0.008	0.009	0.007	0.007	0.006
	(0.009)	(0.007)	(0.006)	(0.006)	(0.006)	(0.006)	(0.006)	(0.006)	(0.006)	(0.006)	(0.007)
Working	0.025***	0.017***	0.010*	0.008	0.006	0.004	0.002	-0.001	0.001	-0.000	-0.001
	(0.008)	(0.006)	(0.005)	(0.005)	(0.005)	(0.005)	(0.005)	(0.005)	(0.005)	(0.005)	(0.005)
Religion important	0.042***	0.026***	0.019***	0.016***	0.010*	0.007	0.006	0.001	0.001	0.000	0.000
	(0.008)	(0.006)	(0.005)	(0.005)	(0.005)	(0.005)	(0.005)	(0.006)	(0.006)	(0.006)	(0.006)
Completely satisfied with life	0.050***	0.038***	0.032***	0.024***	0.023***	0.018**	0.014*	0.015**	0.011	0.012	0.006
	(0.011)	(0.008)	(0.007)	(0.007)	(0.007)	(0.007)	(0.007)	(0.008)	(0.008)	(0.008)	(0.008)
Observations	7,236	7,236	7,236	7,236	7,236	7,236	7,236	7,236	7,236	7,236	7,236
R-squared	0.042	0.027	0.018	0.010	0.008	0.006	0.007	0.007	0.007	0.007	0.007
Country fixed-effects	✓	✓	✓	✓	✓	✓	✓	✓	✓	✓	✓

Note: Results are from OLS regression models. The dependent variable in all models is the proportion of money returned by the trustee in the investment game.

Table F9: Correlates of second-mover behaviour

	(1)<br>0 CU	(2)<br>1 CU	(3)<br>2 CU	(4)<br>3 CU	(5)<br>4 CU	(6)<br>5 CU	(7)<br>6 CU	(8)<br>7 CU	(9)<br>8 CU	(10)<br>9 CU	(11)<br>10 CU
Cond. cooperation	-0.066***	-0.038***	-0.021***	-0.008***	-0.001	0.006***	0.011***	0.017***	0.020***	0.024***	0.025***
	(0.003)	(0.002)	(0.002)	(0.002)	(0.002)	(0.002)	(0.002)	(0.002)	(0.002)	(0.002)	(0.002)
Altruism	0.099***	0.079***	0.070***	0.066***	0.065***	0.064***	0.061***	0.061***	0.059***	0.060***	0.060***
	(0.003)	(0.002)	(0.002)	(0.002)	(0.002)	(0.002)	(0.002)	(0.002)	(0.002)	(0.002)	(0.002)
Risk proclivity	0.006***	0.004***	0.004***	0.004***	0.003**	0.004***	0.003**	0.004***	0.004***	0.003**	0.004***
	(0.002)	(0.001)	(0.001)	(0.001)	(0.001)	(0.001)	(0.001)	(0.001)	(0.001)	(0.001)	(0.001)
Female	0.022***	0.013**	0.006	0.003	-0.000	-0.004	-0.007	-0.011**	-0.012**	-0.017***	-0.018***
	(0.006)	(0.005)	(0.005)	(0.004)	(0.005)	(0.005)	(0.005)	(0.005)	(0.005)	(0.005)	(0.005)
Other diploma	-0.008	0.002	0.004	0.003	0.005	0.005	0.004	0.001	0.002	0.002	0.009
	(0.009)	(0.007)	(0.006)	(0.006)	(0.006)	(0.006)	(0.006)	(0.007)	(0.007)	(0.007)	(0.007)
University	-0.018**	-0.005	0.002	0.002	0.008	0.007	0.011**	0.011*	0.012**	0.013**	0.015***
	(0.008)	(0.006)	(0.005)	(0.005)	(0.005)	(0.005)	(0.005)	(0.006)	(0.006)	(0.006)	(0.006)
Age	0.002***	0.001***	0.000**	0.000	-0.000	-0.000	-0.000	-0.000*	-0.000**	-0.000**	-0.000**
	(0.000)	(0.000)	(0.000)	(0.000)	(0.000)	(0.000)	(0.000)	(0.000)	(0.000)	(0.000)	(0.000)
Born in country	-0.011	-0.004	-0.003	-0.010	-0.011	-0.012	-0.009	-0.013	-0.011	-0.006	-0.006
	(0.012)	(0.009)	(0.008)	(0.008)	(0.008)	(0.009)	(0.008)	(0.009)	(0.009)	(0.009)	(0.009)
Household size	0.002	-0.000	0.000	-0.001	-0.001	-0.002	-0.000	-0.000	-0.001	-0.001	-0.001
	(0.003)	(0.002)	(0.002)	(0.002)	(0.002)	(0.002)	(0.002)	(0.002)	(0.002)	(0.002)	(0.002)
Rural	-0.008	0.004	0.006	0.007	0.005	0.010*	0.007	0.007	0.006	0.006	0.005
	(0.008)	(0.006)	(0.006)	(0.006)	(0.006)	(0.006)	(0.006)	(0.006)	(0.006)	(0.006)	(0.006)
Working	0.015**	0.010*	0.004	0.003	0.002	0.000	-0.002	-0.004	-0.002	-0.003	-0.004
	(0.007)	(0.005)	(0.005)	(0.005)	(0.005)	(0.005)	(0.005)	(0.005)	(0.005)	(0.005)	(0.005)
Religion important	0.022***	0.011**	0.007	0.006	0.002	-0.000	-0.001	-0.005	-0.005	-0.005	-0.004
	(0.007)	(0.006)	(0.005)	(0.005)	(0.005)	(0.005)	(0.005)	(0.005)	(0.005)	(0.005)	(0.005)
Satisfied with life	0.024**	0.020***	0.018**	0.013*	0.013*	0.010	0.007	0.010	0.006	0.008	0.002
	(0.010)	(0.008)	(0.007)	(0.007)	(0.007)	(0.007)	(0.007)	(0.007)	(0.007)	(0.007)	(0.007)
Observations	7,236	7,236	7,236	7,236	7,236	7,236	7,236	7,236	7,236	7,236	7,236
R-squared	0.197	0.177	0.144	0.123	0.109	0.105	0.100	0.097	0.095	0.097	0.097

Note: Results are from OLS regression models. The dependent variable in all models is the proportion of the available amount returned by the second mover. All models include country fixed effects as well as a constant (not shown). Data are from a representative sample of respondents in Germany, Italy, Japan, Luxembourg, the UK and the USA. Cond. cooperation stands for conditional cooperation.

F.3 Preferences for redistribution

Table F10: Proportion returned and preferences for redistribution

VARIABLES	(1)<br>0 CU	(2)<br>1 CU	(3)<br>2 CU	(4)<br>3 CU	(5)<br>4 CU	(6)<br>5 CU	(7)<br>6 CU	(8)<br>7 CU	(9)<br>8 CU	(10)<br>9 CU	(11)<br>10 CU
Tax burden top 1%	0.034***	0.013*	0.004	0.003	0.001	-0.002	-0.007	-0.010	-0.007	-0.012*	-0.014*
	(0.010)	(0.008)	(0.007)	(0.007)	(0.007)	(0.007)	(0.007)	(0.007)	(0.007)	(0.007)	(0.007)
Tax burden next 9%	0.072***	0.031*	0.010	0.009	-0.002	-0.008	-0.011	-0.021	-0.016	-0.024	-0.026
	(0.024)	(0.018)	(0.016)	(0.016)	(0.016)	(0.016)	(0.016)	(0.017)	(0.017)	(0.017)	(0.017)
Tax burden next 40%	0.073***	0.034**	0.013	0.011	0.005	-0.002	-0.007	-0.016	-0.011	-0.020	-0.022
	(0.022)	(0.017)	(0.015)	(0.015)	(0.015)	(0.015)	(0.015)	(0.015)	(0.015)	(0.016)	(0.016)
Tax burden bottom 50%	0.096***	0.046***	0.021	0.015	0.004	-0.005	-0.013	-0.023	-0.018	-0.030*	-0.031*
	(0.023)	(0.017)	(0.016)	(0.015)	(0.015)	(0.016)	(0.015)	(0.016)	(0.016)	(0.016)	(0.016)
Observations	7,236	7,236	7,236	7,236	7,236	7,236	7,236	7,236	7,236	7,236	7,236
R-squared	0.020	0.018	0.013	0.007	0.006	0.004	0.005	0.004	0.004	0.004	0.004
Country fixed-effects	✓	✓	✓	✓	✓	✓	✓	✓	✓	✓	✓

Note: Results are from OLS regression models. The dependent variable in all models is the proportion of the available amount returned by second movers. Data are from a representative sample of respondents in Germany, Italy, Japan, Luxembourg, the UK and the USA.

Table F11: Proportion returned and preferences for redistribution, including controls

VARIABLES	(1)<br>0 CU	(2)<br>1 CU	(3)<br>2 CU	(4)<br>3 CU	(5)<br>4 CU	(6)<br>5 CU	(7)<br>6 CU	(8)<br>7 CU	(9)<br>8 CU	(10)<br>9 CU	(11)<br>10 CU
Tax burden top 1%	0.021**	0.005	-0.002	-0.002	-0.003	-0.006	-0.010	-0.012*	-0.009	-0.015**	-0.016**
	(0.010)	(0.007)	(0.007)	(0.007)	(0.007)	(0.007)	(0.007)	(0.007)	(0.007)	(0.007)	(0.007)
Tax burden next 9%	0.049**	0.015	-0.002	-0.001	-0.010	-0.016	-0.018	-0.027*	-0.020	-0.028*	-0.030*
	(0.022)	(0.017)	(0.015)	(0.015)	(0.015)	(0.015)	(0.015)	(0.016)	(0.016)	(0.016)	(0.016)
Tax burden next 40%	0.048**	0.016	-0.001	-0.001	-0.004	-0.011	-0.014	-0.023	-0.017	-0.025*	-0.028*
	(0.020)	(0.015)	(0.014)	(0.014)	(0.014)	(0.014)	(0.014)	(0.015)	(0.015)	(0.015)	(0.015)
Tax burden bottom 50%	0.061***	0.022	0.002	0.000	-0.008	-0.016	-0.022	-0.030**	-0.025	-0.035**	-0.037**
	(0.021)	(0.016)	(0.015)	(0.014)	(0.015)	(0.015)	(0.015)	(0.015)	(0.015)	(0.015)	(0.016)
Conditional cooperation	-0.069***	-0.040***	-0.022***	-0.009***	-0.001	0.006**	0.011***	0.017***	0.020***	0.025***	0.026***
	(0.003)	(0.002)	(0.002)	(0.002)	(0.002)	(0.002)	(0.002)	(0.002)	(0.002)	(0.002)	(0.002)
Altruism ORP (standardised)	0.099***	0.079***	0.070***	0.067***	0.065***	0.064***	0.061***	0.061***	0.060***	0.060***	0.060***
	(0.003)	(0.002)	(0.002)	(0.002)	(0.002)	(0.002)	(0.002)	(0.002)	(0.002)	(0.002)	(0.002)
Risk proclivity	0.006***	0.004***	0.004***	0.004***	0.003**	0.004***	0.003**	0.004***	0.004***	0.004***	0.004***
	(0.002)	(0.001)	(0.001)	(0.001)	(0.001)	(0.001)	(0.001)	(0.001)	(0.001)	(0.001)	(0.001)
Observations	7,236	7,236	7,236	7,236	7,236	7,236	7,236	7,236	7,236	7,236	7,236
R-squared	0.185	0.173	0.143	0.122	0.108	0.104	0.099	0.096	0.093	0.095	0.095
Country fixed-effects	✓	✓	✓	✓	✓	✓	✓	✓	✓	✓	✓

Note: Results are from OLS regression models. The dependent variable in all models is the proportion of the available amount returned by second movers. Data are from a representative sample of respondents in Germany, Italy, Japan, Luxembourg, the UK and the USA.

F.4 Conformity

Table F12: Proportion returned and conformity

VARIABLES	(1)<br>0 CU	(2)<br>1 CU	(3)<br>2 CU	(4)<br>3 CU	(5)<br>4 CU	(6)<br>5 CU	(7)<br>6 CU	(8)<br>7 CU	(9)<br>8 CU	(10)<br>9 CU	(11)<br>10 CU
Non-conformity	-0.065*	-0.043	-0.025	-0.002	-0.012	-0.006	-0.009	-0.014	-0.010	-0.007	-0.004
	(0.034)	(0.027)	(0.024)	(0.023)	(0.024)	(0.024)	(0.024)	(0.025)	(0.024)	(0.025)	(0.025)
Constant	0.212***	0.273***	0.306***	0.317***	0.350***	0.369***	0.374***	0.391***	0.393***	0.393***	0.411***
	(0.022)	(0.018)	(0.016)	(0.016)	(0.016)	(0.016)	(0.016)	(0.016)	(0.016)	(0.017)	(0.017)
Observations	1,034	1,034	1,034	1,034	1,034	1,034	1,034	1,034	1,034	1,034	1,034
R-squared	0.004	0.002	0.001	0.000	0.000	0.000	0.000	0.000	0.000	0.000	0.000

Note: Results are from OLS regression models. The dependent variable in all models is the proportion of the available amount returned by second movers. Data are from a representative sample of respondents in the UK.

Table F13: Proportion returned and conformity (including controls)

VARIABLES	(1)<br>0 CU	(2)<br>1 CU	(3)<br>2 CU	(4)<br>3 CU	(5)<br>4 CU	(6)<br>5 CU	(7)<br>6 CU	(8)<br>7 CU	(9)<br>8 CU	(10)<br>9 CU	(11)<br>10 CU
Non-conformity	-0.062**	-0.047*	-0.030	-0.011	-0.021	-0.017	-0.020	-0.027	-0.022	-0.022	-0.020
	(0.031)	(0.025)	(0.023)	(0.022)	(0.023)	(0.023)	(0.023)	(0.023)	(0.023)	(0.024)	(0.024)
Conditional cooperation	-0.074***	-0.032***	-0.014**	0.003	0.008	0.016**	0.023***	0.030***	0.033***	0.039***	0.039***
	(0.009)	(0.007)	(0.006)	(0.006)	(0.006)	(0.006)	(0.006)	(0.007)	(0.007)	(0.007)	(0.007)
Altruism ORP (standardised)	0.077***	0.071***	0.059***	0.060***	0.057***	0.062***	0.054***	0.058***	0.054***	0.060***	0.064***
	(0.008)	(0.007)	(0.006)	(0.006)	(0.006)	(0.006)	(0.006)	(0.006)	(0.006)	(0.006)	(0.007)
Risk proclivity	0.016***	0.013***	0.011***	0.009**	0.011***	0.010***	0.008**	0.009**	0.010***	0.006	0.009**
	(0.005)	(0.004)	(0.004)	(0.004)	(0.004)	(0.004)	(0.004)	(0.004)	(0.004)	(0.004)	(0.004)
Constant	0.173***	0.243***	0.279***	0.299***	0.325***	0.347***	0.357***	0.373***	0.370***	0.383***	0.393***
	(0.025)	(0.020)	(0.018)	(0.018)	(0.018)	(0.018)	(0.018)	(0.019)	(0.019)	(0.019)	(0.019)
Observations	1,034	1,034	1,034	1,034	1,034	1,034	1,034	1,034	1,034	1,034	1,034
R-squared	0.155	0.131	0.102	0.094	0.087	0.100	0.081	0.093	0.088	0.103	0.111
Country fixed-effects	✓	✓	✓	✓	✓	✓	✓	✓	✓	✓	✓

Note: Results are from OLS regression models. The dependent variable in all models is the proportion of the available amount returned by second movers. Data are from a representative sample of respondents in the UK.

F.5 Religion

Table F14: Proportion returned and religious affiliation (Catholics)

VARIABLES	(1)<br>0 CU	(2)<br>1 CU	(3)<br>2 CU	(4)<br>3 CU	(5)<br>4 CU	(6)<br>5 CU	(7)<br>6 CU	(8)<br>7 CU	(9)<br>8 CU	(10)<br>9 CU	(11)<br>10 CU
Catholic	0.031**	0.013	0.008	0.010	0.006	0.001	-0.004	-0.004	-0.005	-0.005	-0.012
	(0.013)	(0.010)	(0.009)	(0.009)	(0.009)	(0.009)	(0.009)	(0.009)	(0.009)	(0.009)	(0.009)
UK	-0.016	0.001	-0.006	-0.010	-0.010	-0.007	-0.013	-0.009	-0.012	-0.013	-0.012
	(0.013)	(0.010)	(0.009)	(0.009)	(0.009)	(0.009)	(0.009)	(0.009)	(0.009)	(0.009)	(0.009)
Italy	0.003	0.017	0.013	0.008	0.006	0.011	0.014	0.010	0.012	0.013	0.014
	(0.015)	(0.011)	(0.010)	(0.010)	(0.010)	(0.010)	(0.010)	(0.010)	(0.010)	(0.010)	(0.011)
Japan	-0.034***	-0.030***	-0.029***	-0.019**	-0.022***	-0.017**	-0.026***	-0.025***	-0.022***	-0.016*	-0.020**
	(0.012)	(0.009)	(0.008)	(0.008)	(0.008)	(0.008)	(0.008)	(0.008)	(0.008)	(0.008)	(0.008)
Constant	0.185***	0.245***	0.296***	0.325***	0.352***	0.372***	0.382***	0.392***	0.400***	0.402***	0.422***
	(0.010)	(0.007)	(0.007)	(0.006)	(0.007)	(0.007)	(0.007)	(0.007)	(0.007)	(0.007)	(0.007)
Observations	5,184	5,184	5,184	5,184	5,184	5,184	5,184	5,184	5,184	5,184	5,184
R-squared	0.008	0.010	0.008	0.005	0.004	0.003	0.005	0.004	0.003	0.002	0.003

Note: Results are from OLS regression models. The dependent variable in all models is the proportion of the available amount returned by second movers. Data are from a representative sample of respondents in Germany, Italy, Japan and the UK.

Table F15: Proportion returned and religious affiliation (Catholics), including controls

VARIABLES	(1)<br>0 CU	(2)<br>1 CU	(3)<br>2 CU	(4)<br>3 CU	(5)<br>4 CU	(6)<br>5 CU	(7)<br>6 CU	(8)<br>7 CU	(9)<br>8 CU	(10)<br>9 CU	(11)<br>10 CU
Catholic	0.031**	0.014	0.010	0.013	0.010	0.005	0.001	0.002	0.001	0.001	-0.005
	(0.012)	(0.009)	(0.008)	(0.008)	(0.008)	(0.008)	(0.008)	(0.009)	(0.009)	(0.009)	(0.009)
Conditional cooperation	-0.067***	-0.037***	-0.021***	-0.008***	-0.000	0.006**	0.013***	0.018***	0.022***	0.026***	0.027***
	(0.004)	(0.003)	(0.003)	(0.003)	(0.003)	(0.003)	(0.003)	(0.003)	(0.003)	(0.003)	(0.003)
Altruism ORP (standardised)	0.098***	0.078***	0.070***	0.068***	0.066***	0.066***	0.063***	0.064***	0.061***	0.062***	0.062***
	(0.004)	(0.003)	(0.003)	(0.003)	(0.003)	(0.003)	(0.003)	(0.003)	(0.003)	(0.003)	(0.003)
Risk proclivity	0.005**	0.005***	0.005***	0.004***	0.004**	0.004**	0.003**	0.004***	0.005***	0.004**	0.005***
	(0.002)	(0.002)	(0.002)	(0.002)	(0.002)	(0.002)	(0.002)	(0.002)	(0.002)	(0.002)	(0.002)
Constant	0.151***	0.216***	0.270***	0.301***	0.332***	0.351***	0.364***	0.372***	0.379***	0.385***	0.400***
	(0.011)	(0.008)	(0.008)	(0.007)	(0.008)	(0.008)	(0.008)	(0.008)	(0.008)	(0.008)	(0.008)
Observations	5,184	5,184	5,184	5,184	5,184	5,184	5,184	5,184	5,184	5,184	5,184
R-squared	0.170	0.163	0.143	0.128	0.114	0.111	0.108	0.107	0.102	0.105	0.104
Country fixed-effects	✓	✓	✓	✓	✓	✓	✓	✓	✓	✓	✓

Note: Results are from OLS regression models. The dependent variable in all models is the proportion of the available amount returned by second movers. Data are from a representative sample of respondents in Germany, Italy, Japan and the UK.

Table F16: Proportion returned and religious affiliation (Protestants)

VARIABLES	(1)<br>0 CU	(2)<br>1 CU	(3)<br>2 CU	(4)<br>3 CU	(5)<br>4 CU	(6)<br>5 CU	(7)<br>6 CU	(8)<br>7 CU	(9)<br>8 CU	(10)<br>9 CU	(11)<br>10 CU
Protestant	0.014	0.008	0.002	0.002	-0.001	-0.003	-0.004	-0.005	-0.008	-0.012	-0.009
	(0.015)	(0.011)	(0.010)	(0.010)	(0.010)	(0.010)	(0.010)	(0.011)	(0.011)	(0.011)	(0.011)
UK	-0.019	-0.000	-0.007	-0.011	-0.010	-0.007	-0.013	-0.009	-0.012	-0.013	-0.012
	(0.013)	(0.010)	(0.009)	(0.009)	(0.009)	(0.009)	(0.009)	(0.009)	(0.009)	(0.009)	(0.009)
Italy	0.022	0.026**	0.017*	0.013	0.009	0.010	0.011	0.006	0.007	0.007	0.006
	(0.014)	(0.010)	(0.009)	(0.009)	(0.009)	(0.009)	(0.009)	(0.010)	(0.010)	(0.010)	(0.010)
Japan	-0.038***	-0.031***	-0.030***	-0.021***	-0.024***	-0.018**	-0.026***	-0.026***	-0.023***	-0.018**	-0.020**
	(0.012)	(0.009)	(0.008)	(0.008)	(0.008)	(0.008)	(0.008)	(0.008)	(0.008)	(0.008)	(0.009)
Constant	0.189***	0.246***	0.297***	0.326***	0.353***	0.373***	0.382***	0.392***	0.400***	0.404***	0.422***
	(0.010)	(0.008)	(0.007)	(0.007)	(0.007)	(0.007)	(0.007)	(0.007)	(0.007)	(0.007)	(0.007)
Observations	5,184	5,184	5,184	5,184	5,184	5,184	5,184	5,184	5,184	5,184	5,184
R-squared	0.007	0.010	0.008	0.004	0.004	0.003	0.005	0.004	0.003	0.002	0.002

Note: Results are from OLS regression models. The dependent variable in all models is the proportion of the available amount returned by second movers. Data are from a representative sample of respondents in Germany, Italy, Japan and the UK.

Table F17: Proportion returned and religious affiliation (Protestants), including controls

VARIABLES	(1)<br>0 CU	(2)<br>1 CU	(3)<br>2 CU	(4)<br>3 CU	(5)<br>4 CU	(6)<br>5 CU	(7)<br>6 CU	(8)<br>7 CU	(9)<br>8 CU	(10)<br>9 CU	(11)<br>10 CU
Protestant	0.004	0.001	-0.003	-0.002	-0.004	-0.006	-0.006	-0.007	-0.009	-0.013	-0.010
	(0.014)	(0.010)	(0.010)	(0.009)	(0.010)	(0.010)	(0.010)	(0.010)	(0.010)	(0.010)	(0.010)
Conditional cooperation	-0.067***	-0.038***	-0.021***	-0.008***	-0.000	0.006**	0.013***	0.018***	0.021***	0.026***	0.027***
	(0.004)	(0.003)	(0.003)	(0.003)	(0.003)	(0.003)	(0.003)	(0.003)	(0.003)	(0.003)	(0.003)
Altruism ORP (standardised)	0.097***	0.078***	0.070***	0.067***	0.066***	0.066***	0.063***	0.064***	0.061***	0.062***	0.062***
	(0.004)	(0.003)	(0.003)	(0.003)	(0.003)	(0.003)	(0.003)	(0.003)	(0.003)	(0.003)	(0.003)
Risk proclivity	0.005**	0.005***	0.005***	0.004***	0.004**	0.004**	0.003**	0.004***	0.005***	0.004**	0.005***
	(0.002)	(0.002)	(0.002)	(0.002)	(0.002)	(0.002)	(0.002)	(0.002)	(0.002)	(0.002)	(0.002)
Constant	0.156***	0.219***	0.273***	0.305***	0.335***	0.354***	0.366***	0.374***	0.382***	0.389***	0.402***
	(0.011)	(0.009)	(0.008)	(0.008)	(0.008)	(0.008)	(0.008)	(0.008)	(0.008)	(0.008)	(0.008)
Observations	5,184	5,184	5,184	5,184	5,184	5,184	5,184	5,184	5,184	5,184	5,184
R-squared	0.169	0.162	0.143	0.127	0.113	0.111	0.108	0.107	0.102	0.106	0.105
Country fixed-effects	✓	✓	✓	✓	✓	✓	✓	✓	✓	✓	✓

Note: Results are from OLS regression models. The dependent variable in all models is the proportion of the available amount returned by second movers. Data are from a representative sample of respondents in Germany, Italy, Japan and the UK.

Table F18: Proportion returned and religious affiliation (no religion)

VARIABLES	(1)<br>0 CU	(2)<br>1 CU	(3)<br>2 CU	(4)<br>3 CU	(5)<br>4 CU	(6)<br>5 CU	(7)<br>6 CU	(8)<br>7 CU	(9)<br>8 CU	(10)<br>9 CU	(11)<br>10 CU
No religion	-0.034***	-0.022***	-0.015***	-0.017***	-0.012**	-0.009	-0.004	-0.001	0.001	0.002	0.005
	(0.009)	(0.006)	(0.006)	(0.006)	(0.006)	(0.006)	(0.006)	(0.006)	(0.006)	(0.006)	(0.006)
UK	-0.016	0.002	-0.005	-0.009	-0.009	-0.006	-0.012	-0.008	-0.012	-0.013	-0.012
	(0.013)	(0.010)	(0.009)	(0.009)	(0.009)	(0.009)	(0.009)	(0.009)	(0.009)	(0.009)	(0.009)
Italy	0.011	0.019*	0.013	0.009	0.006	0.009	0.011	0.008	0.009	0.010	0.009
	(0.013)	(0.010)	(0.009)	(0.009)	(0.009)	(0.009)	(0.009)	(0.009)	(0.009)	(0.009)	(0.009)
Japan	-0.035***	-0.029***	-0.027***	-0.018**	-0.021***	-0.015*	-0.024***	-0.024***	-0.021***	-0.015*	-0.018**
	(0.011)	(0.009)	(0.008)	(0.008)	(0.008)	(0.008)	(0.008)	(0.008)	(0.008)	(0.008)	(0.008)
Constant	0.207***	0.257***	0.304***	0.334***	0.358***	0.376***	0.383***	0.392***	0.398***	0.400***	0.417***
	(0.010)	(0.007)	(0.007)	(0.007)	(0.007)	(0.007)	(0.007)	(0.007)	(0.007)	(0.007)	(0.007)
Observations	5,184	5,184	5,184	5,184	5,184	5,184	5,184	5,184	5,184	5,184	5,184
R-squared	0.009	0.012	0.010	0.006	0.005	0.003	0.005	0.004	0.003	0.002	0.002

Note: Results are from OLS regression models. The dependent variable in all models is the proportion of the available amount returned by second movers. Data are from a representative sample of respondents in Germany, Italy, Japan and the UK.

Table F19: Proportion returned and religious affiliation (no religion), including controls

VARIABLES	(1)<br>0 CU	(2)<br>1 CU	(3)<br>2 CU	(4)<br>3 CU	(5)<br>4 CU	(6)<br>5 CU	(7)<br>6 CU	(8)<br>7 CU	(9)<br>8 CU	(10)<br>9 CU	(11)<br>10 CU
No religion	-0.023***	-0.015**	-0.010*	-0.013**	-0.009*	-0.007	-0.003	-0.000	0.001	0.003	0.006
	(0.008)	(0.006)	(0.005)	(0.005)	(0.005)	(0.006)	(0.005)	(0.006)	(0.006)	(0.006)	(0.006)
Conditional cooperation	-0.067***	-0.037***	-0.021***	-0.008***	-0.000	0.006**	0.013***	0.018***	0.021***	0.026***	0.027***
	(0.004)	(0.003)	(0.003)	(0.003)	(0.003)	(0.003)	(0.003)	(0.003)	(0.003)	(0.003)	(0.003)
Altruism ORP (standardised)	0.097***	0.078***	0.070***	0.067***	0.066***	0.066***	0.063***	0.064***	0.061***	0.062***	0.062***
	(0.004)	(0.003)	(0.003)	(0.003)	(0.003)	(0.003)	(0.003)	(0.003)	(0.003)	(0.003)	(0.003)
Risk proclivity	0.005**	0.005***	0.005***	0.004***	0.004**	0.004**	0.003**	0.004***	0.005***	0.004**	0.005***
	(0.002)	(0.002)	(0.002)	(0.002)	(0.002)	(0.002)	(0.002)	(0.002)	(0.002)	(0.002)	(0.002)
Constant	0.167***	0.225***	0.276***	0.309***	0.338***	0.355***	0.365***	0.372***	0.378***	0.384***	0.397***
	(0.011)	(0.008)	(0.008)	(0.008)	(0.008)	(0.008)	(0.008)	(0.008)	(0.008)	(0.008)	(0.008)
Observations	5,184	5,184	5,184	5,184	5,184	5,184	5,184	5,184	5,184	5,184	5,184
R-squared	0.170	0.163	0.144	0.128	0.114	0.112	0.108	0.107	0.102	0.105	0.105
Country fixed-effects	✓	✓	✓	✓	✓	✓	✓	✓	✓	✓	✓

Note: Results are from OLS regression models. The dependent variable in all models is the proportion of the available amount returned by second movers. Data are from a representative sample of respondents in Germany, Italy, Japan and the UK.

F.6 Personality traits

Table F20: Proportion returned and personality traits (agreeableness)

VARIABLES	(1)<br>0 CU	(2)<br>1 CU	(3)<br>2 CU	(4)<br>3 CU	(5)<br>4 CU	(6)<br>5 CU	(7)<br>6 CU	(8)<br>7 CU	(9)<br>8 CU	(10)<br>9 CU	(11)<br>10 CU
Agreeableness	0.002	0.000	0.001	0.001	0.000	0.001	0.000	0.000	0.000	0.001	0.001
	(0.003)	(0.002)	(0.002)	(0.002)	(0.002)	(0.002)	(0.002)	(0.002)	(0.002)	(0.002)	(0.002)
Italy	0.039***	0.025**	0.024***	0.024***	0.019**	0.018**	0.025***	0.016*	0.020**	0.023**	0.019**
	(0.013)	(0.010)	(0.009)	(0.009)	(0.009)	(0.009)	(0.009)	(0.009)	(0.009)	(0.009)	(0.009)
Japan	-0.020*	-0.032***	-0.022***	-0.009	-0.013*	-0.010	-0.012	-0.016**	-0.009	-0.002	-0.005
	(0.011)	(0.009)	(0.008)	(0.008)	(0.008)	(0.008)	(0.008)	(0.008)	(0.008)	(0.008)	(0.008)
Constant	0.166***	0.246***	0.287***	0.313***	0.342***	0.363***	0.368***	0.382***	0.386***	0.386***	0.406***
	(0.013)	(0.010)	(0.009)	(0.009)	(0.009)	(0.009)	(0.009)	(0.009)	(0.009)	(0.010)	(0.010)
Observations	4,186	4,186	4,186	4,186	4,186	4,186	4,186	4,186	4,186	4,186	4,186
R-squared	0.007	0.011	0.009	0.005	0.004	0.003	0.006	0.004	0.003	0.002	0.002

Note: Results are from OLS regression models. The dependent variable in all models is the proportion of the available amount returned by second movers. Data are from a representative sample of respondents in Italy, Japan and the UK.

Table F21: Proportion returned and personality traits (agreeableness), including controls

VARIABLES	(1)<br>0 CU	(2)<br>1 CU	(3)<br>2 CU	(4)<br>3 CU	(5)<br>4 CU	(6)<br>5 CU	(7)<br>6 CU	(8)<br>7 CU	(9)<br>8 CU	(10)<br>9 CU	(11)<br>10 CU
Agreeableness	0.002	-0.000	0.001	0.000	-0.000	0.000	-0.000	-0.000	-0.000	0.000	0.000
	(0.003)	(0.002)	(0.002)	(0.002)	(0.002)	(0.002)	(0.002)	(0.002)	(0.002)	(0.002)	(0.002)
Conditional cooperation	-0.061***	-0.031***	-0.013***	-0.002	0.006*	0.012***	0.018***	0.024***	0.027***	0.032***	0.032***
	(0.004)	(0.003)	(0.003)	(0.003)	(0.003)	(0.003)	(0.003)	(0.003)	(0.003)	(0.003)	(0.003)
Altruism ORP (standardised)	0.099***	0.079***	0.070***	0.067***	0.066***	0.065***	0.063***	0.063***	0.061***	0.063***	0.062***
	(0.004)	(0.003)	(0.003)	(0.003)	(0.003)	(0.003)	(0.003)	(0.003)	(0.003)	(0.003)	(0.003)
Risk proclivity	0.005*	0.006***	0.005***	0.005***	0.005***	0.005***	0.004**	0.005***	0.006***	0.005***	0.006***
	(0.002)	(0.002)	(0.002)	(0.002)	(0.002)	(0.002)	(0.002)	(0.002)	(0.002)	(0.002)	(0.002)
Constant	0.158***	0.234***	0.275***	0.301***	0.330***	0.351***	0.357***	0.368***	0.371***	0.373***	0.389***
	(0.014)	(0.011)	(0.010)	(0.010)	(0.010)	(0.010)	(0.010)	(0.010)	(0.010)	(0.010)	(0.010)
Observations	4,186	4,186	4,186	4,186	4,186	4,186	4,186	4,186	4,186	4,186	4,186
R-squared	0.166	0.160	0.143	0.129	0.119	0.117	0.117	0.114	0.112	0.117	0.114
Country fixed-effects	✓	✓	✓	✓	✓	✓	✓	✓	✓	✓	✓

Note: Results are from OLS regression models. The dependent variable in all models is the proportion of the available amount returned by second movers. Data are from a representative sample of respondents in Italy, Japan and the UK.

Table F22: Proportion returned and personality traits (conscientiousness)

VARIABLES	(1)<br>0 CU	(2)<br>1 CU	(3)<br>2 CU	(4)<br>3 CU	(5)<br>4 CU	(6)<br>5 CU	(7)<br>6 CU	(8)<br>7 CU	(9)<br>8 CU	(10)<br>9 CU	(11)<br>10 CU
Conscientiousness	0.003	0.002	0.001	0.001	0.000	-0.001	-0.000	-0.001	-0.001	-0.001	-0.002
	(0.003)	(0.002)	(0.002)	(0.002)	(0.002)	(0.002)	(0.002)	(0.002)	(0.002)	(0.002)	(0.002)
Italy	0.039***	0.025**	0.024***	0.024***	0.019**	0.018**	0.025***	0.016*	0.020**	0.022**	0.019**
	(0.013)	(0.010)	(0.009)	(0.009)	(0.009)	(0.009)	(0.009)	(0.009)	(0.009)	(0.009)	(0.009)
Japan	-0.015	-0.028***	-0.021**	-0.008	-0.013	-0.011	-0.013	-0.017**	-0.011	-0.004	-0.010
	(0.012)	(0.009)	(0.008)	(0.008)	(0.008)	(0.008)	(0.008)	(0.009)	(0.009)	(0.009)	(0.009)
Constant	0.153***	0.235***	0.283***	0.308***	0.341***	0.369***	0.371***	0.387***	0.391***	0.395***	0.420***
	(0.020)	(0.015)	(0.014)	(0.013)	(0.014)	(0.014)	(0.014)	(0.014)	(0.014)	(0.014)	(0.014)
Observations	4,186	4,186	4,186	4,186	4,186	4,186	4,186	4,186	4,186	4,186	4,186
R-squared	0.007	0.012	0.009	0.005	0.004	0.003	0.006	0.004	0.003	0.003	0.003

Note: Results are from OLS regression models. The dependent variable in all models is the proportion of the available amount returned by second movers. Data are from a representative sample of respondents in Italy, Japan and the UK.

Table F23: Proportion returned and personality traits (conscientiousness), including controls

VARIABLES	(1)<br>0 CU	(2)<br>1 CU	(3)<br>2 CU	(4)<br>3 CU	(5)<br>4 CU	(6)<br>5 CU	(7)<br>6 CU	(8)<br>7 CU	(9)<br>8 CU	(10)<br>9 CU	(11)<br>10 CU
Conscientiousness	0.003	0.002	0.001	0.001	0.000	-0.001	-0.001	-0.001	-0.001	-0.001	-0.002
	(0.003)	(0.002)	(0.002)	(0.002)	(0.002)	(0.002)	(0.002)	(0.002)	(0.002)	(0.002)	(0.002)
Conditional cooperation	-0.061***	-0.031***	-0.013***	-0.002	0.006*	0.012***	0.018***	0.024***	0.027***	0.032***	0.032***
	(0.004)	(0.003)	(0.003)	(0.003)	(0.003)	(0.003)	(0.003)	(0.003)	(0.003)	(0.003)	(0.003)
Altruism ORP (standardised)	0.099***	0.079***	0.070***	0.067***	0.066***	0.065***	0.063***	0.063***	0.061***	0.063***	0.062***
	(0.004)	(0.003)	(0.003)	(0.003)	(0.003)	(0.003)	(0.003)	(0.003)	(0.003)	(0.003)	(0.003)
Risk proclivity	0.005*	0.006***	0.005***	0.005***	0.005***	0.005***	0.004**	0.005***	0.006***	0.005***	0.006***
	(0.002)	(0.002)	(0.002)	(0.002)	(0.002)	(0.002)	(0.002)	(0.002)	(0.002)	(0.002)	(0.002)
Constant	0.146***	0.223***	0.271***	0.296***	0.329***	0.356***	0.359***	0.373***	0.376***	0.382***	0.403***
	(0.019)	(0.015)	(0.013)	(0.013)	(0.014)	(0.014)	(0.014)	(0.014)	(0.014)	(0.014)	(0.014)
Observations	4,186	4,186	4,186	4,186	4,186	4,186	4,186	4,186	4,186	4,186	4,186
R-squared	0.166	0.160	0.143	0.129	0.119	0.117	0.117	0.114	0.112	0.117	0.114
Country fixed-effects	✓	✓	✓	✓	✓	✓	✓	✓	✓	✓	✓

Note: Results are from OLS regression models. The dependent variable in all models is the proportion of the available amount returned by second movers. Data are from a representative sample of respondents in Italy, Japan and the UK.

Table F24: Proportion returned and personality traits (openness)

VARIABLES	(1) 0 CU	(2) 1 CU	(3) 2 CU	(4) 3 CU	(5) 4 CU	(6) 5 CU	(7) 6 CU	(8) 7 CU	(9) 8 CU	(10) 9 CU	(11) 10 CU
Openness	0.005**	0.002	0.002	0.001	0.000	-0.001	-0.001	-0.002	-0.002	-0.002	-0.002
	(0.002)	(0.002)	(0.002)	(0.002)	(0.002)	(0.002)	(0.002)	(0.002)	(0.002)	(0.002)	(0.002)
Italy	0.039***	0.025**	0.024***	0.024***	0.019**	0.018**	0.025***	0.016*	0.020**	0.022**	0.019**
	(0.013)	(0.010)	(0.009)	(0.009)	(0.009)	(0.009)	(0.009)	(0.009)	(0.009)	(0.009)	(0.009)
Japan	-0.016	-0.029***	-0.021***	-0.009	-0.013*	-0.011	-0.013*	-0.018**	-0.011	-0.005	-0.008
	(0.011)	(0.009)	(0.008)	(0.008)	(0.008)	(0.008)	(0.008)	(0.008)	(0.008)	(0.008)	(0.008)
Constant	0.136***	0.229***	0.277***	0.308***	0.340***	0.371***	0.374***	0.395***	0.400***	0.403***	0.422***
	(0.020)	(0.015)	(0.014)	(0.013)	(0.014)	(0.014)	(0.013)	(0.014)	(0.014)	(0.014)	(0.014)
Observations	4,186	4,186	4,186	4,186	4,186	4,186	4,186	4,186	4,186	4,186	4,186
R-squared	0.008	0.012	0.010	0.005	0.004	0.003	0.006	0.004	0.004	0.003	0.003

Note: Results are from OLS regression models. The dependent variable in all models is the proportion of the available amount returned by second movers. Data are from a representative sample of respondents in Italy, Japan and the UK.

Table F25: Proportion returned and personality traits (openness), including controls

VARIABLES	(1) 0 CU	(2) 1 CU	(3) 2 CU	(4) 3 CU	(5) 4 CU	(6) 5 CU	(7) 6 CU	(8) 7 CU	(9) 8 CU	(10) 9 CU	(11) 10 CU
Openness	0.001	-0.000	-0.000	-0.001	-0.002	-0.003*	-0.003	-0.003**	-0.004**	-0.004**	-0.004**
	(0.002)	(0.002)	(0.002)	(0.001)	(0.002)	(0.002)	(0.002)	(0.002)	(0.002)	(0.002)	(0.002)
Conditional cooperation	-0.061***	-0.031***	-0.013***	-0.002	0.006*	0.012***	0.018***	0.024***	0.027***	0.032***	0.032***
	(0.004)	(0.003)	(0.003)	(0.003)	(0.003)	(0.003)	(0.003)	(0.003)	(0.003)	(0.003)	(0.003)
Altruism ORP (standardised)	0.099***	0.079***	0.070***	0.067***	0.066***	0.066***	0.063***	0.063***	0.062***	0.063***	0.063***
	(0.004)	(0.003)	(0.003)	(0.003)	(0.003)	(0.003)	(0.003)	(0.003)	(0.003)	(0.003)	(0.003)
Risk proclivity	0.005*	0.006***	0.006***	0.005***	0.005***	0.005***	0.004***	0.005***	0.006***	0.005***	0.006***
	(0.002)	(0.002)	(0.002)	(0.002)	(0.002)	(0.002)	(0.002)	(0.002)	(0.002)	(0.002)	(0.002)
Constant	0.153***	0.235***	0.280***	0.310***	0.341***	0.371***	0.374***	0.392***	0.395***	0.399***	0.416***
	(0.019)	(0.015)	(0.013)	(0.013)	(0.013)	(0.014)	(0.013)	(0.014)	(0.014)	(0.014)	(0.014)
Observations	4,186	4,186	4,186	4,186	4,186	4,186	4,186	4,186	4,186	4,186	4,186
R-squared	0.166	0.160	0.143	0.129	0.119	0.118	0.117	0.115	0.113	0.118	0.115
Country fixed-effects	✓	✓	✓	✓	✓	✓	✓	✓	✓	✓	✓

Note: Results are from OLS regression models. The dependent variable in all models is the proportion of the available amount returned by second movers. Data are from a representative sample of respondents in Italy, Japan and the UK.

Table F26: Proportion returned and personality traits (extraversion)

VARIABLES	(1)<br>0 CU	(2)<br>1 CU	(3)<br>2 CU	(4)<br>3 CU	(5)<br>4 CU	(6)<br>5 CU	(7)<br>6 CU	(8)<br>7 CU	(9)<br>8 CU	(10)<br>9 CU	(11)<br>10 CU
Extraversion	0.016***	0.009***	0.007***	0.006***	0.005***	0.004***	0.003*	0.002	0.002	0.001	0.002
	(0.002)	(0.002)	(0.002)	(0.002)	(0.002)	(0.002)	(0.002)	(0.002)	(0.002)	(0.002)	(0.002)
Italy	0.037***	0.024**	0.023***	0.023***	0.019**	0.018**	0.025***	0.016*	0.020**	0.022**	0.019**
	(0.013)	(0.010)	(0.009)	(0.009)	(0.009)	(0.009)	(0.009)	(0.009)	(0.009)	(0.009)	(0.009)
Japan	-0.007	-0.024***	-0.017**	-0.005	-0.009	-0.006	-0.010	-0.014*	-0.008	-0.002	-0.005
	(0.011)	(0.009)	(0.008)	(0.007)	(0.008)	(0.008)	(0.008)	(0.008)	(0.008)	(0.008)	(0.008)
Constant	0.122***	0.217***	0.269***	0.297***	0.327***	0.352***	0.359***	0.375***	0.381***	0.386***	0.403***
	(0.012)	(0.009)	(0.008)	(0.008)	(0.008)	(0.008)	(0.008)	(0.008)	(0.008)	(0.008)	(0.009)
Observations	4,186	4,186	4,186	4,186	4,186	4,186	4,186	4,186	4,186	4,186	4,186
R-squared	0.017	0.018	0.014	0.008	0.007	0.005	0.007	0.005	0.004	0.003	0.003

Note: Results are from OLS regression models. The dependent variable in all models is the proportion of the available amount returned by second movers. Data are from a representative sample of respondents in Italy, Japan and the UK.

Table F27: Proportion returned and personality traits (extraversion), including controls

VARIABLES	(1)<br>0 CU	(2)<br>1 CU	(3)<br>2 CU	(4)<br>3 CU	(5)<br>4 CU	(6)<br>5 CU	(7)<br>6 CU	(8)<br>7 CU	(9)<br>8 CU	(10)<br>9 CU	(11)<br>10 CU
Extraversion	0.011***	0.006***	0.004***	0.004**	0.003*	0.002	0.001	0.001	0.000	-0.000	0.000
	(0.002)	(0.002)	(0.002)	(0.002)	(0.002)	(0.002)	(0.002)	(0.002)	(0.002)	(0.002)	(0.002)
Conditional cooperation	-0.060***	-0.030***	-0.013***	-0.001	0.006**	0.012***	0.018***	0.024***	0.027***	0.032***	0.032***
	(0.004)	(0.003)	(0.003)	(0.003)	(0.003)	(0.003)	(0.003)	(0.003)	(0.003)	(0.003)	(0.003)
Altruism ORP (standardised)	0.098***	0.079***	0.070***	0.067***	0.065***	0.065***	0.062***	0.063***	0.061***	0.063***	0.062***
	(0.004)	(0.003)	(0.003)	(0.003)	(0.003)	(0.003)	(0.003)	(0.003)	(0.003)	(0.003)	(0.003)
Risk proclivity	0.004*	0.006***	0.005***	0.005***	0.005***	0.005***	0.004**	0.005***	0.006***	0.005***	0.006***
	(0.002)	(0.002)	(0.002)	(0.002)	(0.002)	(0.002)	(0.002)	(0.002)	(0.002)	(0.002)	(0.002)
Constant	0.131***	0.216***	0.264***	0.291***	0.321***	0.344***	0.351***	0.364***	0.368***	0.374***	0.388***
	(0.013)	(0.010)	(0.009)	(0.009)	(0.009)	(0.009)	(0.009)	(0.009)	(0.009)	(0.009)	(0.010)
Observations	4,186	4,186	4,186	4,186	4,186	4,186	4,186	4,186	4,186	4,186	4,186
R-squared	0.170	0.163	0.145	0.131	0.120	0.118	0.117	0.114	0.112	0.117	0.114
Country fixed-effects	✓	✓	✓	✓	✓	✓	✓	✓	✓	✓	✓

Note: Results are from OLS regression models. The dependent variable in all models is the proportion of the available amount returned by second movers. Data are from a representative sample of respondents in Italy, Japan and the UK.

Table F28: Proportion returned and personality traits (neuroticism)

	(1)	(2)	(3)	(4)	(5)	(6)	(7)	(8)	(9)	(10)	(11)
VARIABLES	0 CU	1 CU	2 CU	3 CU	4 CU	5 CU	6 CU	7 CU	8 CU	9 CU	10 CU
Neuroticism	-0.011***	-0.005***	-0.004***	-0.003**	-0.002	-0.003*	-0.002	-0.002	-0.002	-0.001	-0.001
	(0.002)	(0.002)	(0.001)	(0.001)	(0.001)	(0.001)	(0.001)	(0.002)	(0.002)	(0.002)	(0.002)
Italy	0.038***	0.025**	0.023***	0.024***	0.019**	0.018**	0.025***	0.016*	0.020**	0.022**	0.019**
	(0.013)	(0.010)	(0.009)	(0.009)	(0.009)	(0.009)	(0.009)	(0.009)	(0.009)	(0.009)	(0.009)
Japan	-0.008	-0.026***	-0.019**	-0.006	-0.011	-0.007	-0.010	-0.014*	-0.007	-0.001	-0.005
	(0.011)	(0.009)	(0.008)	(0.008)	(0.008)	(0.008)	(0.008)	(0.008)	(0.008)	(0.008)	(0.008)
Constant	0.204***	0.260***	0.302***	0.324***	0.349***	0.373***	0.373***	0.388***	0.392***	0.393***	0.412***
	(0.011)	(0.008)	(0.007)	(0.007)	(0.007)	(0.007)	(0.007)	(0.008)	(0.008)	(0.008)	(0.008)
Observations	4,186	4,186	4,186	4,186	4,186	4,186	4,186	4,186	4,186	4,186	4,186
R-squared	0.013	0.013	0.011	0.006	0.005	0.004	0.006	0.005	0.004	0.003	0.002

Note: Results are from OLS regression models. The dependent variable in all models is the proportion of the available amount returned by second movers. Data are from a representative sample of respondents in Italy, Japan and the UK.

Table F29: Proportion returned and personality traits (neuroticism), including controls

VARIABLES	(1)<br>0 CU	(2)<br>1 CU	(3)<br>2 CU	(4)<br>3 CU	(5)<br>4 CU	(6)<br>5 CU	(7)<br>6 CU	(8)<br>7 CU	(9)<br>8 CU	(10)<br>9 CU	(11)<br>10 CU
Neuroticism	-0.008***	-0.002	-0.002	-0.001	-0.001	-0.002	-0.001	-0.001	-0.001	-0.001	-0.000
	(0.002)	(0.002)	(0.001)	(0.001)	(0.001)	(0.001)	(0.001)	(0.001)	(0.001)	(0.001)	(0.001)
Conditional cooperation	-0.060***	-0.030***	-0.013***	-0.001	0.006*	0.012***	0.018***	0.024***	0.027***	0.032***	0.032***
	(0.004)	(0.003)	(0.003)	(0.003)	(0.003)	(0.003)	(0.003)	(0.003)	(0.003)	(0.003)	(0.003)
Altruism ORP (standardised)	0.099***	0.079***	0.070***	0.067***	0.066***	0.065***	0.063***	0.063***	0.061***	0.063***	0.062***
	(0.004)	(0.003)	(0.003)	(0.003)	(0.003)	(0.003)	(0.003)	(0.003)	(0.003)	(0.003)	(0.003)
Risk proclivity	0.005*	0.006***	0.005***	0.005***	0.005***	0.005***	0.004**	0.005***	0.006***	0.005***	0.006***
	(0.002)	(0.002)	(0.002)	(0.002)	(0.002)	(0.002)	(0.002)	(0.002)	(0.002)	(0.002)	(0.002)
Constant	0.186***	0.240***	0.283***	0.306***	0.332***	0.356***	0.357***	0.370***	0.373***	0.376***	0.391***
	(0.012)	(0.009)	(0.009)	(0.008)	(0.009)	(0.009)	(0.009)	(0.009)	(0.009)	(0.009)	(0.009)
Observations	4,186	4,186	4,186	4,186	4,186	4,186	4,186	4,186	4,186	4,186	4,186
R-squared	0.169	0.161	0.144	0.130	0.119	0.118	0.117	0.114	0.112	0.117	0.114
Country fixed-effects	✓	✓	✓	✓	✓	✓	✓	✓	✓	✓	✓

Note: Results are from OLS regression models. The dependent variable in all models is the proportion of the available amount returned by second movers. Data are from a representative sample of respondents in Italy, Japan and the UK.

F.7 Martial status

Table F30: Proportion returned and civil status (married) in Italy

	(1)	(2)	(3)	(4)	(5)	(6)	(7)	(8)	(9)	(10)	(11)
VARIABLES	0 CU	1 CU	2 CU	3 CU	4 CU	5 CU	6 CU	7 CU	8 CU	9 CU	10 CU
Married	0.026	-0.003	-0.014	-0.025*	-0.028**	-0.037***	-0.033**	-0.033**	-0.034**	-0.040***	-0.040***
	(0.019)	(0.015)	(0.013)	(0.013)	(0.013)	(0.014)	(0.014)	(0.014)	(0.014)	(0.015)	(0.015)
Constant	0.197***	0.273***	0.322***	0.353***	0.378***	0.403***	0.411***	0.417***	0.426***	0.433***	0.450***
	(0.014)	(0.011)	(0.010)	(0.010)	(0.010)	(0.010)	(0.010)	(0.010)	(0.011)	(0.011)	(0.011)
Observations	998	998	998	998	998	998	998	998	998	998	998
R-squared	0.002	0.000	0.001	0.004	0.005	0.007	0.006	0.005	0.006	0.008	0.008

Note: Results are from OLS regression models. The dependent variable in all models is the proportion of the available amount returned by second movers. Data are from a representative sample of respondents in Italy.

Table F31: Proportion returned and civil status (married) in Italy, including controls

	(1)	(2)	(3)	(4)	(5)	(6)	(7)	(8)	(9)	(10)	(11)
VARIABLES	0 CU	1 CU	2 CU	3 CU	4 CU	5 CU	6 CU	7 CU	8 CU	9 CU	10 CU
Married	0.010	-0.014	-0.022*	-0.030**	-0.033***	-0.040***	-0.036***	-0.035***	-0.035***	-0.041***	-0.041***
	(0.018)	(0.014)	(0.013)	(0.012)	(0.012)	(0.013)	(0.013)	(0.013)	(0.013)	(0.014)	(0.014)
Conditional cooperation	-0.062***	-0.030***	-0.012**	-0.001	0.007	0.012*	0.014**	0.022***	0.025***	0.033***	0.034***
	(0.009)	(0.007)	(0.006)	(0.006)	(0.006)	(0.006)	(0.006)	(0.007)	(0.007)	(0.007)	(0.007)
Altruism ORP (standardised)	0.100***	0.094***	0.084***	0.079***	0.084***	0.083***	0.076***	0.081***	0.081***	0.082***	0.080***
	(0.010)	(0.007)	(0.007)	(0.007)	(0.007)	(0.007)	(0.007)	(0.007)	(0.007)	(0.007)	(0.008)
Risk proclivity	0.006	0.004	0.003	0.000	0.001	-0.000	-0.001	-0.001	0.000	-0.000	0.001
	(0.006)	(0.004)	(0.004)	(0.004)	(0.004)	(0.004)	(0.004)	(0.004)	(0.004)	(0.004)	(0.004)
Constant	0.180***	0.264***	0.317***	0.354***	0.379***	0.406***	0.418***	0.422***	0.429***	0.437***	0.450***
	(0.020)	(0.016)	(0.014)	(0.014)	(0.014)	(0.014)	(0.015)	(0.015)	(0.015)	(0.016)	(0.016)
Observations	998	998	998	998	998	998	998	998	998	998	998
R-squared	0.148	0.164	0.143	0.127	0.138	0.133	0.113	0.122	0.123	0.129	0.124
Country fixed-effects	✓	✓	✓	✓	✓	✓	✓	✓	✓	✓	✓

Note: Results are from OLS regression models. The dependent variable in all models is the proportion of the available amount returned by second movers. Data are from a representative sample of respondents in Italy.

Table F32: Proportion returned and civil status (divorced) in Italy

VARIABLES	(1)<br>0 CU	(2)<br>1 CU	(3)<br>2 CU	(4)<br>3 CU	(5)<br>4 CU	(6)<br>5 CU	(7)<br>6 CU	(8)<br>7 CU	(9)<br>8 CU	(10)<br>9 CU	(11)<br>10 CU
Divorced	0.002	-0.035	-0.030	-0.010	-0.006	0.002	-0.012	-0.014	-0.009	-0.007	-0.006
	(0.035)	(0.027)	(0.025)	(0.024)	(0.024)	(0.025)	(0.025)	(0.026)	(0.026)	(0.027)	(0.027)
Constant	0.211***	0.275***	0.317***	0.340***	0.363***	0.383***	0.394***	0.400***	0.408***	0.412***	0.428***
	(0.010)	(0.008)	(0.007)	(0.007)	(0.007)	(0.007)	(0.007)	(0.007)	(0.007)	(0.008)	(0.008)
Observations	998	998	998	998	998	998	998	998	998	998	998
R-squared	0.000	0.002	0.001	0.000	0.000	0.000	0.000	0.000	0.000	0.000	0.000

Note: Results are from OLS regression models. The dependent variable in all models is the proportion of the available amount returned by second movers. Data are from a representative sample of respondents in Italy.

Table F33: Proportion returned and civil status (divorced) in Italy, including controls

VARIABLES	(1)<br>0 CU	(2)<br>1 CU	(3)<br>2 CU	(4)<br>3 CU	(5)<br>4 CU	(6)<br>5 CU	(7)<br>6 CU	(8)<br>7 CU	(9)<br>8 CU	(10)<br>9 CU	(11)<br>10 CU
Divorced	-0.004	-0.037	-0.030	-0.008	-0.003	0.005	-0.008	-0.009	-0.004	-0.000	0.001
	(0.033)	(0.025)	(0.023)	(0.022)	(0.023)	(0.023)	(0.024)	(0.024)	(0.024)	(0.025)	(0.025)
Conditional cooperation	-0.062***	-0.030***	-0.012*	0.000	0.008	0.013**	0.015**	0.023***	0.026***	0.034***	0.035***
	(0.009)	(0.007)	(0.006)	(0.006)	(0.006)	(0.006)	(0.006)	(0.007)	(0.007)	(0.007)	(0.007)
Altruism ORP (standardised)	0.100***	0.094***	0.084***	0.078***	0.083***	0.082***	0.076***	0.081***	0.080***	0.081***	0.079***
	(0.010)	(0.007)	(0.007)	(0.007)	(0.007)	(0.007)	(0.007)	(0.007)	(0.007)	(0.007)	(0.008)
Risk proclivity	0.006	0.004	0.002	-0.000	-0.000	-0.001	-0.002	-0.001	-0.001	-0.001	0.001
	(0.006)	(0.004)	(0.004)	(0.004)	(0.004)	(0.004)	(0.004)	(0.004)	(0.004)	(0.004)	(0.004)
Constant	0.186***	0.260***	0.309***	0.340***	0.363***	0.386***	0.401***	0.406***	0.412***	0.417***	0.430***
	(0.019)	(0.014)	(0.013)	(0.013)	(0.013)	(0.013)	(0.013)	(0.014)	(0.014)	(0.014)	(0.014)
Observations	998	998	998	998	998	998	998	998	998	998	998
R-squared	0.148	0.165	0.142	0.122	0.132	0.124	0.107	0.117	0.117	0.121	0.116
Country fixed-effects	✓	✓	✓	✓	✓	✓	✓	✓	✓	✓	✓

Note: Results are from OLS regression models. The dependent variable in all models is the proportion of the available amount returned by second movers. Data are from a representative sample of respondents in Italy.

F.8 Political views

Table F34: Proportion returned and political views (against immigration)

VARIABLES	(1)<br>0 CU	(2)<br>1 CU	(3)<br>2 CU	(4)<br>3 CU	(5)<br>4 CU	(6)<br>5 CU	(7)<br>6 CU	(8)<br>7 CU	(9)<br>8 CU	(10)<br>9 CU	(11)<br>10 CU
Against immigration	0.020**	-0.001	-0.005	-0.007	-0.011**	-0.013**	-0.014**	-0.013**	-0.016***	-0.017***	-0.018***
	(0.008)	(0.006)	(0.006)	(0.006)	(0.006)	(0.006)	(0.006)	(0.006)	(0.006)	(0.006)	(0.006)
UK	-0.024*	-0.002	-0.007	-0.012	-0.012	-0.008	-0.013	-0.010	-0.013	-0.013	-0.011
	(0.014)	(0.010)	(0.009)	(0.009)	(0.009)	(0.009)	(0.009)	(0.009)	(0.009)	(0.010)	(0.010)
Italy	0.017	0.024**	0.018*	0.014	0.010	0.012	0.014	0.009	0.011	0.012	0.011
	(0.014)	(0.010)	(0.009)	(0.009)	(0.009)	(0.009)	(0.009)	(0.009)	(0.009)	(0.010)	(0.010)
Japan	-0.041***	-0.031***	-0.028***	-0.019**	-0.022***	-0.015*	-0.023***	-0.023***	-0.019**	-0.013	-0.015*
	(0.012)	(0.009)	(0.008)	(0.008)	(0.008)	(0.008)	(0.008)	(0.008)	(0.008)	(0.008)	(0.008)
USA	0.062***	0.042***	0.026***	0.014	0.003	-0.002	-0.005	-0.011	-0.011	-0.011	-0.012
	(0.013)	(0.010)	(0.009)	(0.009)	(0.009)	(0.009)	(0.009)	(0.009)	(0.009)	(0.010)	(0.010)
Constant	0.186***	0.248***	0.300***	0.329***	0.358***	0.377***	0.386***	0.396***	0.405***	0.407***	0.426***
	(0.010)	(0.008)	(0.007)	(0.007)	(0.007)	(0.007)	(0.007)	(0.007)	(0.007)	(0.007)	(0.007)
Observations	5,870	5,870	5,870	5,870	5,870	5,870	5,870	5,870	5,870	5,870	5,870
R-squared	0.015	0.014	0.011	0.005	0.005	0.003	0.005	0.004	0.004	0.003	0.003

Note: Results are from OLS regression models. The dependent variable in all models is the proportion of the available amount returned by second movers. Data are from a representative sample of respondents in Germany, Italy, Japan, the UK, and the USA.

Table F35: Proportion returned and political views (against immigration), including controls

VARIABLES	(1)<br>0 CU	(2)<br>1 CU	(3)<br>2 CU	(4)<br>3 CU	(5)<br>4 CU	(6)<br>5 CU	(7)<br>6 CU	(8)<br>7 CU	(9)<br>8 CU	(10)<br>9 CU	(11)<br>10 CU
Against immigration	0.019**	-0.001	-0.005	-0.006	-0.010*	-0.011**	-0.013**	-0.011**	-0.015***	-0.015***	-0.016***
	(0.008)	(0.006)	(0.005)	(0.005)	(0.005)	(0.005)	(0.005)	(0.006)	(0.006)	(0.006)	(0.006)
Conditional cooperation	-0.070***	-0.039***	-0.021***	-0.007***	0.001	0.007***	0.014***	0.019***	0.022***	0.027***	0.028***
	(0.004)	(0.003)	(0.003)	(0.003)	(0.003)	(0.003)	(0.003)	(0.003)	(0.003)	(0.003)	(0.003)
Altruism ORP (standardised)	0.098***	0.079***	0.071***	0.068***	0.066***	0.066***	0.062***	0.063***	0.061***	0.062***	0.061***
	(0.004)	(0.003)	(0.003)	(0.002)	(0.003)	(0.003)	(0.003)	(0.003)	(0.003)	(0.003)	(0.003)
Risk proclivity	0.004**	0.003*	0.003**	0.003**	0.002	0.003*	0.003*	0.003*	0.003**	0.002	0.004**
	(0.002)	(0.002)	(0.001)	(0.001)	(0.001)	(0.001)	(0.001)	(0.002)	(0.002)	(0.002)	(0.002)
Constant	0.152***	0.225***	0.279***	0.310***	0.343***	0.360***	0.372***	0.381***	0.389***	0.395***	0.410***
	(0.011)	(0.008)	(0.008)	(0.008)	(0.008)	(0.008)	(0.008)	(0.008)	(0.008)	(0.008)	(0.008)
Observations	5,870	5,870	5,870	5,870	5,870	5,870	5,870	5,870	5,870	5,870	5,870
R-squared	0.181	0.168	0.142	0.123	0.111	0.108	0.103	0.099	0.097	0.100	0.099
Country fixed-effects	✓	✓	✓	✓	✓	✓	✓	✓	✓	✓	✓

Note: Results are from OLS regression models. The dependent variable in all models is the proportion of the available amount returned by second movers. Data are from a representative sample of respondents in Germany, Italy, Japan, the UK, and the USA.

Table F36: Proportion returned and political views (social mobility)

VARIABLES	(1)<br>0 CU	(2)<br>1 CU	(3)<br>2 CU	(4)<br>3 CU	(5)<br>4 CU	(6)<br>5 CU	(7)<br>6 CU	(8)<br>7 CU	(9)<br>8 CU	(10)<br>9 CU	(11)<br>10 CU
Social mobility is low	-0.022***	-0.016***	-0.011**	-0.009*	-0.008	-0.011**	-0.008	-0.007	-0.006	-0.007	-0.005
	(0.008)	(0.006)	(0.005)	(0.005)	(0.005)	(0.005)	(0.005)	(0.005)	(0.005)	(0.005)	(0.005)
UK	-0.021	-0.002	-0.008	-0.012	-0.011	-0.007	-0.013	-0.008	-0.011	-0.012	-0.011
	(0.013)	(0.010)	(0.009)	(0.009)	(0.009)	(0.009)	(0.009)	(0.009)	(0.009)	(0.009)	(0.010)
Italy	0.017	0.023**	0.016*	0.013	0.010	0.012	0.013	0.008	0.010	0.011	0.009
	(0.014)	(0.010)	(0.009)	(0.009)	(0.009)	(0.009)	(0.009)	(0.009)	(0.009)	(0.009)	(0.010)
Japan	-0.041***	-0.032***	-0.030***	-0.020***	-0.023***	-0.015*	-0.023***	-0.023***	-0.019**	-0.013	-0.016*
	(0.012)	(0.009)	(0.008)	(0.008)	(0.008)	(0.008)	(0.008)	(0.008)	(0.008)	(0.008)	(0.008)
Luxembourg	0.008	0.014	0.014	0.013	0.014	0.015	0.006	0.006	0.008	0.011	0.009
	(0.014)	(0.010)	(0.009)	(0.009)	(0.009)	(0.009)	(0.009)	(0.009)	(0.009)	(0.009)	(0.010)
USA	0.059***	0.041***	0.023***	0.011	0.002	-0.004	-0.007	-0.012	-0.012	-0.012	-0.011
	(0.013)	(0.010)	(0.009)	(0.009)	(0.009)	(0.009)	(0.009)	(0.009)	(0.009)	(0.009)	(0.009)
Constant	0.201***	0.254***	0.303***	0.331***	0.356***	0.376***	0.384***	0.394***	0.400***	0.403***	0.422***
	(0.010)	(0.007)	(0.007)	(0.006)	(0.007)	(0.007)	(0.007)	(0.007)	(0.007)	(0.007)	(0.007)
Observations	7,045	7,045	7,045	7,045	7,045	7,045	7,045	7,045	7,045	7,045	7,045
R-squared	0.014	0.015	0.011	0.006	0.005	0.004	0.005	0.004	0.003	0.003	0.003

Note: Results are from OLS regression models. The dependent variable in all models is the proportion of the available amount returned by second movers. Data are from a representative sample of respondents in Germany, Italy, Japan, the UK, and the USA.

Table F37: Proportion returned and political views (social mobility), including controls

VARIABLES	(1)<br>0 CU	(2)<br>1 CU	(3)<br>2 CU	(4)<br>3 CU	(5)<br>4 CU	(6)<br>5 CU	(7)<br>6 CU	(8)<br>7 CU	(9)<br>8 CU	(10)<br>9 CU	(11)<br>10 CU
Social mobility is low	-0.008	-0.006	-0.003	-0.002	-0.002	-0.005	-0.003	-0.003	-0.002	-0.003	-0.002
	(0.007)	(0.005)	(0.005)	(0.005)	(0.005)	(0.005)	(0.005)	(0.005)	(0.005)	(0.005)	(0.005)
Conditional cooperation	-0.071***	-0.041***	-0.022***	-0.009***	-0.001	0.006**	0.011***	0.017***	0.020***	0.025***	0.026***
	(0.003)	(0.002)	(0.002)	(0.002)	(0.002)	(0.002)	(0.002)	(0.002)	(0.002)	(0.002)	(0.002)
Altruism ORP (standardised)	0.100***	0.080***	0.071***	0.067***	0.065***	0.065***	0.062***	0.062***	0.060***	0.060***	0.060***
	(0.003)	(0.003)	(0.002)	(0.002)	(0.002)	(0.002)	(0.002)	(0.002)	(0.002)	(0.002)	(0.002)
Risk proclivity	0.006***	0.004***	0.004***	0.004***	0.003**	0.004***	0.003**	0.004***	0.004***	0.004**	0.004***
	(0.002)	(0.001)	(0.001)	(0.001)	(0.001)	(0.001)	(0.001)	(0.001)	(0.001)	(0.001)	(0.001)
Constant	0.158***	0.224***	0.277***	0.307***	0.337***	0.355***	0.367***	0.375***	0.382***	0.387***	0.404***
	(0.011)	(0.008)	(0.007)	(0.007)	(0.007)	(0.007)	(0.007)	(0.008)	(0.008)	(0.008)	(0.008)
Observations	7,045	7,045	7,045	7,045	7,045	7,045	7,045	7,045	7,045	7,045	7,045
R-squared	0.184	0.175	0.144	0.123	0.108	0.104	0.099	0.094	0.092	0.094	0.094
Country fixed-effects	✓	✓	✓	✓	✓	✓	✓	✓	✓	✓	✓

Note: Results are from OLS regression models. The dependent variable in all models is the proportion of the available amount returned by second movers. Data are from a representative sample of respondents in Germany, Italy, Japan, the UK, and the USA.

Table F38: Proportion returned and political views (political efficacy)

VARIABLES	(1)<br>0 CU	(2)<br>1 CU	(3)<br>2 CU	(4)<br>3 CU	(5)<br>4 CU	(6)<br>5 CU	(7)<br>6 CU	(8)<br>7 CU	(9)<br>8 CU	(10)<br>9 CU	(11)<br>10 CU
Low political efficacy	-0.020**	-0.014**	-0.010*	-0.011*	-0.006	-0.002	-0.000	0.002	0.004	0.003	0.004
	(0.009)	(0.007)	(0.006)	(0.006)	(0.006)	(0.006)	(0.006)	(0.006)	(0.006)	(0.006)	(0.006)
UK	-0.022*	-0.002	-0.008	-0.012	-0.011	-0.008	-0.013	-0.010	-0.013	-0.014	-0.011
	(0.013)	(0.010)	(0.009)	(0.009)	(0.009)	(0.009)	(0.009)	(0.009)	(0.009)	(0.010)	(0.010)
Italy	0.017	0.023**	0.017*	0.014	0.009	0.012	0.014	0.010	0.011	0.012	0.011
	(0.014)	(0.010)	(0.009)	(0.009)	(0.009)	(0.009)	(0.009)	(0.009)	(0.009)	(0.010)	(0.010)
Japan	-0.034***	-0.027***	-0.024***	-0.015*	-0.021**	-0.015*	-0.024***	-0.024***	-0.021**	-0.014*	-0.016*
	(0.012)	(0.009)	(0.008)	(0.008)	(0.008)	(0.008)	(0.008)	(0.008)	(0.008)	(0.009)	(0.009)
USA	0.059***	0.042***	0.025***	0.013	0.002	-0.003	-0.006	-0.011	-0.012	-0.012	-0.012
	(0.013)	(0.010)	(0.009)	(0.009)	(0.009)	(0.009)	(0.009)	(0.009)	(0.009)	(0.009)	(0.010)
Constant	0.197***	0.251***	0.300***	0.329***	0.355***	0.373***	0.381***	0.391***	0.398***	0.401***	0.419***
	(0.010)	(0.007)	(0.007)	(0.007)	(0.007)	(0.007)	(0.007)	(0.007)	(0.007)	(0.007)	(0.007)
Observations	6,007	6,007	6,007	6,007	6,007	6,007	6,007	6,007	6,007	6,007	6,007
R-squared	0.015	0.015	0.011	0.005	0.004	0.002	0.004	0.003	0.003	0.002	0.002

Note: Results are from OLS regression models. The dependent variable in all models is the proportion of the available amount returned by second movers. Data are from a representative sample of respondents in Germany, Italy, Japan, the UK, and the USA.

Table F39: Proportion returned and political views (political efficacy), including controls

VARIABLES	(1)<br>0 CU	(2)<br>1 CU	(3)<br>2 CU	(4)<br>3 CU	(5)<br>4 CU	(6)<br>5 CU	(7)<br>6 CU	(8)<br>7 CU	(9)<br>8 CU	(10)<br>9 CU	(11)<br>10 CU
Low political efficacy	-0.013*	-0.009	-0.006	-0.008	-0.003	0.001	0.002	0.005	0.006	0.005	0.005
	(0.008)	(0.006)	(0.006)	(0.005)	(0.006)	(0.006)	(0.006)	(0.006)	(0.006)	(0.006)	(0.006)
Conditional cooperation	-0.070***	-0.040***	-0.021***	-0.008***	0.001	0.007***	0.014***	0.019***	0.022***	0.027***	0.028***
	(0.004)	(0.003)	(0.003)	(0.002)	(0.003)	(0.003)	(0.003)	(0.003)	(0.003)	(0.003)	(0.003)
Altruism ORP (standardised)	0.099***	0.079***	0.070***	0.067***	0.066***	0.066***	0.062***	0.063***	0.061***	0.062***	0.062***
	(0.004)	(0.003)	(0.002)	(0.002)	(0.002)	(0.003)	(0.002)	(0.003)	(0.003)	(0.003)	(0.003)
Risk proclivity	0.005**	0.004**	0.003**	0.004***	0.002*	0.003**	0.003*	0.003**	0.004**	0.003*	0.004**
	(0.002)	(0.002)	(0.001)	(0.001)	(0.001)	(0.001)	(0.001)	(0.002)	(0.002)	(0.002)	(0.002)
Constant	0.161***	0.225***	0.277***	0.308***	0.338***	0.355***	0.366***	0.375***	0.382***	0.387***	0.402***
	(0.011)	(0.008)	(0.008)	(0.007)	(0.008)	(0.008)	(0.008)	(0.008)	(0.008)	(0.008)	(0.008)
Observations	6,007	6,007	6,007	6,007	6,007	6,007	6,007	6,007	6,007	6,007	6,007
R-squared	0.180	0.168	0.142	0.123	0.111	0.107	0.103	0.100	0.098	0.100	0.100
Country fixed-effects	✓	✓	✓	✓	✓	✓	✓	✓	✓	✓	✓

Note: Results are from OLS regression models. The dependent variable in all models is the proportion of the available amount returned by second movers. Data are from a representative sample of respondents in Germany, Italy, Japan, the UK, and the USA.

G Robustness checks

G.1 Binary and categorical dependent variables

Table G1: Correlates of the amount sent: Predicted probability of sending zero CU (OLS regressions)

VARIABLES	(1)	(2)	(3)	(4)	(5)
Expected return (standardised)	-0.025***				-0.016***
	(0.002)				(0.002)
Altruism (standardised)		-0.039***			-0.034***
		(0.002)			(0.002)
Risk proclivity			-0.006***		-0.004***
			(0.001)		(0.001)
Female				0.003	-0.001
				(0.005)	(0.005)
Non-tertiary diploma				-0.006	-0.008
				(0.006)	(0.006)
University				-0.003	-0.008
				(0.005)	(0.005)
Age				0.000	0.000**
				(0.000)	(0.000)
Respondent born in country				-0.008	-0.010
				(0.009)	(0.008)
Household size				-0.002	-0.001
				(0.002)	(0.002)
Rural				0.007	0.008
				(0.006)	(0.006)
Working				-0.002	0.002
				(0.005)	(0.005)
Religion important				-0.008	-0.002
				(0.005)	(0.005)
Completely satisfied with life				0.004	0.013*
				(0.007)	(0.007)
UK	0.015*	0.010	0.015*	0.016*	0.010
	(0.008)	(0.008)	(0.009)	(0.009)	(0.009)
Italy	0.014	0.004	0.009	0.010	0.006
	(0.009)	(0.008)	(0.009)	(0.009)	(0.009)
Japan	0.038***	0.034***	0.044***	0.049***	0.035***
	(0.007)	(0.007)	(0.007)	(0.008)	(0.008)
Luxembourg	0.000	-0.003	-0.002	-0.010	-0.008
	(0.009)	(0.008)	(0.009)	(0.010)	(0.009)
USA	0.030***	0.030***	0.029***	0.032***	0.032***
	(0.008)	(0.008)	(0.008)	(0.009)	(0.009)
Observations	7,236	7,236	7,236	7,236	7,236
R-squared	0.025	0.048	0.012	0.011	0.057

Note: Results are from OLS regression models. All models control for country fixed effects as well as gender, education level, immigrant and labour force status, household size, whether respondents live in a rural area as well as the importance of religion and life satisfaction. The dependent variable in all models is equal to 1 for trustors who sent zero CU to trustees (n=282, 4%), and 0 otherwise (n=6,954, 96%). Data are from a representative sample of respondents in Germany, Italy, Japan, Luxembourg, the UK and the USA.

Table G2: Correlates of the amount sent: Predicted probability of sending zero CU (probit regressions)

VARIABLES	(1)<br>y1	(2)<br>y1	(3)<br>y1	(4)<br>y1	(5)<br>y1
Expected return (standardised)	-0.027***				-0.017***
	(0.003)				(0.003)
Altruism (standardised)		-0.039***			-0.035***
		(0.003)			(0.003)
Risk proclivity			-0.006***		-0.004***
			(0.001)		(0.001)
Female				0.003	0.003
				(0.005)	(0.004)
Non-tertiary diploma				-0.007	-0.006
				(0.007)	(0.006)
University				-0.004	-0.006
				(0.005)	(0.005)
Age				0.000	0.000
				(0.000)	(0.000)
Respondent born in country				-0.011	-0.012
				(0.010)	(0.009)
Household size				-0.001	0.000
				(0.002)	(0.002)
Rural				0.009	0.012**
				(0.006)	(0.006)
Working				-0.002	-0.000
				(0.005)	(0.005)
Religion important				-0.008	-0.001
				(0.005)	(0.005)
Completely satisfied with life				0.003	0.006
				(0.007)	(0.007)
UK	0.019*	0.014	0.022**	0.022**	0.014
	(0.010)	(0.010)	(0.010)	(0.010)	(0.010)
Italy	0.018*	0.009	0.014	0.014	0.009
	(0.010)	(0.010)	(0.010)	(0.011)	(0.010)
Japan	0.037***	0.030***	0.046***	0.051***	0.031***
	(0.009)	(0.008)	(0.009)	(0.009)	(0.009)
Luxembourg	-0.003	-0.006	-0.005	-0.015	-0.013
	(0.011)	(0.011)	(0.011)	(0.013)	(0.012)
USA	0.034***	0.030***	0.035***	0.038***	0.033***
	(0.009)	(0.009)	(0.010)	(0.010)	(0.009)
Observations	7,236	7,236	7,236	7,236	7,236

Note: Results are from probit regression models. All models control for country fixed effects as well as gender, education level, immigrant and labour force status, household size, whether respondents live in a rural area as well as the importance of religion and life satisfaction. The dependent variable in all models is equal to 1 for trustors who sent zero CU to trustees (n=282, 4%), and 0 otherwise (n=6,954, 96%). Data are from a representative sample of respondents in Germany, Italy, Japan, Luxembourg, the UK and the USA.

Table G3: Correlates of the amount sent: Predicted probability of sending more than half (OLS regressions)

VARIABLES	(1)	(2)	(3)	(4)	(5)
Expected return (standardised)	0.104***				0.071***
	(0.006)				(0.006)
Altruism (standardised)		0.133***			0.112***
		(0.006)			(0.006)
Risk proclivity			0.030***		0.022***
			(0.003)		(0.003)
Female				-0.097***	-0.080***
				(0.011)	(0.011)
Non-tertiary diploma				0.021	0.029*
				(0.016)	(0.015)
University				0.029**	0.045***
				(0.013)	(0.013)
Age				-0.000	-0.001*
				(0.000)	(0.000)
Respondent born in country				0.014	0.021
				(0.021)	(0.020)
Household size				-0.003	-0.007
				(0.005)	(0.004)
Rural				0.000	-0.000
				(0.015)	(0.014)
Working				0.010	-0.008
				(0.012)	(0.012)
Religion important				-0.021*	-0.043***
				(0.013)	(0.012)
Completely satisfied with life				0.056***	0.023
				(0.018)	(0.017)
UK	-0.105***	-0.090***	-0.106***	-0.102***	-0.083***
	(0.021)	(0.020)	(0.021)	(0.022)	(0.021)
Italy	-0.112***	-0.077***	-0.091***	-0.083***	-0.073***
	(0.021)	(0.021)	(0.021)	(0.022)	(0.021)
Japan	-0.156***	-0.150***	-0.182***	-0.190***	-0.138***
	(0.018)	(0.018)	(0.018)	(0.020)	(0.019)
Luxembourg	-0.070***	-0.059***	-0.060***	-0.058**	-0.066***
	(0.021)	(0.021)	(0.021)	(0.024)	(0.023)
USA	-0.083***	-0.082***	-0.082***	-0.075***	-0.077***
	(0.021)	(0.020)	(0.021)	(0.021)	(0.020)
Observations	7,236	7,236	7,236	7,236	7,236
R-squared	0.060	0.090	0.028	0.030	0.127

Note: Results are from OLS regression models. All models control for country fixed effects as well as gender, education level, immigrant and labour force status, household size, whether respondents live in a rural area as well as the importance of religion and life satisfaction. The dependent variable in all models is equal to 1 for trustors who sent more than half their endowment (n=2,669, 37%), and 0 otherwise (n=4,567, 63%). Data are from a representative sample of respondents in Germany, Italy, Japan, Luxembourg, the UK and the USA.

Table G4: Correlates of the amount sent: Predicted probability of sending more than half (probit regressions)

VARIABLES	(1)<br>y1	(2)<br>y1	(3)<br>y1	(4)<br>y1	(5)<br>y1
Expected return (standardised)	0.100***				0.070***
	(0.005)				(0.006)
Altruism (standardised)		0.129***			0.110***
		(0.005)			(0.005)
Risk proclivity			0.029***		0.021***
			(0.003)		(0.003)
Female				-0.097***	-0.079***
				(0.011)	(0.011)
Non-tertiary diploma				0.022	0.030*
				(0.016)	(0.015)
University				0.029**	0.046***
				(0.013)	(0.013)
Age				-0.000	-0.001*
				(0.000)	(0.000)
Respondent born in country				0.013	0.021
				(0.021)	(0.020)
Household size				-0.003	-0.007
				(0.005)	(0.004)
Rural				0.000	-0.001
				(0.015)	(0.014)
Working				0.010	-0.010
				(0.012)	(0.012)
Religion important				-0.020	-0.040***
				(0.013)	(0.012)
Completely satisfied with life				0.055***	0.022
				(0.017)	(0.017)
UK	-0.100***	-0.084***	-0.101***	-0.096***	-0.076***
	(0.020)	(0.020)	(0.021)	(0.021)	(0.020)
Italy	-0.107***	-0.072***	-0.086***	-0.079***	-0.068***
	(0.021)	(0.020)	(0.021)	(0.021)	(0.020)
Japan	-0.154***	-0.150***	-0.181***	-0.188***	-0.137***
	(0.018)	(0.017)	(0.018)	(0.019)	(0.018)
Luxembourg	-0.066***	-0.055***	-0.057***	-0.054**	-0.060***
	(0.020)	(0.020)	(0.021)	(0.023)	(0.022)
USA	-0.079***	-0.078***	-0.078***	-0.071***	-0.073***
	(0.020)	(0.020)	(0.020)	(0.021)	(0.020)
Observations	7,236	7,236	7,236	7,236	7,236

Note: Results are from probit regression models. All models control for country fixed effects as well as gender, education level, immigrant and labour force status, household size, whether respondents live in a rural area as well as the importance of religion and life satisfaction. The dependent variable in all models is equal to 1 for trustors who sent more than half their endowment (n=2,669, 37%), and 0 otherwise (n=4,567, 63%). Data are from a representative sample of respondents in Germany, Italy, Japan, Luxembourg, the UK and the USA.

Table G5: Correlates of the amount sent: Predicted probability of sending less than half, half or more than half (OLS regressions)

VARIABLES	(1)	(2)	(3)	(4)	(5)
Expected return (standardised)	0.185***				0.121***
	(0.009)				(0.009)
Altruism (standardised)		0.252***			0.217***
		(0.009)			(0.009)
Risk proclivity			0.045***		0.031***
			(0.005)		(0.005)
Female				-0.125***	-0.095***
				(0.018)	(0.017)
Non-tertiary diploma				0.050**	0.063***
				(0.025)	(0.024)
University				0.040*	0.069***
				(0.021)	(0.020)
Age				0.001*	0.001
				(0.001)	(0.001)
Respondent born in country				0.010	0.022
				(0.034)	(0.031)
Household size				0.002	-0.004
				(0.007)	(0.007)
Rural				0.008	0.006
				(0.023)	(0.022)
Working				0.032*	0.000
				(0.019)	(0.018)
Religion important				-0.012	-0.052***
				(0.020)	(0.019)
Completely satisfied with life				0.081***	0.021
				(0.028)	(0.026)
UK	-0.180***	-0.151***	-0.182***	-0.166***	-0.130***
	(0.033)	(0.032)	(0.033)	(0.034)	(0.032)
Italy	-0.185***	-0.120***	-0.148***	-0.137***	-0.116***
	(0.033)	(0.032)	(0.034)	(0.035)	(0.032)
Japan	-0.282***	-0.266***	-0.331***	-0.330***	-0.235***
	(0.028)	(0.027)	(0.029)	(0.031)	(0.029)
Luxembourg	-0.090***	-0.071**	-0.072**	-0.075**	-0.089**
	(0.033)	(0.032)	(0.034)	(0.038)	(0.035)
USA	-0.136***	-0.135***	-0.133***	-0.128***	-0.131***
	(0.032)	(0.031)	(0.033)	(0.034)	(0.032)
Observations	7,236	7,236	7,236	7,236	7,236
R-squared	0.079	0.129	0.034	0.034	0.165

Note: Results are from OLS regression models. All models control for country fixed effects as well as gender, education level, immigrant and labour force status, household size, whether respondents live in a rural area as well as the importance of religion and life satisfaction. The dependent variable in all models is equal to 1 for trustors who sent less than half their endowment (23%), 2 for trustors who sent half their endowment (40 %) and 3 for trustors who sent more than half their endowment (37 %). Data are from a representative sample of respondents in Germany, Italy, Japan, Luxembourg, the UK and the USA.

G.2 Larger sample in Japan

Table G6: Correlates of the amount sent with smaller sample for Japan (OLS regressions)

VARIABLES	(1)	(2)	(3)	(4)	(5)
Expected return (standardised)	0.766***				0.519***
	(0.038)				(0.038)
Altruism (standardised)		1.042***			0.897***
		(0.037)			(0.038)
Risk proclivity			0.178***		0.116***
			(0.023)		(0.021)
Female				-0.520***	-0.406***
				(0.077)	(0.072)
Non-tertiary diploma				0.063	0.125
				(0.106)	(0.098)
University				0.124	0.244***
				(0.092)	(0.085)
Age				-0.001	-0.003
				(0.003)	(0.003)
Respondent born in country				-0.029	0.028
				(0.133)	(0.123)
Household size				-0.010	-0.044
				(0.031)	(0.029)
Rural				0.017	0.014
				(0.093)	(0.086)
Working				0.085	-0.031
				(0.083)	(0.077)
Religion important				-0.160*	-0.302***
				(0.085)	(0.079)
Completely satisfied with life				0.329***	0.067
				(0.114)	(0.106)
UK	-0.567***	-0.444***	-0.574***	-0.566***	-0.412***
	(0.128)	(0.124)	(0.131)	(0.135)	(0.125)
Italy	-0.646***	-0.377***	-0.495***	-0.456***	-0.370***
	(0.129)	(0.125)	(0.133)	(0.137)	(0.127)
Japan	-0.960***	-0.911***	-1.174***	-1.208***	-0.792***
	(0.129)	(0.126)	(0.133)	(0.141)	(0.131)
Luxembroug	-0.283**	-0.202	-0.207	-0.244	-0.297**
	(0.130)	(0.126)	(0.133)	(0.149)	(0.137)
USA	-0.499***	-0.495***	-0.488***	-0.431***	-0.449***
	(0.127)	(0.123)	(0.130)	(0.134)	(0.124)
Observations	6,082	6,082	6,082	6,082	6,082
R-squared	0.077	0.129	0.026	0.026	0.169

Note: Results are from OLS regression models. The dependent variable in all models is the amount sent by the first mover. We include a random sub-sample of 1,000 respondents for Japan.

Table G7: Descriptive statistics for Japan (full-sample compared to sub-sample)

	Full sample		Sub-sample		
	mean	sd	mean	sd	p
Female	0.47	0.50	0.50	0.50	0.13
High-school or less	0.30	0.46	0.29	0.45	0.60
Non-tertiary diploma	0.12	0.33	0.12	0.32	0.69
University	0.58	0.49	0.59	0.49	0.45
Age	42.75	13.18	42.36	13.04	0.44
Respondent born in country	1.00	0.04	1.00	0.04	0.93
Household size	2.84	1.30	2.85	1.30	0.72
Rural	0.04	0.20	0.05	0.21	0.46
Working	0.65	0.48	0.64	0.48	0.86
Religion important	0.21	0.40	0.21	0.41	0.80
Completely satisfied with life	0.06	0.24	0.07	0.25	0.57
Social mobility is low	0.44	0.50	0.44	0.50	0.98
Tax burden top 1	42.97	17.51	43.00	18.02	0.96
Tax burden next 9	34.14	11.39	34.38	11.86	0.59
Tax burden next 40	22.71	4.67	22.52	4.86	0.31
Tax burden bottom 50	15.77	11.23	15.90	11.58	0.76
Low political efficacy	0.59	0.49	0.61	0.49	0.39
Against immigration	0.40	0.49	0.40	0.49	0.98
Observations	2154		1000		

Note: Descriptive statistics are shown for the full sample of 2,154 Japanese respondents and a random sub-sample of 1,000 respondents.

Table G8: Correlates of the amount sent with weights (OLS regressions)

VARIABLES	(1)	(2)	(3)	(4)	(5)
Expected return (standardised)	0.764***				0.528***
	(0.039)				(0.039)
Altruism (standardised)		1.021***			0.874***
		(0.039)			(0.040)
Risk proclivity			0.172***		0.111***
			(0.023)		(0.021)
Female				-0.551***	-0.436***
				(0.074)	(0.069)
Non-tertiary diploma				0.115	0.170*
				(0.100)	(0.093)
University				0.197**	0.317***
				(0.087)	(0.081)
Age				-0.001	-0.003
				(0.003)	(0.003)
Respondent born in country				-0.015	0.042
				(0.130)	(0.122)
Household size				-0.015	-0.042
				(0.030)	(0.028)
Rural				0.036	0.034
				(0.090)	(0.083)
Working				0.118	-0.006
				(0.079)	(0.073)
Religion important				-0.114	-0.256***
				(0.081)	(0.075)
Completely satisfied with life				0.314***	0.058
				(0.113)	(0.105)
UK	-0.567***	-0.447***	-0.575***	-0.546***	-0.398***
	(0.122)	(0.122)	(0.127)	(0.131)	(0.122)
Italy	-0.645***	-0.379***	-0.495***	-0.451***	-0.376***
	(0.124)	(0.121)	(0.128)	(0.131)	(0.122)
Japan	-1.013***	-0.953***	-1.218***	-1.250***	-0.853***
	(0.110)	(0.107)	(0.113)	(0.123)	(0.114)
Luxembourg	-0.282**	-0.202*	-0.207	-0.244*	-0.304**
	(0.123)	(0.122)	(0.126)	(0.140)	(0.131)
USA	-0.499***	-0.494***	-0.487***	-0.443***	-0.460***
	(0.123)	(0.120)	(0.127)	(0.131)	(0.121)
Observations	7,236	7,236	7,236	7,236	7,236
R-squared	0.078	0.124	0.025	0.028	0.167

Note: Results are from OLS regression models. The dependent variable in all models is the amount sent by the first mover. Data from Japan are weighted by 0.4 ($\frac{1000}{2500}$) to account for differences in sample size between countries.

G.3 Country-specific regressions: Amount sent

Table G9: Correlates of the amount sent in Germany (OLS regressions)

VARIABLES	(1)	(2)	(3)	(4)	(5)
Expected return (standardised)	0.861***				0.671***
	(0.099)				(0.098)
Altruism (standardised)		0.949***			0.802***
		(0.093)			(0.093)
Risk proclivity			0.156***		0.102**
			(0.052)		(0.049)
Female				-0.533***	-0.328*
				(0.180)	(0.168)
Non-tertiary diploma				-0.381*	-0.344
				(0.225)	(0.209)
University				-0.058	0.066
				(0.234)	(0.218)
Age				0.003	0.002
				(0.007)	(0.006)
Respondent born in country				0.189	0.215
				(0.376)	(0.350)
Household size				0.068	-0.039
				(0.076)	(0.071)
Rural				0.051	0.102
				(0.181)	(0.168)
Working				0.122	0.033
				(0.199)	(0.186)
Religion important				-0.106	-0.257
				(0.197)	(0.184)
Completely satisfied with life				0.676***	0.445*
				(0.261)	(0.243)
Observations	998	998	998	998	998
R-squared	0.071	0.094	0.009	0.021	0.156

Note: Results are from OLS regression models. The dependent variable in all models is the amount sent by the first mover.

Table G10: Potential determinants of trusting behaviour in Germany (Shapley values)

	Shapley values				
	(1)	(2)	(3)	(4)	(5)
Epected return (standardised)	0.071				0.055
Altruism ORP (standardised)		0.094			0.078
Risk tolerance			0.009		0.006
Individual characteristics				0.009	0.017

Note: The table shows Shapley values for potential determinants of trust. Each column corresponds to a model in Table G9 and cells show Shapley values for key variables of interest. For instance, column 1 shows shapley values for expected returns (i.e. the variable included in Model 1 in Table G9). Grouped Shapley values are shown for individual characteristics.

Table G11: Correlates of the amount sent in the UK (OLS regressions)

VARIABLES	(1)	(2)	(3)	(4)	(5)
Expected return (standardised)	0.937***				0.778***
	(0.090)				(0.091)
Altruism (standardised)		0.827***			0.662***
		(0.092)			(0.091)
Risk proclivity			0.142**		0.062
			(0.057)		(0.054)
Female				-0.405**	-0.352**
				(0.189)	(0.175)
Non-tertiary diploma				0.120	0.164
				(0.269)	(0.250)
University				0.154	0.188
				(0.210)	(0.195)
Age				0.004	0.003
				(0.007)	(0.007)
Respondent born in country				-0.190	-0.119
				(0.307)	(0.286)
Household size				-0.147*	-0.136*
				(0.078)	(0.072)
Rural				0.140	0.146
				(0.218)	(0.203)
Working				0.025	-0.079
				(0.198)	(0.184)
Religion important				-0.179	-0.298
				(0.214)	(0.199)
Completely satisfied with life				0.285	-0.068
				(0.263)	(0.246)
Observations	1,034	1,034	1,034	1,034	1,034
R-squared	0.095	0.073	0.006	0.013	0.151

Note: Results are from OLS regression models. The dependent variable in all models is the amount sent by the first mover.

Table G12: Potential determinants of trusting behaviour in the UK (Shapley values)

	Shapley values				
	(1)	(2)	(3)	(4)	(5)
Expected return (standardised)	0.095				0.077
Altruism ORP (standardised)		0.073			0.058
Risk tolerance			0.006		0.003
Individual characteristics				0.006	0.013

Note: The table shows Shapley values for potential determinants of trust. Each column corresponds to a model in Table G11 and cells show Shapley values for key variables of interest. For instance, column 1 shows shapley values for expected returns (i.e. the variable included in Model 1 in Table G11). Grouped Shapley values are shown for individual characteristics.

Table G13: Correlates of the amount sent in Italy (OLS regressions)

VARIABLES	(1)	(2)	(3)	(4)	(5)
Expected return (standardised)	0.722***				0.458***
	(0.084)				(0.086)
Altruism (standardised)		1.142***			0.948***
		(0.094)			(0.099)
Risk proclivity			0.093		0.017
			(0.058)		(0.054)
Female				-0.590***	-0.464***
				(0.190)	(0.176)
Non-tertiary diploma				0.214	0.316
				(0.263)	(0.243)
University				-0.052	0.095
				(0.214)	(0.198)
Age				-0.010	-0.012*
				(0.008)	(0.007)
Respondent born in country				0.030	-0.433
				(0.497)	(0.460)
Household size				-0.101	-0.080
				(0.080)	(0.074)
Rural				-0.170	-0.201
				(0.186)	(0.172)
Working				0.251	0.157
				(0.209)	(0.193)
Religion important				-0.128	-0.173
				(0.191)	(0.177)
Completely satisfied with life				0.096	-0.181
				(0.264)	(0.245)
Observations	998	998	998	998	998
R-squared	0.070	0.129	0.003	0.020	0.169

Note: Results are from OLS regression models. The dependent variable in all models is the amount sent by the first mover.

Table G14: Potential determinants of trusting behaviour in Italy (Shapley values)

	Shapley values				
	(1)	(2)	(3)	(4)	(5)
Expected return (standardised)	0.070				0.046
Altruism ORP (standardised)		0.129			0.102
Risk tolerance			0.003		0.001
Individual characteristics				0.003	0.019

Note: The table shows Shapley values for potential determinants of trust. Each column corresponds to a model in Table G13 and cells show Shapley values for key variables of interest. For instance, column 1 shows shapley values for expected returns (i.e. the variable included in Model 1 in Table G13). Grouped Shapley values are shown for individual characteristics.

Table G15: Correlates of the amount sent in Japan (OLS regressions)

VARIABLES	(1)	(2)	(3)	(4)	(5)
Expected return (standardised)	0.860***				0.420***
	(0.073)				(0.071)
Altruism (standardised)		1.288***			1.157***
		(0.061)			(0.063)
Risk proclivity			0.297***		0.250***
			(0.041)		(0.037)
Female				-0.612***	-0.387***
				(0.146)	(0.132)
Non-tertiary diploma				0.503**	0.573***
				(0.241)	(0.216)
University				0.083	0.243*
				(0.162)	(0.145)
Age				0.013**	0.008
				(0.006)	(0.005)
Respondent born in country				-1.104	-1.031
				(1.640)	(1.466)
Household size				0.024	-0.005
				(0.055)	(0.049)
Rural				-0.120	-0.228
				(0.356)	(0.318)
Working				0.042	-0.100
				(0.157)	(0.141)
Religion important				0.253	-0.100
				(0.175)	(0.157)
Completely satisfied with life				0.040	-0.204
				(0.292)	(0.261)
Observations	2,154	2,154	2,154	2,154	2,154
R-squared	0.061	0.174	0.024	0.016	0.215

Note: Results are from OLS regression models. The dependent variable in all models is the amount sent by the first mover.

Table G16: Potential determinants of trusting behaviour in Japan (Shapley values)

	Shapley values				
	(1)	(2)	(3)	(4)	(5)
Expected return (standardised)	0.061				0.036
Altruism ORP (standardised)		0.174			0.148
Risk tolerance			0.024		0.020
Individual characteristics				0.024	0.011

Note: The table shows Shapley values for potential determinants of trust. Each column corresponds to a model in Table G15 and cells show Shapley values for key variables of interest. For instance, column 1 shows shapley values for expected returns (i.e. the variable included in Model 1 in Table G15). Grouped Shapley values are shown for individual characteristics.

Table G17: Correlates of the amount sent in Luxembourg (OLS regressions)

VARIABLES	(1)	(2)	(3)	(4)	(5)
Expected return (standardised)	0.554***				0.457***
	(0.096)				(0.094)
Altruism (standardised)		0.818***			0.760***
		(0.097)			(0.097)
Risk proclivity			0.185***		0.126**
			(0.053)		(0.050)
Female				-0.839***	-0.708***
				(0.185)	(0.177)
Non-tertiary diploma				-0.085	-0.059
				(0.263)	(0.251)
University				0.380*	0.551***
				(0.215)	(0.206)
Age				-0.010	-0.012*
				(0.007)	(0.006)
Respondent born in country				0.044	0.125
				(0.190)	(0.181)
Household size				0.052	0.027
				(0.070)	(0.067)
Rural				-0.174	-0.105
				(0.287)	(0.273)
Working				0.309	0.230
				(0.216)	(0.206)
Religion important				-0.053	-0.288
				(0.229)	(0.218)
Completely satisfied with life				0.477*	0.284
				(0.281)	(0.268)
Observations	981	981	981	981	981
R-squared	0.033	0.067	0.012	0.039	0.135

Note: Results are from OLS regression models. The dependent variable in all models is the amount sent by the first mover.

Table G18: Potential determinants of trusting behaviour in Luxembourg (Shapley values)

	Shapley values				
	(1)	(2)	(3)	(4)	(5)
Expected return (standardised)	0.033				0.027
Altruism ORP (standardised)		0.067			0.061
Risk tolerance			0.012		0.009
Individual characteristics				0.012	0.038

Note: The table shows Shapley values for potential determinants of trust. Each column corresponds to a model in Table G17 and cells show Shapley values for key variables of interest. For instance, column 1 shows shapley values for expected returns (i.e. the variable included in Model 1 in Table G17). Grouped Shapley values are shown for individual characteristics.

Table G19: Correlates of the amount sent in the USA (OLS regressions)

VARIABLES	(1)	(2)	(3)	(4)	(5)
Expected return (standardised)	0.691***				0.403***
	(0.085)				(0.085)
Altruism (standardised)		1.061***			0.934***
		(0.083)			(0.086)
Risk proclivity			0.165***		0.110**
			(0.052)		(0.048)
Female				-0.408**	-0.338*
				(0.189)	(0.174)
Non-tertiary diploma				0.609**	0.638***
				(0.258)	(0.238)
University				0.648**	0.704***
				(0.262)	(0.241)
Age				-0.002	-0.004
				(0.007)	(0.007)
Respondent born in country				-0.059	0.122
				(0.324)	(0.299)
Household size				0.010	-0.027
				(0.076)	(0.070)
Rural				0.196	0.121
				(0.225)	(0.207)
Working				-0.158	-0.293
				(0.203)	(0.187)
Religion important				-0.215	-0.285
				(0.192)	(0.177)
Completely satisfied with life				0.327	0.107
				(0.251)	(0.231)
Observations	1,071	1,071	1,071	1,071	1,071
R-squared	0.059	0.132	0.009	0.014	0.170

Note: Results are from OLS regression models. The dependent variable in all models is the amount sent by the first mover.

Table G20: Potential determinants of trusting behaviour in Luxembourg (Shapley values)

	Shapley values				
	(1)	(2)	(3)	(4)	(5)
Expected return (standardised)	0.059				0.038
Altruism ORP (standardised)		0.132			0.112
Risk tolerance			0.009		0.006
Individual characteristics				0.009	0.014

Note: The table shows Shapley values for potential determinants of trust. Each column corresponds to a model in Table G19 and cells show Shapley values for key variables of interest. For instance, column 1 shows shapley values for expected returns (i.e. the variable included in Model 1 in Table G19). Grouped Shapley values are shown for individual characteristics.

G.4 Country-specific regressions: Proportion returned

Table G21: Determinants of the proportion returned in Germany (OLS regressions)

VARIABLES	(1)<br>0 CU	(2)<br>1 CU	(3)<br>2 CU	(4)<br>3 CU	(5)<br>4 CU	(6)<br>5 CU	(7)<br>6 CU	(8)<br>7 CU	(9)<br>8 CU	(10)<br>9 CU	(11)<br>10 CU
Conditional cooperation	-0.086***	-0.060***	-0.046***	-0.030***	-0.020***	-0.013**	-0.004	0.001	0.003	0.006	0.008
	(0.008)	(0.006)	(0.006)	(0.006)	(0.006)	(0.006)	(0.006)	(0.006)	(0.006)	(0.006)	(0.006)
Altruism ORP (standardised)	0.085***	0.067***	0.063***	0.066***	0.064***	0.064***	0.060***	0.064***	0.058***	0.057***	0.059***
	(0.009)	(0.007)	(0.006)	(0.006)	(0.007)	(0.007)	(0.007)	(0.007)	(0.007)	(0.007)	(0.007)
Risk proclivity	0.005	0.001	0.002	0.002	-0.002	-0.000	-0.001	0.001	0.001	-0.002	0.001
	(0.005)	(0.004)	(0.003)	(0.003)	(0.004)	(0.003)	(0.004)	(0.004)	(0.004)	(0.004)	(0.004)
Female	0.037**	0.027**	0.024**	0.019*	0.016	0.004	0.007	0.004	0.000	-0.009	-0.002
	(0.017)	(0.013)	(0.012)	(0.012)	(0.012)	(0.012)	(0.012)	(0.013)	(0.013)	(0.013)	(0.013)
Non-tertiary diploma	-0.027	-0.035**	-0.039***	-0.035**	-0.041***	-0.042***	-0.043***	-0.042***	-0.040**	-0.037**	-0.035**
	(0.022)	(0.016)	(0.014)	(0.014)	(0.015)	(0.015)	(0.015)	(0.016)	(0.016)	(0.016)	(0.016)
University	-0.032	-0.031*	-0.034**	-0.027*	-0.025	-0.026	-0.023	-0.020	-0.021	-0.016	-0.011
	(0.022)	(0.016)	(0.015)	(0.015)	(0.016)	(0.016)	(0.016)	(0.016)	(0.016)	(0.017)	(0.017)
Age	0.003***	0.002***	0.001***	0.001***	0.001***	0.001*	0.001*	0.001*	0.001	0.001	0.001
	(0.001)	(0.000)	(0.000)	(0.000)	(0.000)	(0.000)	(0.000)	(0.000)	(0.000)	(0.000)	(0.000)
Respondent born in country	0.010	0.015	0.014	0.006	-0.001	0.004	0.003	0.005	-0.007	0.004	0.012
	(0.036)	(0.026)	(0.024)	(0.024)	(0.025)	(0.025)	(0.025)	(0.026)	(0.026)	(0.027)	(0.026)
Household size	0.003	0.007	0.005	0.005	0.003	0.001	0.003	0.004	0.003	0.006	0.005
	(0.007)	(0.005)	(0.005)	(0.005)	(0.005)	(0.005)	(0.005)	(0.005)	(0.005)	(0.005)	(0.005)
Rural	0.003	0.008	0.002	-0.005	-0.007	-0.005	-0.007	-0.009	-0.009	-0.009	-0.009
	(0.017)	(0.013)	(0.012)	(0.012)	(0.012)	(0.012)	(0.012)	(0.013)	(0.013)	(0.013)	(0.013)
Working	0.005	0.009	0.006	0.003	0.007	0.016	0.018	0.016	0.017	0.011	0.014
	(0.019)	(0.014)	(0.013)	(0.013)	(0.013)	(0.013)	(0.013)	(0.014)	(0.014)	(0.014)	(0.014)
Religion important	0.017	-0.002	0.010	0.005	0.005	0.006	0.010	0.005	0.006	0.009	0.010
	(0.019)	(0.014)	(0.013)	(0.013)	(0.013)	(0.013)	(0.013)	(0.014)	(0.014)	(0.014)	(0.014)
Completely satisfied with life	0.043*	0.026	0.030*	0.011	0.013	0.015	0.016	0.022	0.008	0.011	-0.001
	(0.025)	(0.018)	(0.017)	(0.017)	(0.018)	(0.017)	(0.018)	(0.018)	(0.018)	(0.018)	(0.018)
Observations	998	998	998	998	998	998	998	998	998	998	998
R-squared	0.211	0.213	0.188	0.153	0.118	0.110	0.093	0.096	0.079	0.075	0.080

Note: Results are from OLS regression models. The dependent variable in all models is the proportion of the available amount returned (endowment + tripled amount sent) by the trustee in the investment game.

Table G22: Proportion returned and conditional cooperation in Germany (OLS regressions)

VARIABLES	(1)<br>0 CU	(2)<br>1 CU	(3)<br>2 CU	(4)<br>3 CU	(5)<br>4 CU	(6)<br>5 CU	(7)<br>6 CU	(8)<br>7 CU	(9)<br>8 CU	(10)<br>9 CU	(11)<br>10 CU
Conditional cooperation	-0.100***	-0.069***	-0.054***	-0.038***	-0.027***	-0.019***	-0.011*	-0.006	-0.003	0.001	0.002
	(0.009)	(0.006)	(0.006)	(0.006)	(0.006)	(0.006)	(0.006)	(0.006)	(0.006)	(0.006)	(0.006)
Observations	998	998	998	998	998	998	998	998	998	998	998
R-squared	0.120	0.111	0.081	0.042	0.020	0.010	0.003	0.001	0.000	0.000	0.000

Note: Results are from OLS regression models. The dependent variable in all models is the proportion of the available amount returned (endowment + tripled amount sent) by the trustee in the investment game.

Table G23: Proportion returned and altruism (ORP) in Germany (OLS regressions)

VARIABLES	(1)<br>0 CU	(2)<br>1 CU	(3)<br>2 CU	(4)<br>3 CU	(5)<br>4 CU	(6)<br>5 CU	(7)<br>6 CU	(8)<br>7 CU	(9)<br>8 CU	(10)<br>9 CU	(11)<br>10 CU
Altruism ORP (standardised)	0.096***	0.075***	0.069***	0.070***	0.066***	0.066***	0.061***	0.065***	0.058***	0.057***	0.059***
	(0.010)	(0.007)	(0.007)	(0.006)	(0.007)	(0.006)	(0.007)	(0.007)	(0.007)	(0.007)	(0.007)
Observations	998	998	998	998	998	998	998	998	998	998	998
R-squared	0.086	0.099	0.101	0.107	0.092	0.094	0.080	0.084	0.069	0.065	0.070

Note: Results are from OLS regression models. The dependent variable in all models is the proportion of the available amount returned (endowment + tripled amount sent) by the trustee in the investment game.

Table G24: Determinants of the proportion returned in the UK (OLS regressions)

VARIABLES	(1)<br>0 CU	(2)<br>1 CU	(3)<br>2 CU	(4)<br>3 CU	(5)<br>4 CU	(6)<br>5 CU	(7)<br>6 CU	(8)<br>7 CU	(9)<br>8 CU	(10)<br>9 CU	(11)<br>10 CU
Conditional cooperation	-0.068***	-0.030***	-0.013*	0.005	0.009	0.017***	0.023***	0.029***	0.032***	0.038***	0.038***
	(0.009)	(0.007)	(0.006)	(0.006)	(0.006)	(0.006)	(0.007)	(0.007)	(0.007)	(0.007)	(0.007)
Altruism ORP (standardised)	0.077***	0.070***	0.058***	0.059***	0.056***	0.062***	0.053***	0.058***	0.053***	0.060***	0.064***
	(0.008)	(0.007)	(0.006)	(0.006)	(0.006)	(0.006)	(0.006)	(0.006)	(0.006)	(0.007)	(0.007)
Risk proclivity	0.015***	0.013***	0.011***	0.008**	0.010***	0.010***	0.008**	0.008**	0.010**	0.006	0.009**
	(0.005)	(0.004)	(0.004)	(0.004)	(0.004)	(0.004)	(0.004)	(0.004)	(0.004)	(0.004)	(0.004)
Female	-0.017	-0.004	-0.003	-0.010	-0.010	-0.016	-0.016	-0.017	-0.016	-0.018	-0.014
	(0.017)	(0.014)	(0.012)	(0.012)	(0.012)	(0.012)	(0.012)	(0.013)	(0.013)	(0.013)	(0.013)
Non-tertiary diploma	-0.018	0.003	-0.001	-0.001	-0.010	-0.006	-0.007	-0.010	-0.012	-0.001	-0.002
	(0.024)	(0.019)	(0.017)	(0.017)	(0.017)	(0.017)	(0.018)	(0.018)	(0.018)	(0.018)	(0.018)
University	-0.025	-0.020	-0.005	-0.007	0.002	-0.003	0.012	0.011	0.007	0.010	0.017
	(0.019)	(0.015)	(0.014)	(0.013)	(0.014)	(0.014)	(0.014)	(0.014)	(0.014)	(0.014)	(0.014)
Age	0.002***	0.001	0.000	0.000	-0.000	0.000	-0.000	-0.000	-0.000	-0.000	0.000
	(0.001)	(0.001)	(0.000)	(0.000)	(0.000)	(0.000)	(0.000)	(0.000)	(0.000)	(0.000)	(0.001)
Respondent born in country	0.000	0.008	0.005	0.007	0.006	0.005	0.014	0.001	0.012	0.022	0.013
	(0.027)	(0.022)	(0.020)	(0.019)	(0.020)	(0.020)	(0.020)	(0.021)	(0.020)	(0.021)	(0.021)
Household size	0.011	0.006	0.002	0.003	-0.001	-0.002	-0.001	0.000	-0.003	-0.004	-0.006
	(0.007)	(0.006)	(0.005)	(0.005)	(0.005)	(0.005)	(0.005)	(0.005)	(0.005)	(0.005)	(0.005)
Rural	0.006	0.013	0.026*	0.024*	0.024*	0.026*	0.027*	0.021	0.023	0.014	0.016
	(0.019)	(0.016)	(0.014)	(0.014)	(0.014)	(0.014)	(0.014)	(0.015)	(0.015)	(0.015)	(0.015)
Working	0.006	0.005	-0.003	0.010	0.004	0.001	-0.001	-0.002	0.005	0.004	-0.004
	(0.018)	(0.014)	(0.013)	(0.013)	(0.013)	(0.013)	(0.013)	(0.013)	(0.013)	(0.013)	(0.014)
Religion important	0.024	0.008	0.004	0.005	-0.005	-0.015	-0.016	-0.019	-0.017	-0.018	-0.020
	(0.019)	(0.016)	(0.014)	(0.014)	(0.014)	(0.014)	(0.014)	(0.015)	(0.014)	(0.015)	(0.015)
Completely satisfied with life	0.018	0.015	0.029*	0.023	0.036**	0.034**	0.021	0.029	0.029	0.027	0.024
	(0.023)	(0.019)	(0.017)	(0.017)	(0.017)	(0.017)	(0.017)	(0.018)	(0.018)	(0.018)	(0.018)
Observations	1,034	1,034	1,034	1,034	1,034	1,034	1,034	1,034	1,034	1,034	1,034
R-squared	0.169	0.135	0.108	0.100	0.094	0.110	0.090	0.100	0.097	0.111	0.119

Note: Results are from OLS regression models. The dependent variable in all models is the proportion of the available amount returned (endowment + tripled amount sent) by the trustee in the investment game.

Table G25: Proportion returned and conditional cooperation in the UK (OLS regressions)

VARIABLES	(1)<br>0 CU	(2)<br>1 CU	(3)<br>2 CU	(4)<br>3 CU	(5)<br>4 CU	(6)<br>5 CU	(7)<br>6 CU	(8)<br>7 CU	(9)<br>8 CU	(10)<br>9 CU	(11)<br>10 CU
Conditional cooperation	-0.082***	-0.039***	-0.020***	-0.003	0.003	0.010	0.018***	0.025***	0.028***	0.034***	0.033***
	(0.009)	(0.007)	(0.007)	(0.006)	(0.007)	(0.007)	(0.007)	(0.007)	(0.007)	(0.007)	(0.007)
Observations	1,034	1,034	1,034	1,034	1,034	1,034	1,034	1,034	1,034	1,034	1,034
R-squared	0.073	0.026	0.009	0.000	0.000	0.002	0.008	0.013	0.016	0.023	0.021

Note: Results are from OLS regression models. The dependent variable in all models is the proportion of the available amount returned (endowment + tripled amount sent) by the trustee in the investment game.

Table G26: Proportion returned and altruism (ORP) in the UK (OLS regressions)

VARIABLES	(1)<br>0 CU	(2)<br>1 CU	(3)<br>2 CU	(4)<br>3 CU	(5)<br>4 CU	(6)<br>5 CU	(7)<br>6 CU	(8)<br>7 CU	(9)<br>8 CU	(10)<br>9 CU	(11)<br>10 CU
Altruism ORP (standardised)	0.084***	0.074***	0.061***	0.061***	0.058***	0.062***	0.053***	0.057***	0.052***	0.058***	0.062***
	(0.009)	(0.007)	(0.006)	(0.006)	(0.006)	(0.006)	(0.006)	(0.006)	(0.006)	(0.007)	(0.007)
Observations	1,034	1,034	1,034	1,034	1,034	1,034	1,034	1,034	1,034	1,034	1,034
R-squared	0.081	0.100	0.087	0.088	0.078	0.089	0.065	0.070	0.060	0.071	0.078

Note: Results are from OLS regression models. The dependent variable in all models is the proportion of the available amount returned (endowment + tripled amount sent) by the trustee in the investment game.

Table G27: Determinants of the Proportion returned in the investment game in Italy (OLS regressions)

VARIABLES	(1)<br>0 CU	(2)<br>1 CU	(3)<br>2 CU	(4)<br>3 CU	(5)<br>4 CU	(6)<br>5 CU	(7)<br>6 CU	(8)<br>7 CU	(9)<br>8 CU	(10)<br>9 CU	(11)<br>10 CU
Conditional cooperation	-0.056***	-0.028***	-0.012*	-0.001	0.005	0.010	0.013*	0.019***	0.023***	0.030***	0.031***
	(0.009)	(0.007)	(0.006)	(0.006)	(0.006)	(0.006)	(0.006)	(0.007)	(0.007)	(0.007)	(0.007)
Altruism ORP (standardised)	0.102***	0.096***	0.086***	0.080***	0.084***	0.083***	0.077***	0.081***	0.080***	0.081***	0.078***
	(0.010)	(0.007)	(0.007)	(0.007)	(0.007)	(0.007)	(0.007)	(0.007)	(0.007)	(0.008)	(0.008)
Risk proclivity	0.007	0.005	0.003	0.001	0.001	-0.000	-0.001	-0.001	0.000	-0.001	0.001
	(0.006)	(0.004)	(0.004)	(0.004)	(0.004)	(0.004)	(0.004)	(0.004)	(0.004)	(0.004)	(0.004)
Female	0.033*	0.027*	0.014	0.011	0.005	0.001	-0.002	-0.012	-0.014	-0.019	-0.024*
	(0.018)	(0.014)	(0.013)	(0.013)	(0.013)	(0.013)	(0.013)	(0.014)	(0.014)	(0.014)	(0.014)
Non-tertiary diploma	-0.039	-0.011	0.003	0.012	0.012	0.015	0.005	0.005	0.002	0.007	0.008
	(0.025)	(0.019)	(0.018)	(0.018)	(0.018)	(0.018)	(0.019)	(0.019)	(0.019)	(0.020)	(0.020)
University	-0.012	0.006	0.019	0.011	0.019	0.019	0.014	0.012	0.015	0.016	0.015
	(0.021)	(0.016)	(0.015)	(0.014)	(0.014)	(0.015)	(0.015)	(0.016)	(0.016)	(0.016)	(0.016)
Age	0.002*	0.000	-0.000	-0.000	-0.001**	-0.001*	-0.001*	-0.001**	-0.001**	-0.001**	-0.001**
	(0.001)	(0.001)	(0.001)	(0.001)	(0.001)	(0.001)	(0.001)	(0.001)	(0.001)	(0.001)	(0.001)
Respondent born in country	-0.083*	-0.074**	-0.023	-0.025	-0.013	-0.014	-0.035	-0.014	-0.006	0.003	0.018
	(0.048)	(0.037)	(0.034)	(0.033)	(0.034)	(0.034)	(0.035)	(0.036)	(0.036)	(0.037)	(0.037)
Household size	-0.004	-0.002	-0.004	-0.004	-0.002	-0.006	-0.002	-0.002	-0.003	-0.003	-0.004
	(0.008)	(0.006)	(0.005)	(0.005)	(0.005)	(0.006)	(0.006)	(0.006)	(0.006)	(0.006)	(0.006)
Rural	-0.035*	-0.013	-0.011	-0.010	-0.012	-0.008	-0.008	-0.004	-0.009	-0.003	-0.005
	(0.018)	(0.014)	(0.013)	(0.012)	(0.013)	(0.013)	(0.013)	(0.013)	(0.014)	(0.014)	(0.014)
Working	0.030	0.022	0.012	0.011	0.011	0.005	0.006	0.005	-0.002	0.003	0.005
	(0.020)	(0.015)	(0.014)	(0.014)	(0.014)	(0.014)	(0.015)	(0.015)	(0.015)	(0.016)	(0.016)
Religion important	0.038**	0.024*	0.021	0.018	0.012	0.011	0.004	0.006	0.008	0.002	0.000
	(0.018)	(0.014)	(0.013)	(0.013)	(0.013)	(0.013)	(0.013)	(0.014)	(0.014)	(0.014)	(0.014)
Completely satisfied with life	-0.032	-0.022	-0.024	-0.016	-0.014	-0.020	-0.019	-0.019	-0.022	-0.018	-0.021
	(0.025)	(0.020)	(0.018)	(0.018)	(0.018)	(0.018)	(0.019)	(0.019)	(0.019)	(0.020)	(0.020)
Observations	998	998	998	998	998	998	998	998	998	998	998
R-squared	0.171	0.176	0.149	0.128	0.140	0.133	0.114	0.124	0.125	0.131	0.127

Note: Results are from OLS regression models. The dependent variable in all models is the proportion of the available amount returned (endowment + tripled amount sent) by the trustee in the investment game.

Table G28: Proportion returned and conditional cooperation in Italy (OLS regressions)

VARIABLES	(1)<br>0 CU	(2)<br>1 CU	(3)<br>2 CU	(4)<br>3 CU	(5)<br>4 CU	(6)<br>5 CU	(7)<br>6 CU	(8)<br>7 CU	(9)<br>8 CU	(10)<br>9 CU	(11)<br>10 CU
Conditional cooperation	-0.070***	-0.037***	-0.018***	-0.005	0.002	0.007	0.011	0.017**	0.021***	0.028***	0.030***
	(0.009)	(0.007)	(0.007)	(0.006)	(0.006)	(0.007)	(0.007)	(0.007)	(0.007)	(0.007)	(0.007)
Observations	998	998	998	998	998	998	998	998	998	998	998
R-squared	0.055	0.026	0.007	0.001	0.000	0.001	0.003	0.006	0.009	0.016	0.017

Note: Results are from OLS regression models. The dependent variable in all models is the proportion of the available amount returned (endowment + tripled amount sent) by the trustee in the investment game.

Table G29: Proportion returned and altruism (ORP) in Italy (OLS regressions)

VARIABLES	(1)<br>0 CU	(2)<br>1 CU	(3)<br>2 CU	(4)<br>3 CU	(5)<br>4 CU	(6)<br>5 CU	(7)<br>6 CU	(8)<br>7 CU	(9)<br>8 CU	(10)<br>9 CU	(11)<br>10 CU
Altruism ORP (standardised)	0.106***	0.097***	0.086***	0.078***	0.082***	0.081***	0.074***	0.079***	0.078***	0.078***	0.076***
	(0.010)	(0.007)	(0.007)	(0.007)	(0.007)	(0.007)	(0.007)	(0.007)	(0.007)	(0.008)	(0.008)
Observations	998	998	998	998	998	998	998	998	998	998	998
R-squared	0.102	0.145	0.137	0.122	0.131	0.121	0.101	0.105	0.102	0.098	0.092

Note: Results are from OLS regression models. The dependent variable in all models is the proportion of the available amount returned (endowment + tripled amount sent) by the trustee in the investment game.

Table G30: Determinants of the proportion returned in Japan (OLS regressions)

VARIABLES	(1)<br>0 CU	(2)<br>1 CU	(3)<br>2 CU	(4)<br>3 CU	(5)<br>4 CU	(6)<br>5 CU	(7)<br>6 CU	(8)<br>7 CU	(9)<br>8 CU	(10)<br>9 CU	(11)<br>10 CU
Conditional cooperation	-0.053***	-0.028***	-0.012***	-0.004	0.005	0.011**	0.017***	0.023***	0.025***	0.029***	0.029***
	(0.006)	(0.005)	(0.004)	(0.004)	(0.004)	(0.004)	(0.004)	(0.004)	(0.004)	(0.004)	(0.004)
Altruism ORP (standardised)	0.104***	0.076***	0.069***	0.066***	0.062***	0.061***	0.061***	0.059***	0.058***	0.057***	0.056***
	(0.005)	(0.004)	(0.004)	(0.004)	(0.004)	(0.004)	(0.004)	(0.004)	(0.004)	(0.004)	(0.004)
Risk proclivity	0.000	0.003	0.003	0.005**	0.003	0.004*	0.004*	0.005**	0.005**	0.005**	0.006**
	(0.003)	(0.002)	(0.002)	(0.002)	(0.002)	(0.002)	(0.002)	(0.002)	(0.002)	(0.002)	(0.002)
Female	0.018	-0.001	-0.012	-0.014*	-0.018**	-0.020**	-0.022***	-0.022***	-0.025***	-0.025***	-0.025***
	(0.012)	(0.009)	(0.008)	(0.008)	(0.008)	(0.008)	(0.008)	(0.008)	(0.008)	(0.008)	(0.009)
Non-tertiary diploma	0.023	0.031**	0.027**	0.023*	0.023*	0.025*	0.015	0.005	0.005	0.003	0.002
	(0.019)	(0.014)	(0.013)	(0.013)	(0.013)	(0.013)	(0.013)	(0.013)	(0.013)	(0.013)	(0.014)
University	-0.003	0.014	0.017**	0.012	0.015*	0.018**	0.014	0.015*	0.015*	0.014	0.016*
	(0.013)	(0.010)	(0.009)	(0.009)	(0.009)	(0.009)	(0.009)	(0.009)	(0.009)	(0.009)	(0.009)
Age	0.002***	0.001***	0.001**	0.001*	0.000	0.000	-0.000	-0.000	-0.000	-0.000	-0.000
	(0.000)	(0.000)	(0.000)	(0.000)	(0.000)	(0.000)	(0.000)	(0.000)	(0.000)	(0.000)	(0.000)
Respondent born in country	-0.083	-0.064	-0.043	-0.025	-0.131	-0.153*	-0.138	-0.119	-0.098	-0.081	-0.115
	(0.130)	(0.098)	(0.088)	(0.087)	(0.090)	(0.090)	(0.087)	(0.092)	(0.091)	(0.092)	(0.094)
Household size	-0.003	-0.004	-0.002	-0.002	-0.001	-0.001	-0.001	-0.001	0.001	-0.000	0.001
	(0.004)	(0.003)	(0.003)	(0.003)	(0.003)	(0.003)	(0.003)	(0.003)	(0.003)	(0.003)	(0.003)
Rural	0.008	0.023	0.020	0.019	0.017	0.020	0.001	-0.000	-0.002	0.005	0.007
	(0.028)	(0.021)	(0.019)	(0.019)	(0.019)	(0.020)	(0.019)	(0.020)	(0.020)	(0.020)	(0.020)
Working	0.025**	0.004	0.003	-0.002	-0.006	-0.007	-0.007	-0.008	-0.007	-0.008	-0.007
	(0.012)	(0.009)	(0.008)	(0.008)	(0.009)	(0.009)	(0.008)	(0.009)	(0.009)	(0.009)	(0.009)
Religion important	0.038***	0.024**	0.015	0.017*	0.013	0.014	0.015	0.004	0.001	0.004	0.004
	(0.014)	(0.010)	(0.009)	(0.009)	(0.010)	(0.010)	(0.009)	(0.010)	(0.010)	(0.010)	(0.010)
Completely satisfied with life	0.024	0.042**	0.031**	0.025	0.024	0.018	0.022	0.029*	0.027*	0.028*	0.020
	(0.023)	(0.017)	(0.016)	(0.015)	(0.016)	(0.016)	(0.015)	(0.016)	(0.016)	(0.016)	(0.017)
Observations	2,154	2,154	2,154	2,154	2,154	2,154	2,154	2,154	2,154	2,154	2,154
R-squared	0.195	0.172	0.164	0.155	0.134	0.130	0.144	0.130	0.129	0.131	0.122

Note: Results are from OLS regression models. The dependent variable in all models is the proportion of the available amount returned (endowment + tripled amount sent) by the trustee in the investment game.

Table G31: Proportion returned and conditional cooperation in Japan (OLS regressions)

VARIABLES	(1)<br>0 CU	(2)<br>1 CU	(3)<br>2 CU	(4)<br>3 CU	(5)<br>4 CU	(6)<br>5 CU	(7)<br>6 CU	(8)<br>7 CU	(9)<br>8 CU	(10)<br>9 CU	(11)<br>10 CU
Conditional cooperation	-0.047***	-0.024***	-0.009**	-0.001	0.008*	0.014***	0.020***	0.025***	0.028***	0.031***	0.031***
	(0.007)	(0.005)	(0.005)	(0.004)	(0.005)	(0.005)	(0.004)	(0.005)	(0.005)	(0.005)	(0.005)
Observations	2,154	2,154	2,154	2,154	2,154	2,154	2,154	2,154	2,154	2,154	2,154
R-squared	0.022	0.011	0.002	0.000	0.002	0.004	0.010	0.014	0.017	0.022	0.020

Note: Results are from OLS regression models. The dependent variable in all models is the proportion of the available amount returned (endowment + tripled amount sent) by the trustee in the investment game.

Table G32: Proportion returned and altruism (ORP) in Japan (OLS regressions)

VARIABLES	(1)<br>0 CU	(2)<br>1 CU	(3)<br>2 CU	(4)<br>3 CU	(5)<br>4 CU	(6)<br>5 CU	(7)<br>6 CU	(8)<br>7 CU	(9)<br>8 CU	(10)<br>9 CU	(11)<br>10 CU
Altruism ORP (standardised)	0.104***	0.076***	0.069***	0.067***	0.064***	0.062***	0.063***	0.061***	0.060***	0.060***	0.059***
	(0.005)	(0.004)	(0.004)	(0.004)	(0.004)	(0.004)	(0.004)	(0.004)	(0.004)	(0.004)	(0.004)
Observations	2,154	2,154	2,154	2,154	2,154	2,154	2,154	2,154	2,154	2,154	2,154
R-squared	0.148	0.143	0.148	0.144	0.125	0.118	0.129	0.111	0.108	0.106	0.097

Note: Results are from OLS regression models. The dependent variable in all models is the proportion of the available amount returned (endowment + tripled amount sent) by the trustee in the investment game.

Table G33: Determinants of the proportion returned in Luxembourg (OLS regressions)

VARIABLES	(1)<br>0 CU	(2)<br>1 CU	(3)<br>2 CU	(4)<br>3 CU	(5)<br>4 CU	(6)<br>5 CU	(7)<br>6 CU	(8)<br>7 CU	(9)<br>8 CU	(10)<br>9 CU	(11)<br>10 CU
Conditional cooperation	-0.065***	-0.040***	-0.025***	-0.016***	-0.011*	-0.003	-0.004	0.003	0.005	0.010	0.009
	(0.009)	(0.006)	(0.006)	(0.006)	(0.006)	(0.006)	(0.006)	(0.006)	(0.006)	(0.006)	(0.006)
Altruism ORP (standardised)	0.097***	0.080***	0.067***	0.061***	0.054***	0.054***	0.049***	0.048***	0.046***	0.043***	0.048***
	(0.009)	(0.007)	(0.007)	(0.006)	(0.007)	(0.007)	(0.007)	(0.007)	(0.007)	(0.007)	(0.007)
Risk proclivity	0.010**	0.006*	0.006*	0.007**	0.007**	0.007**	0.006*	0.007**	0.007**	0.008**	0.006
	(0.005)	(0.004)	(0.003)	(0.003)	(0.003)	(0.003)	(0.003)	(0.004)	(0.004)	(0.004)	(0.004)
Female	0.025	0.016	0.010	0.012	0.007	0.006	-0.010	-0.013	-0.015	-0.018	-0.029**
	(0.017)	(0.013)	(0.012)	(0.012)	(0.012)	(0.012)	(0.012)	(0.012)	(0.012)	(0.013)	(0.013)
Non-tertiary diploma	0.002	-0.003	-0.007	-0.010	-0.001	-0.009	0.010	-0.001	0.004	-0.002	0.018
	(0.025)	(0.018)	(0.017)	(0.017)	(0.017)	(0.017)	(0.017)	(0.017)	(0.018)	(0.018)	(0.018)
University	-0.060***	-0.041***	-0.028*	-0.017	-0.008	-0.006	0.007	0.005	0.013	0.013	0.017
	(0.020)	(0.015)	(0.014)	(0.014)	(0.014)	(0.014)	(0.014)	(0.014)	(0.014)	(0.015)	(0.015)
Age	0.003***	0.001*	0.000	0.000	-0.000	-0.001	-0.000	-0.001	-0.001*	-0.001	-0.001*
	(0.001)	(0.000)	(0.000)	(0.000)	(0.000)	(0.000)	(0.000)	(0.000)	(0.000)	(0.000)	(0.000)
Respondent born in country	-0.016	-0.009	-0.008	-0.017	-0.016	-0.020	-0.011	-0.014	-0.016	-0.016	-0.014
	(0.018)	(0.013)	(0.012)	(0.012)	(0.012)	(0.012)	(0.012)	(0.013)	(0.013)	(0.013)	(0.013)
Household size	0.011*	0.002	0.001	-0.002	-0.004	-0.003	-0.001	-0.003	-0.004	-0.003	-0.002
	(0.007)	(0.005)	(0.005)	(0.004)	(0.005)	(0.005)	(0.005)	(0.005)	(0.005)	(0.005)	(0.005)
Rural	-0.013	0.009	-0.005	0.011	0.010	0.018	-0.005	0.018	0.013	0.013	0.009
	(0.027)	(0.020)	(0.019)	(0.018)	(0.019)	(0.019)	(0.019)	(0.019)	(0.019)	(0.019)	(0.020)
Working	0.022	0.030**	0.024*	0.012	0.013	0.005	0.001	-0.003	-0.006	-0.003	-0.005
	(0.020)	(0.015)	(0.014)	(0.014)	(0.014)	(0.014)	(0.014)	(0.014)	(0.014)	(0.014)	(0.015)
Religion important	0.002	0.027*	0.008	0.002	-0.006	-0.016	-0.016	-0.024	-0.020	-0.024	-0.020
	(0.021)	(0.016)	(0.015)	(0.015)	(0.015)	(0.015)	(0.015)	(0.015)	(0.015)	(0.015)	(0.016)
Completely satisfied with life	0.037	0.022	0.016	0.015	0.024	0.026	0.023	0.014	0.016	0.024	0.026
	(0.026)	(0.020)	(0.019)	(0.018)	(0.018)	(0.018)	(0.018)	(0.019)	(0.019)	(0.019)	(0.019)
Observations	981	981	981	981	981	981	981	981	981	981	981
R-squared	0.226	0.204	0.144	0.117	0.086	0.081	0.068	0.064	0.063	0.059	0.066

Note: Results are from OLS regression models. The dependent variable in all models is the proportion of the available amount returned (endowment + tripled amount sent) by the trustee in the investment game.

Table G34: Proportion returned and conditional cooperation in Luxembourg (OLS regressions)

VARIABLES	(1)<br>0 CU	(2)<br>1 CU	(3)<br>2 CU	(4)<br>3 CU	(5)<br>4 CU	(6)<br>5 CU	(7)<br>6 CU	(8)<br>7 CU	(9)<br>8 CU	(10)<br>9 CU	(11)<br>10 CU
Conditional cooperation	-0.091***	-0.056***	-0.036***	-0.026***	-0.018***	-0.009	-0.009	-0.002	0.002	0.007	0.006
	(0.009)	(0.006)	(0.006)	(0.006)	(0.006)	(0.006)	(0.006)	(0.006)	(0.006)	(0.006)	(0.006)
Observations	981	981	981	981	981	981	981	981	981	981	981
R-squared	0.101	0.071	0.037	0.020	0.009	0.003	0.002	0.000	0.000	0.001	0.001

Note: Results are from OLS regression models. The dependent variable in all models is the proportion of the available amount returned (endowment + tripled amount sent) by the trustee in the investment game.

Table G35: Proportion returned and altruism (ORP) in Luxembourg (OLS regressions)

VARIABLES	(1)<br>0 CU	(2)<br>1 CU	(3)<br>2 CU	(4)<br>3 CU	(5)<br>4 CU	(6)<br>5 CU	(7)<br>6 CU	(8)<br>7 CU	(9)<br>8 CU	(10)<br>9 CU	(11)<br>10 CU
Altruism ORP (standardised)	0.112***	0.090***	0.073***	0.065***	0.057***	0.055***	0.050***	0.048***	0.046***	0.042***	0.047***
	(0.010)	(0.007)	(0.007)	(0.006)	(0.006)	(0.006)	(0.006)	(0.007)	(0.007)	(0.007)	(0.007)
Observations	981	981	981	981	981	981	981	981	981	981	981
R-squared	0.117	0.137	0.112	0.098	0.073	0.069	0.059	0.052	0.047	0.038	0.046

Note: Results are from OLS regression models. The dependent variable in all models is the proportion of the available amount returned (endowment + tripled amount sent) by the trustee in the investment game.

Table G36: Determinants of the proportion returned in the USA (OLS regressions)

VARIABLES	(1)<br>0 CU	(2)<br>1 CU	(3)<br>2 CU	(4)<br>3 CU	(5)<br>4 CU	(6)<br>5 CU	(7)<br>6 CU	(8)<br>7 CU	(9)<br>8 CU	(10)<br>9 CU	(11)<br>10 CU
Conditional cooperation	-0.073***	-0.046***	-0.022***	-0.005	0.003	0.010*	0.018***	0.021***	0.025***	0.028***	0.033***
	(0.009)	(0.007)	(0.006)	(0.006)	(0.006)	(0.006)	(0.006)	(0.007)	(0.007)	(0.007)	(0.007)
Altruism ORP (standardised)	0.109***	0.086***	0.072***	0.067***	0.068***	0.066***	0.063***	0.059***	0.060***	0.061***	0.059***
	(0.009)	(0.007)	(0.006)	(0.006)	(0.006)	(0.006)	(0.006)	(0.007)	(0.007)	(0.007)	(0.007)
Risk proclivity	0.007	-0.000	-0.000	0.002	-0.000	0.001	0.001	-0.000	0.000	0.000	-0.001
	(0.005)	(0.004)	(0.004)	(0.004)	(0.004)	(0.004)	(0.004)	(0.004)	(0.004)	(0.004)	(0.004)
Female	0.044**	0.021	0.021	0.015	0.017	0.014	0.014	0.006	0.005	-0.003	-0.006
	(0.019)	(0.014)	(0.013)	(0.013)	(0.013)	(0.013)	(0.013)	(0.014)	(0.014)	(0.014)	(0.014)
Non-tertiary diploma	0.001	0.003	0.015	0.019	0.032*	0.032*	0.040**	0.039**	0.048**	0.037*	0.055***
	(0.025)	(0.020)	(0.018)	(0.018)	(0.018)	(0.018)	(0.018)	(0.019)	(0.019)	(0.020)	(0.019)
University	0.012	0.002	0.011	0.018	0.028	0.022	0.040**	0.034*	0.039**	0.032	0.035*
	(0.026)	(0.020)	(0.019)	(0.018)	(0.018)	(0.019)	(0.019)	(0.019)	(0.019)	(0.020)	(0.020)
Age	0.002***	-0.000	-0.000	-0.001**	-0.001*	-0.001	-0.001**	-0.001**	-0.001**	-0.001**	-0.001*
	(0.001)	(0.001)	(0.001)	(0.001)	(0.001)	(0.001)	(0.001)	(0.001)	(0.001)	(0.001)	(0.001)
Respondent born in country	-0.004	0.002	-0.005	-0.022	-0.032	-0.036	-0.031	-0.050**	-0.038	-0.029	-0.039
	(0.032)	(0.025)	(0.023)	(0.022)	(0.023)	(0.023)	(0.023)	(0.024)	(0.024)	(0.025)	(0.024)
Household size	-0.002	-0.003	-0.000	-0.003	-0.001	-0.005	-0.001	-0.002	-0.003	-0.003	-0.002
	(0.007)	(0.006)	(0.005)	(0.005)	(0.005)	(0.005)	(0.005)	(0.006)	(0.006)	(0.006)	(0.006)
Rural	-0.021	-0.010	0.003	0.013	0.010	0.015	0.021	0.016	0.019	0.012	0.006
	(0.022)	(0.017)	(0.016)	(0.016)	(0.016)	(0.016)	(0.016)	(0.017)	(0.017)	(0.017)	(0.017)
Working	-0.008	-0.007	-0.020	-0.019	-0.016	-0.018	-0.022	-0.031**	-0.024	-0.028*	-0.028*
	(0.020)	(0.016)	(0.014)	(0.014)	(0.014)	(0.014)	(0.015)	(0.015)	(0.015)	(0.015)	(0.015)
Religion important	0.009	-0.007	-0.007	-0.004	-0.005	0.001	-0.002	-0.002	-0.002	0.001	0.003
	(0.019)	(0.015)	(0.014)	(0.013)	(0.013)	(0.014)	(0.014)	(0.014)	(0.014)	(0.015)	(0.014)
Completely satisfied with life	0.046*	0.028	0.019	0.013	-0.002	-0.007	-0.013	-0.018	-0.019	-0.020	-0.030
	(0.025)	(0.019)	(0.018)	(0.017)	(0.018)	(0.018)	(0.018)	(0.018)	(0.019)	(0.019)	(0.019)
Observations	1,071	1,071	1,071	1,071	1,071	1,071	1,071	1,071	1,071	1,071	1,071
R-squared	0.205	0.176	0.128	0.110	0.110	0.104	0.100	0.093	0.097	0.093	0.100

Note: Results are from OLS regression models. The dependent variable in all models is the proportion of the available amount returned (endowment + tripled amount sent) by the trustee in the investment game.

Table G37: Proportion returned and conditional cooperation in the USA (OLS regressions)

VARIABLES	(1)<br>0 CU	(2)<br>1 CU	(3)<br>2 CU	(4)<br>3 CU	(5)<br>4 CU	(6)<br>5 CU	(7)<br>6 CU	(8)<br>7 CU	(9)<br>8 CU	(10)<br>9 CU	(11)<br>10 CU
Conditional cooperation	-0.083***	-0.052***	-0.025***	-0.008	0.001	0.008	0.016**	0.020***	0.024***	0.027***	0.032***
	(0.009)	(0.007)	(0.007)	(0.006)	(0.006)	(0.007)	(0.007)	(0.007)	(0.007)	(0.007)	(0.007)
Observations	1,071	1,071	1,071	1,071	1,071	1,071	1,071	1,071	1,071	1,071	1,071
R-squared	0.070	0.046	0.014	0.001	0.000	0.001	0.006	0.009	0.012	0.014	0.020

Note: Results are from OLS regression models. The dependent variable in all models is the proportion of the available amount returned (endowment + tripled amount sent) by the trustee in the investment game.

Table G38: Proportion returned and altruism (ORP) in the USA (OLS regressions)

VARIABLES	(1)<br>0 CU	(2)<br>1 CU	(3)<br>2 CU	(4)<br>3 CU	(5)<br>4 CU	(6)<br>5 CU	(7)<br>6 CU	(8)<br>7 CU	(9)<br>8 CU	(10)<br>9 CU	(11)<br>10 CU
Altruism ORP (standardised)	0.116***	0.089***	0.074***	0.067***	0.067***	0.065***	0.061***	0.057***	0.058***	0.058***	0.056***
	(0.009)	(0.007)	(0.006)	(0.006)	(0.006)	(0.006)	(0.006)	(0.007)	(0.007)	(0.007)	(0.007)
Observations	1,071	1,071	1,071	1,071	1,071	1,071	1,071	1,071	1,071	1,071	1,071
R-squared	0.132	0.136	0.113	0.100	0.098	0.091	0.078	0.065	0.066	0.064	0.060

Note: Results are from OLS regression models. The dependent variable in all models is the proportion of the available amount returned (endowment + tripled amount sent) by the trustee in the investment game.
